# Morphological and Transcriptomic Analysis of a Beetle Chemosensory System Reveals a Gnathal Olfactory Center

**DOI:** 10.1186/s12915-016-0304-z

**Published:** 2016-10-17

**Authors:** Stefan Dippel, Martin Kollmann, Georg Oberhofer, Alice Montino, Carolin Knoll, Milosz Krala, Karl-Heinz Rexer, Sergius Frank, Robert Kumpf, Joachim Schachtner, Ernst A. Wimmer

**Affiliations:** 1 Department of Developmental Biology, Göttingen Center for Molecular Biosciences (GZMB), Georg-August-University Goettingen, Johann-Friedrich-Blumenbach-Institute of Zoology and Anthropology, Ernst-Caspari-Haus, Justus-von-Liebig-Weg 11, 37077 Göttingen, Germany; 2 Department of Biology – Neurobiology/Ethology, Philipps-University Marburg, Karl-von-Frisch-Str. 8, 35032 Marburg, Germany; 3 Department of Evolutionary Developmental Genetics, GZMB, Ernst-Caspari-Haus, Georg-August-University Goettingen, Johann-Friedrich-Blumenbach-Institute of Zoology and Anthropology, Justus-von-Liebig-Weg 11, 37077 Göttingen, Germany; 4 Department of Biology – Mycology, Philipps-University Marburg, Karl-von-Frisch-Str. 8, 35032 Marburg, Germany; 5 Department of Plant Systems Biology, Flanders Institute for Biotechnology, Technologiepark 927, 9052 Gent, Belgium

**Keywords:** *Tribolium castaneum*, olfaction, insect, chemoreception, gustation, neuroanatomy, lobus glomerulatus

## Abstract

**Background:**

The red flour beetle *Tribolium castaneum* is an emerging insect model organism representing the largest insect order, Coleoptera, which encompasses several serious agricultural and forest pests. Despite the ecological and economic importance of beetles, most insect olfaction studies have so far focused on dipteran, lepidopteran, or hymenopteran systems.

**Results:**

Here, we present the first detailed morphological description of a coleopteran olfactory pathway in combination with genome-wide expression analysis of the relevant gene families involved in chemoreception. Our study revealed that besides the antennae, also the mouthparts are highly involved in olfaction and that their respective contribution is processed separately. In this beetle, olfactory sensory neurons from the mouthparts project to the lobus glomerulatus, a structure so far only characterized in hemimetabolous insects, as well as to a so far non-described unpaired glomerularly organized olfactory neuropil in the gnathal ganglion, which we term the gnathal olfactory center. The high number of functional odorant receptor genes expressed in the mouthparts also supports the importance of the maxillary and labial palps in olfaction of this beetle. Moreover, gustatory perception seems equally distributed between antenna and mouthparts, since the number of expressed gustatory receptors is similar for both organs.

**Conclusions:**

Our analysis of the *T. castaneum* chemosensory system confirms that olfactory and gustatory perception are not organotopically separated to the antennae and mouthparts, respectively. The identification of additional olfactory processing centers, the lobus glomerulatus and the gnathal olfactory center, is in contrast to the current picture that in holometabolous insects all olfactory inputs allegedly converge in the antennal lobe. These findings indicate that Holometabola have evolved a wider variety of solutions to chemoreception than previously assumed.

**Electronic supplementary material:**

The online version of this article (doi:10.1186/s12915-016-0304-z) contains supplementary material, which is available to authorized users.

## Background

Insects use chemical cues for most tasks they encounter during their life history. Over long distances, airborne chemical stimuli guide insects to food sources, mates, and places for oviposition [1–6]. Within close range, olfaction as well as gustation are used to discriminate between different food qualities, to avoid toxins or harmful microbes, to communicate intra- or interspecifically, to identify suitable mating partners, and to find appropriate egg-laying sites [6–15]. Because of insects’ devastating impact on agriculture and stored food products, as well as their ability to serve as vectors for detrimental diseases, insect olfaction has become an important research field in biology [4].

Chemical signals are typically perceived within specialized antennal and palpal cuticular structures, the olfactory or gustatory sensilla. These chemosensory sensilla form a hollow structure filled with aqueous lymph and harbor the dendritic branches of the chemosensory neurons (CSNs), namely the olfactory (OSNs) or gustatory sensory neurons (GSNs) [16, 17]. They are divided into several sub-types according to their different morphology [16]. The volatile molecules enter the cavity through wall pores finally to reach and activate the chemoreceptors on the dendrites of the OSNs. To enhance olfactory sensitivity and specificity, odorant binding proteins (OBPs) or potentially chemosensory proteins (CSPs) facilitate the translocation of many, mostly hydrophobic, chemicals through the aqueous lymph [18]. In insects, typically three different receptor families are involved in chemoreception [4]: the ionotropic glutamate-like receptors (IRs) [19, 20], the gustatory receptors (GRs) [13, 21], and the odorant receptors (ORs) [22–24]. The IRs are evolutionarily highly conserved chemoreceptors involved in protostome olfaction [19]. They contain three transmembrane domains and form functional heteromers between an odor-specific IR and a co-receptor (IR8a and IR25a) The GRs are seven transmembrane receptors found across arthropods [25–28] whose quaternary structure [13, 29–31], as well as the signal transduction mechanism [32, 33], are still under debate. The typical ORs are seven transmembrane receptors found in pterygote insects [24] that form functional heteromers with the atypical (general) odorant receptor co-receptor (Orco) [22, 23, 34–36]. Their signal transduction mechanism is currently discussed and they may either form an ionotropic receptor complex that is regulated by second messengers or be functional metabotropic receptors [22, 23, 34, 37–40]. The described influence of G-proteins and affiliated second messengers on insect olfaction supports both mechanisms [41–47]. Moreover, sensitive pheromone detection requires the OR/Orco complex to interact with a sensory neuron membrane protein (SNMP) related to the scavenger receptor CD36 [48–50]. Besides the perireceptor events involved in effective activation, the high temporal resolution of olfactory reception probably also requires signal termination, which is supposedly mediated by secreted or membrane-bound odorant-degrading enzymes (ODEs) [51–55].

Activation of the described chemoreceptors elicits action potentials in the CSNs that are further transmitted via the antennal nerve to the antennal lobe (AL), the first integration center of the olfactory pathway in the brain, or for GSNs, to the primary gustatory center of the gnathal ganglion (GNG) [56]. The AL of insects consists typically of spherical sub-compartments, the olfactory glomeruli [57]. Usually OSNs express only one typical (specific) OR gene and all antennal OSNs expressing the same typical OR converge into the same olfactory glomerulus, creating a chemotropic map-like representation of chemical coding in the AL [58–60], known as the central dogma of olfaction [61, 62]. In *Drosophila melanogaster*, the OR/Orco and IR derived sensory information from the antennae and the maxillary palps is processed in the AL [63], whereas in several hemimetabolous insects, CSNs from the palps converge typically in the lobus glomerulatus (LG), next to but outside the AL [57, 64–66]. In the AL, olfactory information from the OSNs, is processed by a complex network of local interneurons [57, 67–69]. The processed odor information is further relayed by distinct antennal lobe tracts (ALTs) formed by the projection neurons (PNs) to the mushroom body (MB) and the lateral horn (LH) [57, 70]. The MBs are higher-order integration centers for multiple processed sensory information and are responsible for odor discrimination, associative learning, as well as memory storage and retrieval. The LHs receive odor input directly from the ALs or indirectly from the MBs, decode the quality and intensity of the information, and finally trigger immediate odor-driven behavior [71–77].

Despite the evolutionary success and ecological as well as economic importance of beetles [78, 79], little is known on the neuroanatomy, genetics, or biochemistry of their olfactory pathway. Within the Coleoptera, the red flour beetle *Tribolium castaneum* has become the prime model organism for developmental biology and pest management [80]. With its fully annotated genome [81–83] and the multiple powerful genetic tools – such as systemic RNA interference [84, 85], insertional mutagenesis [86], and transgene-based misexpression systems [87, 88] – *T. castaneum* represents an eligible beetle model organism for olfaction. In the current study, we present a substantial overview of the olfactory pathway in *T. castaneum*, covering the morphology of the sensilla and the antenna, all major neuropils including AL, MB, LH, LG, and the gnathal olfactory center (GOC), a previously undescribed glomerularly organized neuropil in the GNG. Additional support for the importance of the gnathal input into olfaction is provided by genome-wide expression analysis of gene families involved in chemoreception (e.g., ORs, GRs, IRs, SMNPs, and ODEs) and CSPs and OBPs, which have recently been published [89].

## Results

### The Antenna of *Tribolium castaneum*

**Fig. 1:**
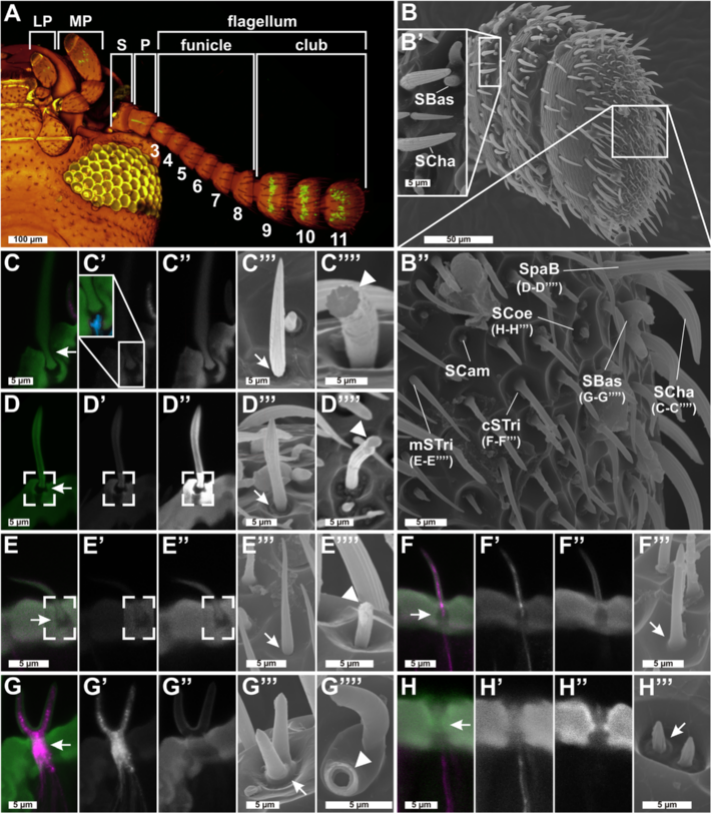
Sensilla types and distribution on *Tribolium castaneum* antennae I. **a** Chemosensory sensilla are restricted to the distal three segments (9–11) of the *T. castaneum* antenna, which is composed of scape (*S*), pedicel (*P*), and flagellum, and the last labial palp (*LP*) and maxillary palp (*MP*) segment. CLSM-stack voltex projection of a transgenic beetle head (ventral view, *green*: partial *Orco*-*Gal4/UAS*-tGFP; yellowish eye, brownish cuticle: autofluorescence). **b**–**b**'' SEM images of the club segments with close-up of segments 9 (**b**') and 11 (**b**''). Single sensilla: CLSM maximum intensity projection overlays (**c**–**h**) of antibody-enhanced *EF1*-*B*-DsRed reporter signal (*magenta*, **c**'–**h**') and cuticle autofluorescence (*green*, **c**''–**h**''). **c**'''–**h**''' SEM analysis. Mechanoreceptive sensilla: *SCam* are small, smooth, and dome-shaped sensilla restricted to segment 11 (Additional file 3: Figure S3a); *SCha* – previously described as spines [90] – are longitudinally corrugated, connected to a neuron at the socket (**c**'; *blue*), jointed (**c**'''; *arrow*), and solid (**c**''''; *arrowhead*). **d**–**d**'''' *SpaB* – in *T. brevicornis* called sensilla squamiformium [95] – resemble modified (slightly thicker tip) *SCha* [96] restricted to segment 11 (Additional file 3: Figure S3b). **e**–**e**'''' *mSTri* (structurally similar to *SCha* but smaller more hair-like appearance) have previously been described in other species [24, 244]. CLSM analysis showed joint-like structures at the base (**c**–**e**, **c**''–**e**'', *open squares*) of the mechanoreceptive sensilla and SEM revealed a small gap at their base (**c**'''**–e**''', *arrow*). Chemoreceptive sensilla: **f**–**f**''' *cSTri* are hair-like structures restricted to segment 11 (Additional file 3: Figure S3d) with a rounded tip and a smooth transition of the base; **g**–**g**'''' *SBas* are smooth-surfaced pegs with rounded tips and smooth transitions at the base (**g**'''; *arrow*). **h**–**h**''' *SCoe* are short and corrugated, and their transition into the antennal cuticle shows a typical elevation (**b**'', **h**'''). All chemoreceptive sensilla (**f**, **g**, **f**'–**h**') house dendritic branches of CSNs labeled by DsRed. The close-up in **c**' shows a non-CSN fiber entering only the base of a *SCha* labeled with phalloidin (*blue*). Chemoreceptive sensilla show a smooth transition into the antennal cuticle (**f**'''–**h**''', *arrow*). Whereas all mechanoreceptive sensilla are solid cuticular structures (fractured in **c**''''–**e**''''), chemoreceptive *SBas* appear hollow (fractured in **g**''''). *CLSM* confocal laser-scanning microscopy, *CSN* chemosensory neuron, *cSTri* chemosensilla trichoidea, *LP* labial palp, *MP* maxillary palp, *mSTri* mechanosensilla trichoidea, *P* pedicel, *S* scape, *SBas* sensilla basiconica, *SCam* sensilla campaniformes, *SCha* sensilla chaetica, *SCoe* sensilla coeloconica, *SEM* scanning electron microscopy, *SpaB* spaculate bristle

**Fig. 2:**
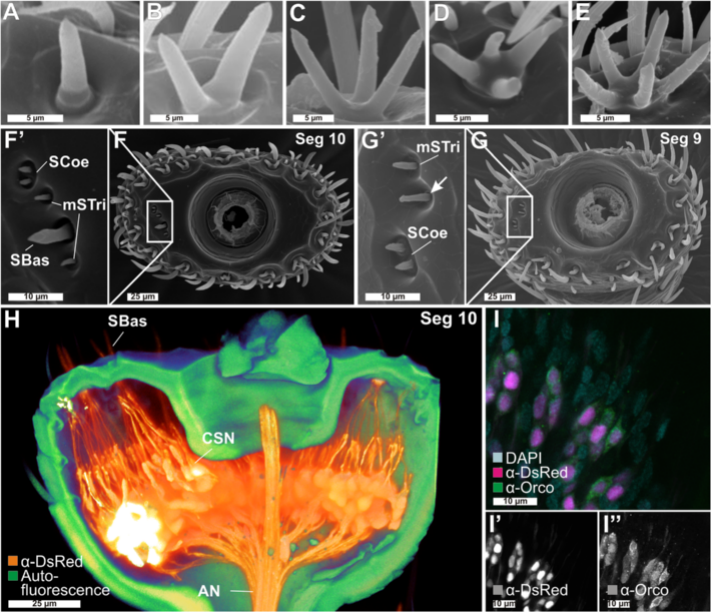
Sensilla types and distribution on *Tribolium castaneum* antennae II. **a**–**e** SEM images of *SBas* with one to five prongs. **f**, **f**' SEM image of the tenth segment of the antenna with a close-up of the lateral corner (**f**') containing *SCoe*, *SBas*, and *mSTri*. **g**, **g**' SEM image of the ninth segment with a close-up of the lateral corner (**g'**) showing *SCoe* and *mSTri*. Chemoreceptive *SCoe* were previously described as “minute spicule-like sensilla trichoidea” [90], are relatively rare (Additional file 3: Figure S3e), and located besides the lateral corners of segments 9 and 10 (**f**',**g**') mostly at the apical side of segment 11 (Fig. 1b''). Chemoreceptive *SBas* are arranged in an axial ring at the distal margins of all three club segments (**f, g**, Fig. 1b–b''). For mechanoreceptive *mSTri*, we identified about 37 on the apical side of segment 11 (Fig. 1b'') and four in lateral corners of segments 9 and 10 (**f**, **f**', **g**, **g**', and Additional file 3: Figure S3c, h). **h** Voltex projection based on a CLSM image stack of the tenth segment from the *EF1*-*B*-DsRed line displaying CSNs (*orange*) and autofluorescence of the cuticle (*green*). The dendrites of the CSNs converge into the *SBas* (on average, six per prong), while the axons unite at the center of the segment and join the antennal nerve (*AN*). **i**–**i**'' Overlay of the signals of the DsRed reporter (*magenta*, **i**') and the Orco antibody (*green*, **i**'') together with DAPI staining (*light blue*) in the *EF1*-*B*-DsRed line, demonstrating a high level of colocalization between DsRed and Orco in segments 9 and 10, but not in 11, where some DsRed-immunoreactive CSNs are spared (compare with Additional file 1: Figure S1a). *AN* antennal nerve, *CLSM* confocal laser-scanning microscopy, *CSN* chemosensory neuron, *mSTri* mechanosensilla trichoidea, *Orco* odorant receptor co-receptor, *SBas* sensilla basiconica, *SCoe* sensilla coeloconica, *Seg* segment, *SEM* scanning electron microscopy

To determine the distribution and number of CSNs, we used immunohistochemistry (IHC) with a cross-reactive antibody against Orco, fluorescent *in situ* hybridization with an *Orco*-specific probe, and a transgenic line, *EF1-B-DsRed*, that labels almost all and only CSNs in the adult antenna (see ‘Methods’ for a detailed characterization). Moreover, we generated an *Orco-Gal4* line that partially covers the Orco pattern, which we refer to as the partial Orco-Gal4 line (see ‘Methods’ for a detailed characterization of reagents). These different approaches unequivocally confirm that CSNs are restricted to the distal three segments (9–11) that form the enlarged club of the antenna [90] (Fig. 1a; Additional file 1: Figure S1a; and Additional file 2: Figure S2a). To improve on previous data in respect to the characterization, location, and exact number of antennal sensilla [90], we used in addition to the confocal laser-scanning microscopy (CLSM) approaches also scanning electron microscopy (SEM) (Figs. 1b–h and 2a–g). This morphologically verified the presence of chemosensory sensilla exclusively on the three club segments [90], with the highest number and diversity on the apical part of the terminal segment 11 (Fig. 1b–b''; Additional file 3: Figure S3).

Four mechanoreceptive and three chemoreceptive sensilla types could be confirmed by the combination of these techniques (Fig. 1b–b'') and the respective number of contained CSNs was identified. The mechanoreceptive sensilla include the spatulate bristles (SpaB; Fig. 1d–d''''), the mechanosensilla trichoidea (mSTri; Fig. 1e–e''''), the sensilla campaniformes (SCam; Fig. 1b''), and the sensilla chaetica (SCha; Fig. 1c–c''''), which are the most dominant sensilla type present on the lateral sites of all 11 segments (Fig. 1a). The chemoreceptive sensilla subdivide into chemo-sensilla trichoidea (cSTri, Fig. 1f–f'''), sensilla basiconica (SBas; Fig. 1g–g''''), and sensilla coeloconica (SCoe, Fig. 1h–h'''). For the chemoreceptive sensilla, segments 9 and 10 carry mostly SBas (about 15) arranged in an axial ring at the apical edge of each segment (Fig. 2f, g) and two SCoe (Fig. 2f, g'), whereas the terminal segment 11 harbors SBas (about 25), some SCoe (about 7), and many cSTri (about 87) (Fig. 1b–b''). A detailed analysis of the number and distribution of the different sensilla types in males and females revealed no sexual dimorphism (Additional file 3: Figure S3).

The number of CSNs per antenna was estimated based on the number of CSNs per sensillum or prong and the number of the respective sensilla per antenna. cSTri contain typically one Orco-immunoreactive OSN (Additional file 1: Figure S1b; Additional file 3: Figure S3i). This type of sensilla is known for its pheromone receiving abilities in Lepidoptera [91–93] and had been described as olfactory sensilla in *D. melanogaster* [59] and *Culex quinquefasciatus* [94]. SBas of *T. castaneum* consist of up to five prongs (Fig. 2a–e) like other Tenebrionidae [90, 95]. Each prong harbors about six CSNs (Additional file 3: Figure S3i) – the same number as in *Tenebrio molitor* [96]. Of them, four or five can be considered olfactory based on Orco-immunoreactivity (Additional file 1: Figure S1c). Findings in *Tribolium brevicornis* [95] suggest an additional gustatory function of SBas, leading to the conclusion that the SBas are bimodal chemosensilla. Because of this constant number of CSNs per prong and the shared lymphatic space (Fig. 1g–g''), we propose that multiple pronged SBas are derived from a fusion of single sensilla. Nonetheless, we refer to and count multiple pronged SBas as a single sensillum independent of the number of prongs. SCoe contain three CSNs (Additional file 3: Figure S3i) without Orco-immunoreactivity (Additional file 1: Figure S1d). The SCoe in *T. castaneum* might therefore harbor IRs as shown in *D. melanogaster* [19, 97]. Altogether, we found on each antenna about 100 prongs of SBas with six CSNs each, 87 cSTri with one CSN, and 11 SCoe with about three CSNs (Additional file 3: Figure S3). This leads to a total number of about 720 CSNs per antenna of *T. castaneum*.

### Anatomy of the olfactory pathway in the red flour beetle brain

#### Antennal projections

**Fig. 3:**
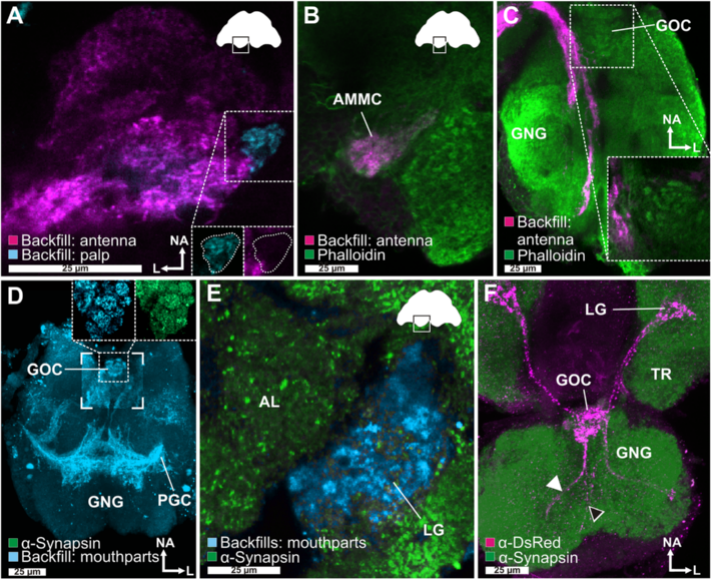
The central olfactory pathway of *T. castaneum*. **a** Backfill of one antenna (*magenta*) stains all glomeruli in the ipsilateral antennal lobe (*AL*) except one. This glomerulus is exclusively labeled by a backfill of a maxillary palp (*cyan*). **b** In addition to the *AL* glomeruli, backfilling (*magenta*) of one antenna labeled the ipsilateral antennal mechanosensory and motor center (*AMMC*), located n-dorsally to the *AL*, **c** as well as descending fibers to the gnathal ganglion (*GNG*). **d** Maximum intensity projection of the backfills of mouthparts (*cyan*) shows massive innervation of the *GNG* including the gnathal olfactory center (*GOC*) (magnified in the *inset*) and the primary gustatory center (*PGC*). **e** Backfill of the mouthparts (*cyan*) revealed in the cerebral ganglion beside innervation of a single ipsilateral *AL* glomerulus also projections in the ipsilateral lobus glomerulatus (*LG*). **f** Reporter expression of the partial *Orco*-Gal4/*UAS*-DsRed line (*magenta*) revealed two paired input tracts *(black* and *white arrowheads*) from the maxillary (*white arrowhead*) and labial palps (*black arrowhead*) that converge in a medial and n-anterodorsally located glomerular area, the *GOC*, and ascend to a microglomerularly organized area, the *LG*. See also Additional file 7: Movie S3. Orientation bars in (**a**) also apply for (**b**) and (**e**). *AL* antennal lobe, *AMMC* antennal mechanosensory and motor center, *GNG* gnathal ganglion, *GOC* gnathal olfactory center, *L* lateral, *LG* lobus glomerulatus, *NA* neuroaxis-anterior, *PGC* primary gustatory center, *TR* ﻿tritocerebrum﻿

To get an impression of the innervation pattern of chemosensory neuropils, we performed antennal and palpal backfills. Backfills via the antennal nerve labeled the ipsilateral AL (Fig. 3a; Additional file 4: Movie S1; Additional file 5: Movie S2; Additional file 6: Figure S4), the antennal mechanosensory and motor center (AMMC) (Fig. 3b), as well as a distinct area in the GNG (Fig. 3c). While this ipsilateral restriction is common in many insects [57], it is in contrast to *D. melanogaster* and *Ceratitis capitata*, where the majority of OSNs innervate the ipsi- and contralateral sides [59, 98]. The antennal backfills labeled all AL glomeruli except one, which is the only glomerulus labeled by backfills of the maxillary palp via the GNG (Fig. 3a; Additional file 5: Movie S2). This resembles the situation in Lepidoptera, where CO_2_ responsive CSNs from the palp project into a single AL glomerulus devoid of antennal innervation [99]. The descending antennal projections into the GNG (Fig. 3c) are not labeled in the partial *Orco*-Gal4/*UAS*-DsRed line and might therefore be from gustatory or mechanosensory neurons, as described in *Periplaneta americana* and *Locusta migratoria* [100, 101].

**Fig. 4:**
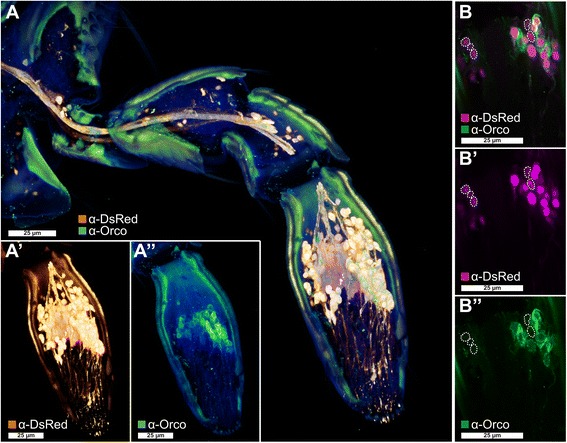
Orco-immunoreactive sensory neurons in the maxillary palp. **a** Voltex projection of a CLSM-stack showing antibody enhanced reporter expression of the *EF1*-*B*-DsRed line (**a**', *orange*) and Orco-immunoreactive cells (**a**'', *green*) in a halved maxillary palp. **b**–**b**'' Single optical section of (**a**) showing partial colocalization of Orco immunoreactivity and the reporter expression of the *EF1*-*B*-DsRed line (*magenta*). Dotted lines in (**b**) highlight reporter-expressing cells that are not Orco-immunoreactive. *CLSM* confocal laser-scanning microscopy, *Orco* odorant receptor co-receptor



**Additional file 4: Movie S1:** 3D reconstructions of the antennal nerve, antennal lobe, antennal mechanosensory and motor center, and lobus glomerulatus. Z-stack video of a phalloidin stained brain with embedded 3D reconstruction of antennal lobe (*dark blue*), antennal nerve (*light blue*), antennal mechanosensory and motor center (*turquoise*), and the lobus glomerulatus (*magenta*). Later the neuropils are embedded in a voltex projection of the brain, also based on phalloidin staining (*orange*). (MP4 11379 kb)




**Additional file 5: Movie S2:** Camera path through a confocal stack of the AL with backfills of the antenna and maxillary palp. Obtained from the same confocal stack as Fig. 3a. Antennal backfill in *green* and maxillary palp in *magenta*. (MP4 5185 kb)


#### Antennal lobe

For the AL of freshly eclosed adults, about 70 distinguishable olfactory glomeruli have been previously described using a synapsin antibody [102]. To evaluate the glomeruli number in ALs of beetles, 7 days after adult eclosion, we improved the analysis by deconvolution as well as using an additional antiserum against tachykinin-related peptides (TKRPs), which distinctly labels also densely packed glomeruli [103]. This more advanced analysis resulted in the 3D reconstruction of about 90 glomeruli per AL with no obvious sexual dimorphism (females: mean 89.2, standard deviation or SD = 4.9, *n* = 5; males: mean 89.4, SD, 7.6, *n* = 5).

#### Palpal projections into accessory olfactory centers

Whole mouthparts or maxillary palp backfills (Fig. 3d, e) revealed besides the already mentioned single AL glomerulus, innervation of three distinct neuropil areas: an unpaired glomerular organized neuropil in the GNG, the primary gustatory center also in the GNG [104], as well as an area near the AL, resembling the LG of hemimetabolous insects [57, 105]. The unpaired neuropil located n-anterodorsal in the GNG consists of 30 to 40 glomeruli (Fig. 3d, inset), which are all innervated from both sides of the mouthparts. This neuropil is also labeled by the partial *Orco*-Gal4/*UAS*-DsRed line (Fig. 3f; Additional file 7: Movie S3), which indicates innervation by OSNs originating in the maxillary or labial palps (Figs. 1a, 3f, 4a'', 4b''; Additional file 2: Figure S2f) that project via two tracts into the GNG. This neuropil, therefore, represents an olfactory processing center in the GNG that has to our knowledge never been described before and we term the gnathal olfactory center (GOC). Some of the fibers labeled by the palpal backfills, as well as the partial *Orco*-Gal4/*UAS*-DsRed line pass through the GOC, ascend via the neck connectives, and terminate ipsilaterally in an area medioventral to the AL (Fig. 3d), resembling the LG, which to date had only been described in hemimetabolous insects [57, 105]. Since the position, innervation, and glomerularly organized structure of this paired neuropil in *T. castaneum* is similar to the LG in cockroach, locust, and silverfish [57, 64–66], we refer to it as LG. In summary, our data suggest that in *T. castaneum*, odor information from the antennae and the mouthparts are processed separately. It appears that OSNs from the mouthparts do not project into the AL but into the GOC and the LG.



**Additional file 7: Movie S3:** Voltex projection of the gnathal ganglion and part of the brain of the *Orco*-Gal4/*UAS*-DsRed line. The video was obtained from the same confocal stack as Fig. 3f. It shows two paired input tracts from the maxillary and labial palps that converge in the *GOC* and ascend to the *LGs*, as well as the partially labeled *ALs*. (MP4 16700 kb)


#### Projection neurons

**Fig. 5:**
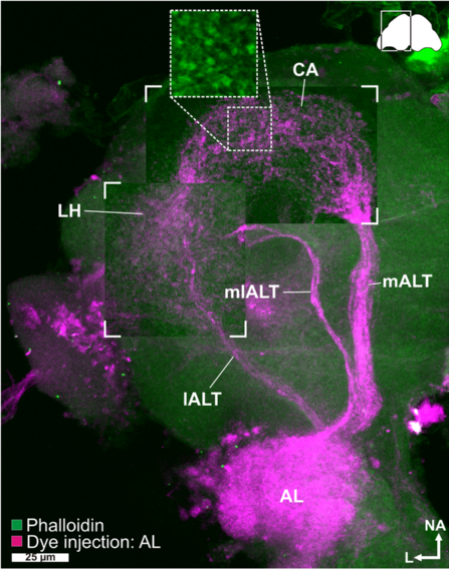
Antennal lobe tracts. Maximum intensity projection of a CLSM image stack after dye injection into the *AL* (*magenta*) revealed three antennal lobe tracts – the medial (*mALT*), mediolateral (*mlALT*), and the lateral antennal lobe tract (*lALT*) – as well as the calyx (*CA*) and the lateral horn (*LH*). In the *CA*, most fibers from the *mALT* form microglomeruli (*inset* obtained from another preparation). The staining in the optical lobe is an artifact caused by diffusion of the dye during application. Phalloidin counterstaining in *green. AL* antennal lobe, *ALT* antennal lobe tracts, *CA* calyx, *CLSM* confocal laser-scanning microscopy, *lALT* lateral antennal lobe tract, *LH* lateral horn, *mALT* mediolateral lobe tract, *mlALT* mediolateral lobe tract

Dye injections into the AL of adult *T. castaneum* revealed three ALTs formed by the PNs (Fig. 5), exclusively in the ipsilateral hemisphere. The most prominent tract, the medial antennal lobe tract (mALT), connects the AL with the calyx (CA) of the MB and the LH. The mediolateral antennal lobe tract (mlALT) passes the region near the MB spur without forming sub-branches (Fig. 5) and further projects to the LH. The lateral antennal lobe tract (lALT) projects directly to the most n-posterior part of the LH. We could not observe any obvious direct projections of the mlALT and the lALT to the CA. However, since they possibly overlap with trajectories of the mALT fibers from the CA to the LH [106], we cannot exclude their existence, as described for other holometabolous insects [70]. Previously only the mALT had been clearly identified in Coleoptera and the existence of a mlALT had only been presumed [70]. Our results indicate that three ALTs are a common feature among most holometabolous insects, including beetles.

#### Mushroom body

The detailed architecture of the MB of *T. castaneum* is described in [103]. The CA is innervated by the mALT (Fig. 5) and microglomerularly organized as indicated by phalloidin or synapsin antibody stainings (Fig. 5, inset). This is similar to several insects including *Apis mellifera* [107, 108] and *D. melanogaster* [109, 110] and suggests a comparable wiring with the PNs. The Kenyon cells (KCs) were identified in DAPI (4',6-diamidino-2-phenylindole) stainings based on their smaller and brighter stained nuclei [103]. The number of about 2700 KCs was determined by interpolation of volumetric data as well as by counting of the stained nuclei using MorphoGraphX [111]. Both procedures resulted in comparable numbers with the interpolation of 13 CAs from seven animals estimating about 2800 KCs (2795; SD: 214) and the counting of nine CAs from five specimen indicating approximately 2600 KCs (2613; SD: 204) per MB.

### Genome-wide expression analysis of genes involved in chemoreception in *T. castaneum*

**Fig. 6:**
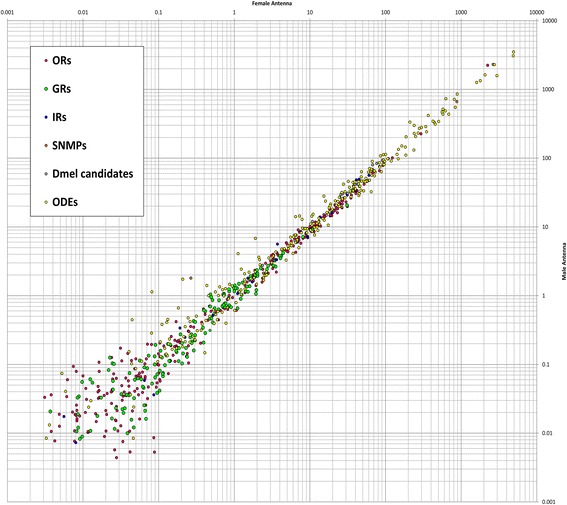
Comparison of expression levels in male and female antenna. Comparison of expression levels of odorant receptors (*ORs*, *magenta*), gustatory receptors (*GRs*, *green*), ionotropic glutamate-like receptors (*IRs*, *blue*), sensory neuron membrane proteins (*SNMPs*, *orange*), orthologous of candidates obtained from *D. melanogaster* (*Dmel candidates*, *grey*) and potential odorant degrading enzymes (*ODEs*, *yellow*) in male and female antennae. Average values based on two male and three female antennal samples. Scatter plot of the RPKM values. *Dmel D. melanogaster*, *GRs* gustatory receptors, *IRs* ionotropic glutamate-like receptors, *ODEs* odorant degrading enzymes, *ORs* odorant receptors, *RPKM* reads per kilobase per million, *SNMPs* sensory neuron membrane proteins

The fully sequenced genome of *T. castaneum* [81–83] led to the annotation of the major gene families involved in chemoreception. Based on genome data and computational gene predictions, the OBPs [112], CSPs [113], IRs [114], GRs [81], ORs [115], and SNMPs [116, 117] were annotated, but only for the ORs was a RT-PCR-based expression analysis performed [115]. To validate or correct the predicted gene models of these gene family members and to determine their tissue-specific expression, we performed transcriptome analyses of adult male and female antennae, heads (without antennae, but including mouthparts), mouthparts (the part of the head capsule, anterior to the antennal bases), legs, and bodies (without legs and head). In addition, we identified potential ODEs, as well as orthologs from further genes described to be involved in *D. melanogaster* olfaction. The detailed analysis of the OBPs and CSPs has already been published [89] and revealed that the majority of the classic OBPs and antenna binding proteins II (ABP II) seem to be involved in chemoreception while only a few of the minus-C OBPs (C-OBPs) and CSPs are enriched in antenna or mouthparts. The following results are based on this same set of transcriptome data (GEO accession number: GSE63162 (http://www.ncbi.nlm.nih.gov/geo/query/acc.cgi?acc=GSE63162 [118]). Like OBPs and CSPs [89], also for the genes presented here, no significant differences on the expression level between male and female antenna samples were identified (Fig. 6). Therefore, the female and male antenna samples can serve as biological replicates and indicate that reads above 0.1 reads per kilobase per million (RPKM) are reproducible (Fig. 6). However, to minimize the rate of potential false positives in our description, we considered only genes with RPKM ≥ 0.5 as expressed. We are aware that this might lead to an underestimation of the expressed gene numbers for each class of genes. Since it is impossible to determine the exact number of genes that are functionally involved in chemoreception based only on transcriptomic expression analyses, we always present two values for expressed genes, one based on RPKM ≥ 0.5 and the other defined by statistical analysis as significantly enriched over body. All raw values and the re-annotated gene models are summarized in Additional file 8.

#### Tissue-specific expression of ionotropic glutamate-like receptors

**Fig. 7:**
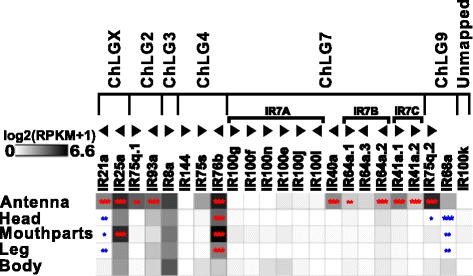
Expression of *T. castaneum* ionotropic glutamate-like receptors (*IRs*). Heat map showing the expression level of the 23 *IRs* as a log_2_[RPKM + 1] value in different tissues [adult antennae, head (missing antennae but including mouthparts), mouthparts, legs, and body]. The candidates are ordered according to their chromosomal localization (Additional file 9: Figure S5b). *Horizontal brackets* above indicate clustering in the genome. The *arrowheads* represent the orientation of the open reading frame. The expression levels are represented by a *greyscale* with highest shown expression levels labeled *black*. The *asterisks* mark statistically significantly differentially expressed genes compared to body (based on biological replicates of five antennal, two head, three mouthpart, two leg, and two body samples). The *red asterisks* represent up- and the *blue* down-regulation (*p* values adjusted are * < 0.05, ** < 0.01, and *** < 0.001). *IR* ionotropic glutamate-like receptor, *RPKM* reads per kilobase per million

The RNAseq based revision of the 23 previously annotated IRs [114] confirmed the sequences of three open reading frames (ORFs); 17 had to be modified, two were incompletely covered by reads, and for a single one, no expression was detected (color coded in Additional file 8: Table S1, column B). In antennae, 16 of the IRs were significantly enriched compared to body (Fig. 7; Additional file 9: Figure S5a). In the mouthparts, five IRs are expressed, two are significantly enriched.

**Fig. 8:**
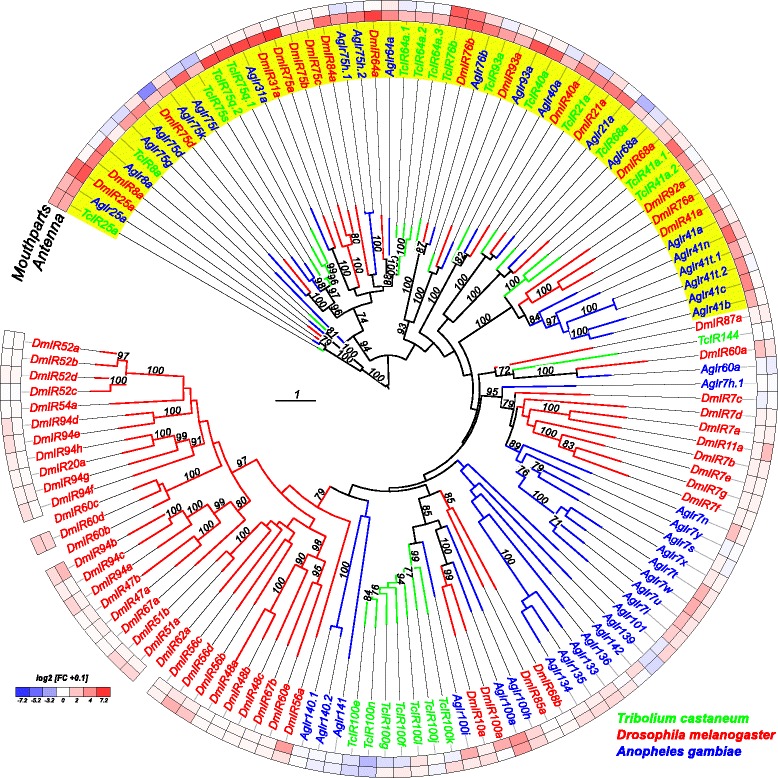
Phylogenetic tree of IRs. Based on protein sequences from *T. castaneum* (*green branches*), *D. melanogaster* (*red branches*), and *An. gambiae* (*blue branches*). The tree was rooted using the IR8/IR25 clade, according to [114]. Robustness of the tree topology was evaluated by 100 rapid bootstrap replications. *Outer rings* represent the expression in antennae and mouthparts (*T. castaneum*: palps, mandible, labrum, and labium; *D. melanogaster*: palp and proboscis; *An. gambiae*: maxillary palp) as log_2_-fold change compared to body corresponding to the scale in the *left lower corner*. The *scale bars* within the trees represent one amino acid substitution per site. Antennal IRs are highlighted in *yellow*. Basically the same figure is available with absolute values instead of fold changes to get an impression of the tissue-specific abundance of the transcripts as Additional file 10: Figure S6. *IR* ionotropic glutamate-like receptor

Comparing expression profiles of the IRs from *T. castaneum*, *D. melanogaster*, and *Anopheles gambiae* confirmed the antennal specific expression, as well as the high degree of phylogenetic conservation of the antennal IRs (Fig. 8; Additional file 10: Figure S6; highlighted in yellow) as proposed [114]. In contrast, the divergent IRs are non-antennal specifically expressed and are highly radiated within species clades as previously shown or predicted [114]. *T. castaneum* has a lower number of IRs compared to *D. melanogaster* and *An. gambiae*, due to lesser expansions of divergent IRs, but maintains the basic repertoire of antennal IRs (Fig. 8; highlighted in yellow). The homologs of IR25a, IR93a, and IR40a, which are necessary for humidity perception in *D. melanogaster* [119], are significantly enriched in antennae. IR40a is exclusively expressed in antennae, which correlates with the essential role of antennae in *T. castaneum* hygro-perception [90]. The homolog of the highly sensitive salt receptor and possible co-receptor IR76b [20, 120] is significantly enriched in antennae, mouthparts, and legs, while the co-receptors IR8a and IR25a [20] are highly expressed in all tissues of *T. castaneum* (Fig. 7).

#### Tissue-specific expression of gustatory receptors

**Fig. 9:**
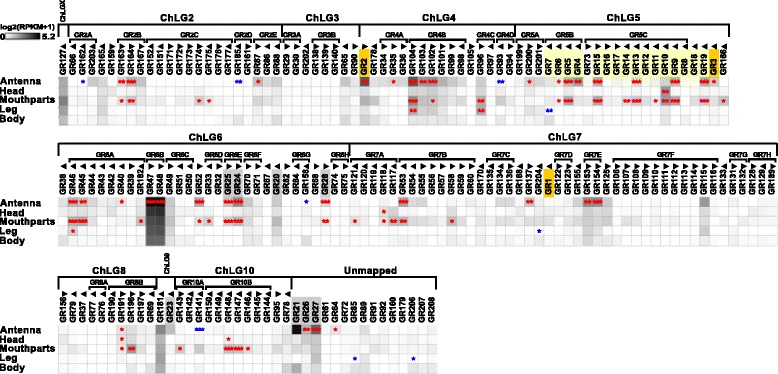
Expression of *T. castaneum* gustatory receptors (*GRs*). Heat map showing the expression level of the 207 analyzed *GRs* as a log_2_[RPKM + 1] value in different tissues [adult antennae, head (missing antennae but including mouthparts), mouthparts, legs, and body]. The candidates are ordered according to their chromosomal localization (Additional file 11: Figure S7b). *Horizontal brackets* above indicate clustering in the genome. The *arrowheads* represent the orientation of the open reading frame. The expression levels are represented by a *greyscale* with the highest shown expression levels labeled *black*. The *asterisks* mark statistically significantly differentially expressed genes compared to body (based on biological replicates of five antennal, two head, three mouthpart, two leg, and two body samples). The *red asterisks* represent up- and the *blue* down-regulation (*p* values adjusted are * < 0.05, ** < 0.01, and *** < 0.001). CO_2_ receptors are highlighted in *orange*, fructose receptor related genes in *grey*, and sugar receptors in *yellow. GR* gustatory receptor, *RPKM* reads per kilobase per million,

Of the 220 previously annotated GRs [81], only 207 genes had available gene models [82, 83]. Our transcriptome analysis verified the ORFs of 58 GRs, showed slight differences for 20 GRs, but did not or only incompletely cover 129 GRs (Additional file 8: Table S1, column B). In the antennae, 62 GRs are expressed, with 34 being significantly enriched and 10 being antennal-specific. Of the 69 mouthpart-expressed GRs, 36 are significantly enriched and 19 exclusive. Seventeen GRs are significantly enriched in both antenna and mouthparts. In legs, 18 GRs are expressed with three being significantly enriched (Fig. 9; Additional file 11: Figure S7a).

**Fig. 10:**
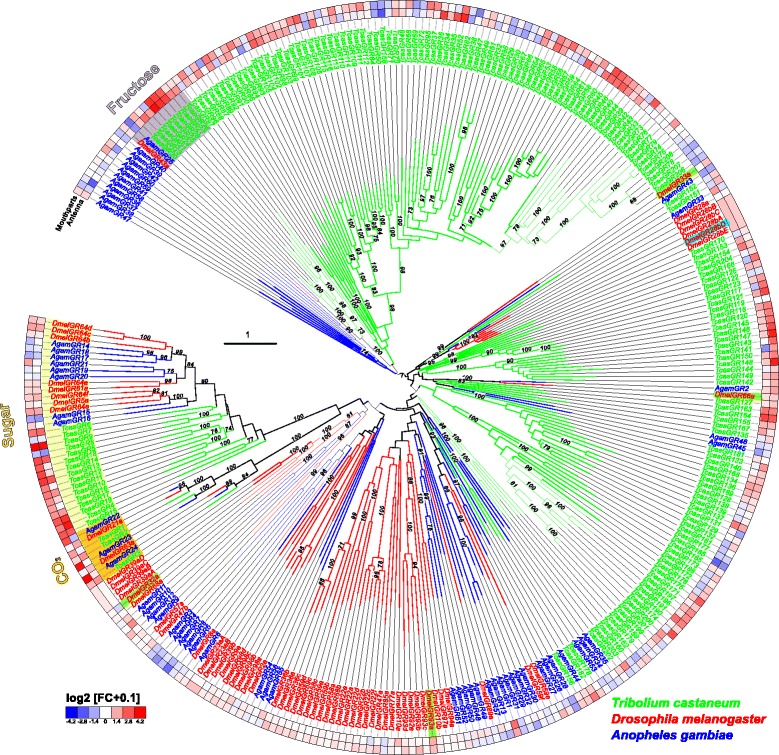
Phylogenetic tree of gustatory receptors (*GRs*). Mid-point rooted tree based on protein sequences from *T. castaneum* (*green branches*), *D. melanogaster* (*red branches*), and *An. gambiae* (*blue branches*). Robustness of the tree topology was evaluated by 100 rapid bootstrap replications. *Outer rings* represent the expression in antennae and mouthparts (*T. castaneum*: palps, mandible, labrum, and labium; *D. melanogaster*: palp and proboscis; *An. gambiae*: maxillary palp) as log_2_-fold change compared to body corresponding to the scale in the *left lower corner*. The *scale bars* within the trees represent one amino acid substitution per site. Potential sugar receptors (highlighted in *yellow*), fructose receptors (highlighted in *grey*), and CO_2_ receptors (highlighted in *orange*) are labeled. Known bitter receptors from *D. melanogaster* are highlighted in *green*, and the thermos-sensitive GR28bD in li*ght blue*. Basically the same figure is available with absolute values instead of fold changes to get an impression of the tissue-specific abundance of the transcripts as Additional file 12: Figure S8. *GR* gustatory receptor

The phylogenetic comparison of the GRs in *T. castaneum*, *D. melanogaster*, and *An. gambiae* (Fig. 10; Additional file 12: Figure S8) confirmed that only the CO_2_ receptors (highlighted in orange) are highly conserved [121]. The other GRs seem to have undergone independent radiation during the transition to *T. castaneum*: e.g. the sugar receptor-related branch (highlighted in light yellow) contains 16 genes [122], twice the number compared to the two chosen dipterans. In addition, the single fructose receptor (highlighted in grey) found in *D. melanogaster* and *An. gambiae* is represented by eight homologs in *T. castaneum*. The remaining 180 GRs belong to several *T. castaneum*-specific expansion groups. Specific orthologs to the known bitter receptors of *D. melanogaster* [13] as well as to the thermo-sensitive *Dmel*GR28bD [123] cannot be predicted based on our phylogenetic analysis.

Like other insects [121, 124, 125], *T. castaneum* has three CO_2_ receptors (*Tcas*GR1, *Tcas*GR2, and *Tcas*GR3), while *D. melanogaster* has only two that form functional heteromers [126, 127]. In *T. castaneum*, the expression of the CO_2_ receptors is not restricted to one of the chemosensory organs with *Tcas*GR2 and *Tcas*GR3 being significantly enriched in antennae but also being expressed together with *Tcas*GR1 in the mouthparts (Fig. 9; highlighted in orange). This dual input is in contrast to but combines both the expression of the three *An. gambiae* CO_2_ receptors that are restricted to the maxillary palps [128, 129], as well as the two *D. melanogaster* CO_2_ receptors that are mainly expressed in the antennae [126, 127, 130].

The presence of GRs on insect antenna had previously been postulated based on physiological response to sugars [95, 131–133] and was identified by antennal expression analysis [124, 127, 128, 134–136]. Our interspecies comparison (Fig. 10) confirms the antennal enrichment of several GRs in the two analyzed dipterans. However, the high number of 34 significantly enriched GRs in the antenna of *T. castaneum* is unusual, but reflects the increased total number of GRs in this species. Interestingly, the GRs of *T. castaneum* are present in both antenna and mouthparts at similar numbers and expression levels (Fig. 9; Additional file 11: Figure S7a).

#### Tissue-specific expression of odorant receptors

**Fig. 11:**
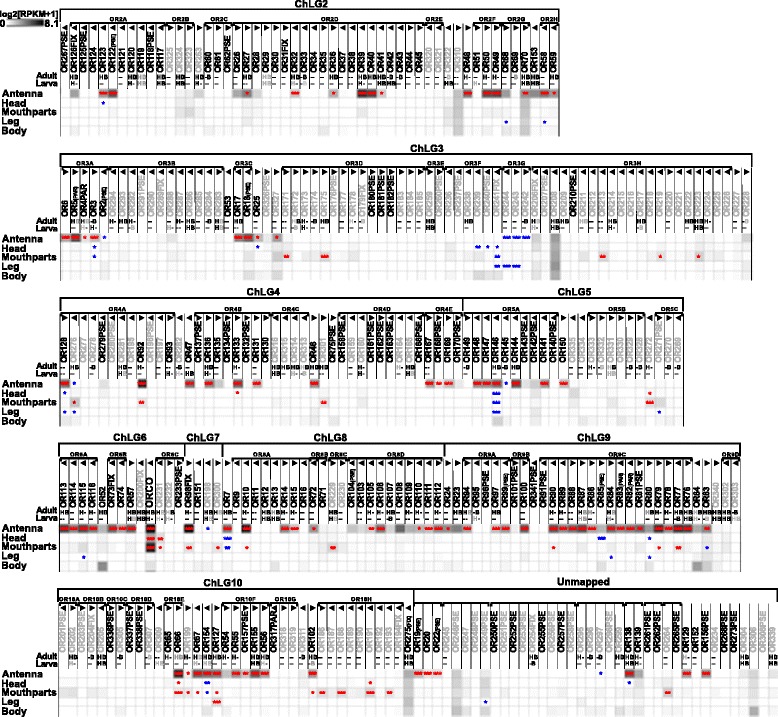
Expression of *T. castaneum* odorant receptors (ORs). Heat map showing the expression levels of the 337 analyzed ORs as log_2_[RPKM + 1] with a maximum of 8.1 (Orco has a value of 11.1 in antenna) in different tissues [adult antennae, head (missing antennae but including mouthparts), mouthparts, legs, and body]. The candidates are ordered according to their chromosomal localization (Additional file 13: Figure S9c). *Horizontal brackets* above indicate clustering in the genome, and the *arrowheads* represent the orientation of the open reading frame. ORs that are member of clades four, five, and six [115] are written in *grey letters*. The line labeled with *Adult* and *Larva* refers to data from [115]. The character *H* (respectively *B*) indicates that the corresponding *OR* was detected in head or body cDNA samples by reverse PCR of the labeled developmental stage. A *black letter* indicates that an amplicon was detected in the majority of replicates, a *grey letter* means only in a few replicates, a *dash* indicates no PCR product and no character means no data available. A comparison of the number of expressed genes is summarized in Additional file 13: Figure S9b. The expression levels are represented by a *greyscale* with highest shown expression levels (3 RPKM or higher) labeled *black* to make sure that also low level expression is identifiably presented. The *asterisks* mark statistically significantly differentially expressed genes compared to body (based on biological replicates of five antennal, two head, three mouthpart, two leg, and two body samples). The *red asterisks* represent up- and the *blue* down-regulation (*p* values adjusted are * < 0.05, ** < 0.01, and *** < 0.001). *B* body, *H* head, *OR* odorant receptor, *RPKM* reads per kilobase per million

Of the 341 previously annotated OR sequences [115], we could re-analyze 337 based on our RNAseq data. This revision confirmed 97, and 22 were re-annotated reviving eight previously indicated pseudogenes [115], namely *Tcas*OR2, *Tcas*OR18, *Tcas*OR19, *Tcas*OR22, *Tcas*OR85, *Tcas*OR99, *Tcas*OR104, and *Tcas*OR122. Moreover, 219 genes were not or only partially covered by our transcriptome data (color coded in Additional file 8: Table S1, column B). Over all samples, 170 ORs are expressed (Fig. 11; Additional file 13: Figure S9a). In antennae, 129 ORs are expressed, with 92 being significantly enriched and 99 exclusive. In the mouthparts, 49 ORs are expressed, with 28 being significantly enriched and 27 exclusive. In addition, 16 of the significantly mouthpart-enriched ORs are not enriched in the antenna (Fig. 11). The expression of typical ORs in the mouthparts is consistent with the high expression of Orco in this tissue (Figs. 1a and 4) and with observations in other insect species [128, 129, 137, 138]. In legs, ten ORs are expressed (Additional file 13: Figure S9a) but only one, namely *Tcas*OR127, is statistically enriched (Fig. 9).

**Fig. 12:**
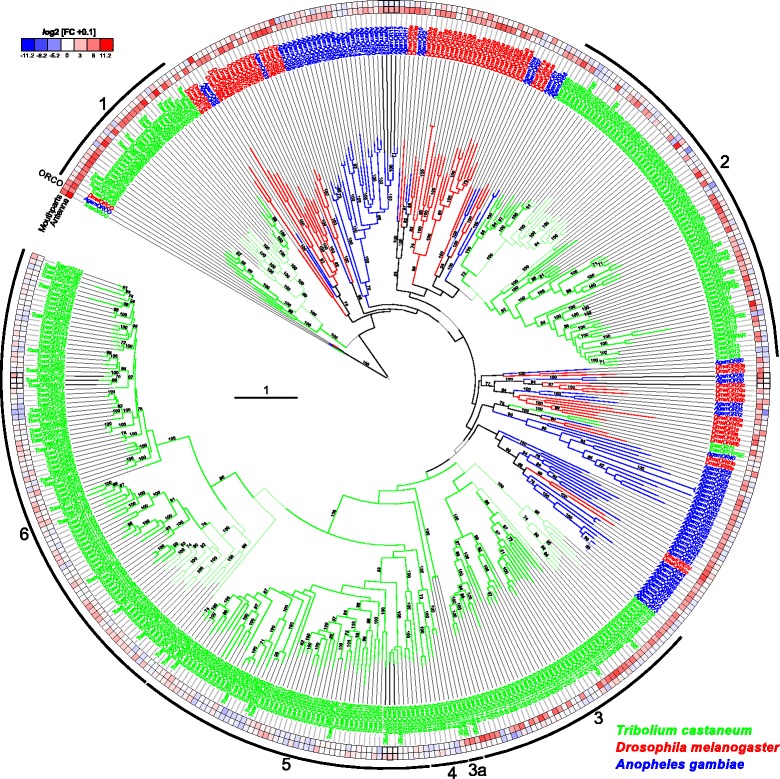
Phylogenetic tree of odorant receptors (ORs). Protein sequences (>300 amino acids) from *T. castaneum* (*green branches*), *D. melanogaster* (*red branches*), and *An. gambiae* (*blue branches*). The tree was rooted using the Orco clade, according to [24]. Robustness of the tree topology was evaluated by 100 rapid bootstrap replications. *Outer rings* represent the expression in antennae and mouthparts (*T. castaneum*: palps, mandible, labrum, and labium; *D. melanogaster*: palp and proboscis; *An. gambiae*: maxillary palp) as log_2_-fold change compared to body corresponding to the scale in the *left upper corner*. The surrounding numbers on the *outer thin line* indicate the expansion groups 1 to 6 [115]. *TcasOR71* and *TcasOR72PSE* were previously assigned to expansion group 1. The *scale bar* within the tree represents one amino acid substitution per site. Basically the same figure is available with absolute values instead of fold changes to get an impression of the tissue-specific abundance of the transcripts as Additional file 14: Figure S10. *OR* odorant receptor

The phylogenetic comparison of OR expression patterns in *T. castaneum*, *D. melanogaster*, and *An. gambiae* (Fig. 12; Additional file 14: Figure S10) revealed that the atypical odorant co-receptor Orco (in *T. castaneum* previously called *Tc*OR1 [115]) is the highest expressed OR in all tissues of all three species. In *T. castaneum*, Orco is expressed highest in antenna, followed by mouthparts. Orco is the only OR of *T. castaneum* with clear orthologs in dipterans [115, 139]. The high expression levels, the distribution, and the evolutionary conservation of Orco are consistent with its ancestral origin [24] and its outstanding role as a chaperone and co-receptor, forming functional heteromers with all typical ORs [140, 141].

The exceptional high number of typical ORs (Fig. 12) in *T. castaneum* is the result of large gene radiations within the coleopteran and tenebrionid lineages [136], which were previously subdivided into six expansion groups (Fig. 12) [115]. Expansion groups 1, 2, and 3 are conserved in other coleopterans [136] and are mainly expressed in antennae. The ORs of the expansion groups 4, 5, and 6 are highly derived, have no described homologs in other insects, and their expression is unusually often mouthpart-enriched (Fig. 11; grey lettering). This is consistent with the elaborated role of the mouthparts in *T. castaneum* olfaction. Specific orthologs to deorphanized ORs of *D. melanogaster* [142] cannot be predicted based on our phylogenetic analysis.

#### Identification and expression of potential odorant degrading enzymes

**Fig. 13:**
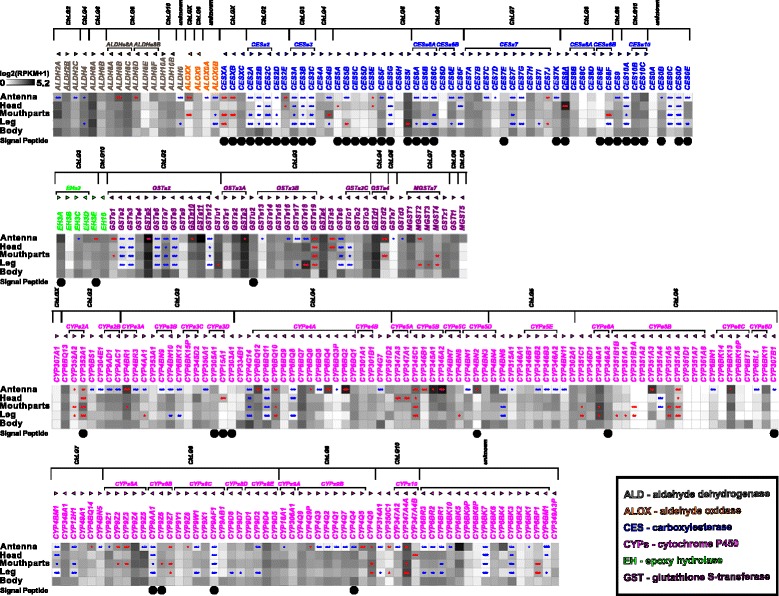
Expression of *T. castaneum* potential odorant degrading enzymes (ODEs). Heat map showing the expression level of the 263 potential ODEs as a log_2_[RPKM + 1] value in different tissues [adult antennae, head (missing antennae but including mouthparts), mouthparts, legs, and body]. The candidates are ordered according to their protein family and chromosomal localization. *Horizontal brackets* above indicate clustering in the genome (Additional file 15: Figure S11), and the *arrowheads* represent the orientation of the open reading frame. Underlined genes were previously found on the protein level in antennae by [89]. The expression levels are represented by a *greyscale* with the highest shown expression levels labeled *black*. The *asterisks* mark statistically significantly differentially expressed genes compared to body (based on biological replicates of five antennal, two head, three mouthpart, two leg, and two body samples). The *red asterisks* represent up- and the *blue* down-regulation (*p* values adjusted are * < 0.05, ** < 0.01, and *** < 0.001). A *black dot* in the lowest line indicates a predicted signal peptide according to a SignalP 4.0 [211] prediction. *ODE* odorant degrading enzyme, *RPKM* reads per kilobase per million

The genome of *T. castaneum* contains 15 aldehyde dehydrogenases (ALDHs) (Fig. 13 and Additional file 15) with two of them being significantly enriched, but not exclusively expressed in antenna. We found four predicted genes encoding aldehyde oxidases (ALOXs) with one being highly enriched in antennae and mouthparts, which, in contrast to ALOX ODEs from lepidopterans [143–145], does not encode a signal peptide (Fig. 13). Five of the 54 identified carboxylesterases (CESs) are significantly enriched in antenna, with two of them also in the mouthparts. Two other CESs are significantly enriched exclusively in the mouthparts. Five of these seven candidates show a predicted signal peptide for secretion (Fig. 13). *Tcas*CESXA shares sequence similarities with *D. melanogaster* Est6, and *Tcas*CES7J with *Dmel*JHEdup, with both *D. melanogaster* homologs having previously been identified as ODE candidates [54, 55]. *TcasCES10C* is expressed highest in antennae and related to a pheromone degrading enzyme from the Japanese beetle, *Popillia japonica* [146]. We identified six epoxide hydrolases (EHs), which are supposed to be membrane bound ODEs [147], with one being significantly enriched in antennae and having a predicted signal peptide (Fig. 13). The glutathione S-transferases (GSTs) of *T. castaneum* had already been annotated [148]. The revision confirmed most gene models, only *TcasGSTd2* and *TcasMGST2* had to be modified (available in Additional file 8: Table S1). Eight of the 41 GSTs are significantly enriched in antennae, with three also in the mouthparts (Fig. 13). One of these three, *TcasGSTd2* represents a member of the GST delta subfamily such as GST-msolf1 from *Manduca sexta*, which is an olfactory-specific GST expressed specifically in the sex-pheromone-detecting sensilla [149]. Analysis of the 141 previously described cytochrome P450s (CYPs) [150] revealed that two predicted gene models (*CYP347A4* and *CYP351B1*) were fusions of two separate genes (now termed *CYP347A4A* and *CYP347A4B*, as well as *CYP351B1A* and *CYP351B1B*, respectively). Seven other predictions had to be adjusted based on RNAseq data (sequences available in Additional file 8: Table S1). The expression analysis of these 141 genes showed that 26 are significantly enriched in the antenna, with 11 also in the mouthparts (Fig. 13). In addition, six CYPs are significantly enriched in mouthparts, but not in antennae. For the coleopteran *Phyllopertha diversa*, CYPs have been shown to be involved in pheromone degradation in a membrane-bound manner [51].

#### Expression of potential olfaction signal transduction pathway components

**Fig. 14:**
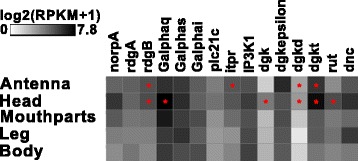
Expression of *T. castaneum* homologs of genes described to be involved in olfaction of *D. melanogaster*. Heat map showing the expression level of the several genes supposed to be involved in *D. melanogaster* olfaction, as a log_2_[RPKM + 1] value in different tissues [adult antennae, head (missing antennae but including mouthparts), mouthparts, legs, and body]. The expression levels are represented by a *greyscale* with highest shown expression levels labeled *black*. The *asterisks* mark statistically significantly differentially expressed genes compared to body (﻿based on biological replicates of five antennal, two head, three mouthpart, two leg, and two body samples). The *red asterisks* represent up-regulation (*p* values adjusted are * < 0.05). *RPKM* reads per kilobase per million

The orthologs of genes encoding signal transduction pathway components known to be involved in olfaction of *D. melanogaster* [46] were identified by BLAST and manually curated. The expression analysis revealed that four of them (*rdgB*, *itpr*, *dgkd*, and *dgkt*) are significantly enriched in the antennae (Fig. 14). However, there is no chemosensory-specific candidate exclusively expressed in antennae or mouthparts. Our data, therefore, do not indicate a chemosensory-specific metabotropic signal transduction pathway.

#### Expression and distribution of sensory neuron membrane proteins

**Fig. 15:**
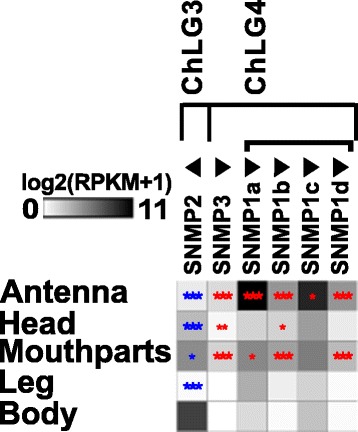
Expression of *T. castaneum* sensory neuron membrane proteins (*SNMPs*). Heat map showing the expression level of the six sensory neuron membrane proteins of *T. castaneum*, as a log_2_[RPKM + 1] value in different tissues [adult antennae, head (missing antennae but including mouthparts), mouthparts, legs, body, as well as larval head and body]. The candidates are ordered according to their chromosomal localization (Additional file 9: Figure S5b). *Horizontal brackets* above indicate clustering in the genome, and the *arrowheads* represent the orientation of the open reading frame. The expression levels are represented by a *greyscale* with highest shown expression levels labeled *black*. The *asterisks* mark statistically significantly differentially expressed genes compared to body (based on biological replicates of five antennal, two head, three mouthpart, two leg, and two body samples). The *red asterisks* represent up- and the *blue* down-regulation (*p* values adjusted are * < 0.05, ** < 0.01, and *** < 0.001). *SNMP* sensory neuron membrane protein, *RPKM* reads per kilobase per million

The transcriptome analysis revealed that one of the seven previously identified *Tcas*SNMPs [116, 117], namely XP_969729 [116], was incorrectly annotated and does not encode for a CD36-related protein. Moreover, the gene model previously named SNMP1c (XM_001816389) was a fusion of two SNMPs and overlaps with SNMP1d (XM_001816391) [117]. In our re-annotation, we removed XP_969729 and separated *Tcas*SNMP1c and *Tcas*SNMP1d. In addition, the gene models of *Tcas*SNMP2, *Tcas*SNMP1a, and XP_975606 [116] had to be modified based on transcriptome and RACE-PCR data. For XP_975606, we propose the name *Tcas*SNMP3, to reflect its unclear phylogenic relationship. Despite the more SNMP1-like expression pattern (Fig. 15) and chromosomal localization (Additional file 9: Figure S5b) of *Tcas*SNMP3, the comparison of the amino acid composition revealed no clear affiliation to either the SNMP1 or the SNMP2 subgroup [151]. Interspecies comparison revealed no clear orthology of *Tcas*SNMP3 to SNMPs from other species, including the so-called SNMP3 of *Calliphora stygia* [152], which, based on phylogeny, clearly represents an SNMP1 homolog. All six *Tcas*SNMPs are expressed in antennae (Fig. 15), which was also confirmed by rapid amplification of cDNA-ends PCR ﻿(RACE-PCR) based on an antennae cDNA pool, but only *Tcas*SNMP1a-d and *Tcas*SNMP3 are significantly enriched in antennal tissue. Moreover, three of the *Tcas*SNMP1, as well as *Tcas*SNMP3, are also enriched in mouthparts (Fig. 15), further supporting the importance of the mouthparts for olfaction in *T. castaneum*. In contrast, *Tcas*SNMP2 is expressed highest in body and significantly underrepresented in antennae and mouthparts (Fig. 15), which is similar to its ortholog in *D. melanogaster* [49]. Despite the observation that in most insects with a fully sequenced genome only two SNMPs were found [116, 117], the relatively high amount of six *Tcas*SNMPs of *T. castaneum* is not unique, since transcriptome analysis, e.g., of other beetles, revealed four SNMPs in *Dendroctonus valens* [153] and *Dastarcus helophoroides* [154], as well as three in *Ips typographus* and *Dendroctonus ponderosae* [136]. However, *T. castaneum* is currently only exceeded by the hessian fly (*Mayetiola destructor*) with seven expressed SNMPs [155].

## Discussion

### Independent integration centers for antennal and palpal olfactory perception

**Fig. 16:**
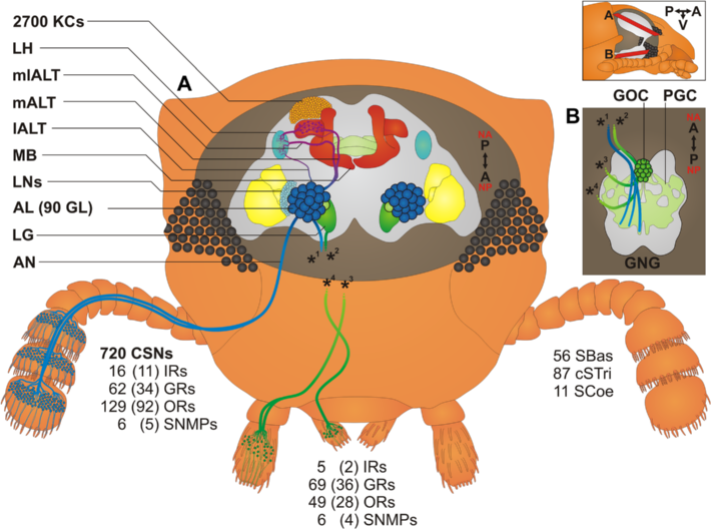
*T. castaneum* head scheme depicting the major olfactory pathway components. **a** Head section (dorsal view) showing the brain and the *CSNs* from the antenna (*blue*) and the mouthparts (*green*). **b** Head section (ventral view) showing the *GNG*. Section orientation is indicated at the *upper right corner* (lateral view of the head; *﻿V, ventral*). *Double-headed arrows* indicate body (*A*, anterior ↔ *P*, posterior; *black*) and neuro-axis (*NA*, n-anterior ↔ *NP*, n-posterior; *red*). Chemical signals are sensed by about 720 *CSNs* located in 56 *SBas*, 87 *cSTri*, and 11 *SCoe* on the last three antennal segments. These *CSNs* express 16 *IRs*, 62 *GRs*, 129 *ORs*, and six *SNMPs*. Chemosensory information is also perceived in the palps by five *IRs*, 69 *GRs*, 49 *ORs*, and six *SMNPs* (number in brackets indicates significantly enriched members compared to body). The antennal nerve (*AN*) projects into the ipsilateral *AL*, where all except one (*light green*) of the about 90 *GL* (*dark blue*) are innervated. A separate antennal tract (*^1^) descends into the *GNG* (**b**, *blue*), where presumably gustatory and mechanosensory information is processed. Incoming olfactory information is processed by a complex network of local interneurons (*LNs*) in the *AL* and further relayed by projection neurons forming three *ALTs*. The medial *ALT* (*mALT*) projects to and arborizes in the calyx of the *MB* formed by about 2700 *KCs* (*orange*) to eventually innervate the *LH* (*light blue*). The mediolateral ALT (*mlALT*) and lateral *ALT* (*lALT*) directly innervate the *LH*. From the mouthparts, *CSNs* project via the maxillary (*^3^) and labial palp nerves (*^4^) into the *GNG*, where the gustatory information is processed in the *PGC*. The olfactory sensory input from the palps is processed in an unpaired glomerularly organized *GNG* structure, the *GOC*, as well as in the *LG*, which receives input from some palpal OSNs via ascending neurons (*^2^) passing through the *GOC*. Some of the palp-derived chemosensory information is processed in the single *AL* glomerulus, which lacks antennal innervation and is, therefore, exclusively innervated by projections from the mouthparts (*light green*). *AL* antennal lobe, *ALT* antennal lobe tracts, *AN* antennal nerve, *CSN* chemosensory neuron, *cSTri* chemosensilla trichoidea, *GL* antennal lobe glomeruli, *GNG* gnathal ganglia, *GOC* gnathal olfactory center, *GR* gustatory receptor, *IR* ionotropic glutamate-like receptor, *KC* Kenyon cells, *lALT* lateral antennal lobe tract, *LG* lobus glomerulatus, *LH* lateral horn, *LNs* local interneurons, *mALT* mediolateral lobe tract, *MB* mushroom body, *mlALT* mediolateral lobe tract, *OR* odorant receptor, *OSN*, olfactory sensory neuron; *PGC* primary gustatory center, *SBas* sensilla basiconica, *SCoe* sensilla coeloconica, *SNMP* sensory neuron membrane protein

In *T. castaneum*, odorants are mainly perceived with the last three segments of the antenna, which carries three types of chemoreceptive sensilla (SBas, cSTri, and SCoe), as well as with the maxillary and labial palps (Fig. 16). Accordingly, expression analysis revealed that ORs are mostly expressed in antennae, but also in the mouthparts (Figs. 11 and 12; Additional file 13: Figure S9a) as previously shown for several dipteran species [128, 129, 137, 156–158]. In contrast to the Diptera, where the palps are chemosensory appendages with limited odor coding complexity, the relatively high number of Orco-immunoreactive CSNs (Fig. 4) as well as the high number of expressed ORs, SNMPs, potential ODEs, and OBPs [89] in *T. castaneum* mouthparts (Figs. 11, 13, 15, and 16) imply a more prominent role of the palps in olfaction. The palpal ORs are possibly involved in the evaluation of the quality of food sources, like the ORs on the proboscis of *Manduca sexta* [159].

Moreover, in addition to the differences on the perception level, major dissimilarities to the Diptera occur on the level of odor processing. The data from the partial *Orco*-Gal4 line as well as the backfills from the antenna and the mouthparts indicate that processing olfactory information at least at the level of the first central relay station occurs independently of each other (Fig. 16). This is surprising, as many of the ORs expressed on the mouthparts are also expressed on the antennae. In contrast, typical OR expression is mutually exclusive between antenna and palps in *D. melanogaster* and *An. gambiae* [128, 137], where in addition, projections from the palps innervate several AL glomeruli [63, 160, 161]. In *T. castaneum*, the olfactory input stemming from the antenna seems to be processed exclusively in the AL (Fig. 3a; Additional file 5: Movie S2; Additional file 6: Figure S4), whereas the palpal-derived olfactory information is essentially processed outside the AL, in the LG (Fig. 3e, f; Additional file 5: Movie S2) and the GOC, an unpaired and glomerularly organized first olfactory center in the GNG (Fig. 3d, f; Additional file 7: Movie S3). The LG had, as far as we know, previously been described only in hemimetabolous insects [57, 64–66, 105]. A glomerularly organized olfactory center in the GNG such as the GOC has, to our knowledge, not been described in any insect so far. The number of 49 ORs (with 28 being significantly enriched compared to body) that are expressed in the mouthparts is roughly consistent with the estimated 30 to 40 glomeruli in the GOC. This suggests that the wiring in the GOC may resemble the situation in the ALs with the difference being convergence into an unpaired medial structure. The only palpal projection into the AL is a mutually exclusive innervation of a single ipsilateral glomerulus (Fig. 3a; Additional file 5), which may be involved in CO_2_ perception, as described in several moth species [99] and proposed for some mosquitoes [161, 162].

### Antennae serve also as key organs for gustatory perception

In *T. castaneum*, antennae and mouthparts express similar high numbers and levels of GRs, which indicates the antenna as a key gustatory organ besides the mouthparts (Fig. 9; Additional file 11: Figure S7a). This finding may reflect the beetles’ ground-dwelling life style and indicates that the scanning behavior with the antennae not only gathers tactile but also chemical stimuli. This is in contrast to the Diptera, where the labellum is the main gustatory organ [163–165].

### Postulation of exceptions to the central dogma

The number of 129 ORs that we found to be expressed in *T. castaneum* antennae (Fig. 11; Additional file 13: Figure S9a) exceed the numbere of about 90 glomeruli in the AL. Moreover, some glomeruli are likely to get exclusive innervation by OSNs that express IRs, as described in *D. melanogaster* [20]. These observations do not conciliate with the central dogma postulating that OSNs express only one typical OR and all OSNs carrying this same OR converge into one and the same glomerulus, which was hypothesized to be the typical situation for insects [61, 137, 166]. However, for *D. melanogaster*, both co-expression of more than one typical OR per OSN as well as co-convergence due to innervation of one AL glomerulus by more than one OSN sub-type have been already described as exceptions [137, 167]. For *T. castaneum*, we propose that such exceptions are much more frequent.

### Large repertoire of potentially functional odorant receptor genes and possible environmental regulation

The genome of *T. castaneum* harbors 341 OR genes [81, 115], of which 270 seem to encode for functional ORs. Of the 337 ORs with available full sequence information [115], we find in our RNAseq data 161 ORs to be expressed in adult antennae, mouthparts, and head by a threshold of 0.5 RPKM (Additional file 13: Figure S9a). In comparison to the RT-PCR-based data from [115], who found 112 ORs to be clearly expressed in adult heads, we only confirmed 82 ORs. In addition, we identified 41 ORs previously declared as not expressed and 37 ORs previously not tested [115] as expressed (Additional file 13: Figure S9b). This discrepancy may partially be due to the different type of methodology used to identify expression. However, culturing conditions and the specific genetic variations of the strain used may also be responsible for the differences.

Taking both studies together, there is clear experimental evidence for 191 ORs that are expressed in the adult head. By including adult leg and all adult body data, 223 ORs seem to be expressed in total, of which 17 actually do not encode an intact OR. However, for 64 OR functional gene models, no expression could be detected so far. This may be due to low expression in a single OSN or conditional expression under exceptional circumstances. The red flour beetle can live for up to two years [168]. During this long period in their natural environment, the beetles can encounter a variety of challenges such as food shortages, which possibly triggers flight migrations over tens of kilometers [169]. Under such exceptional circumstances, the not or low-expressed receptor genes may become active [115], as shown in studies in *D. melanogaster* [170] and *An. gambiae* [171] where up to fivefold upregulation of several ORs was triggered by temperature or feeding state.

### Inter-species comparison of olfactory components

The comparison of the number of main components of the chemosensory pathway of different insect species reveals the high diversity of evolutionary strategies to enable proper chemoreception and thus, reflects the diversity of insects and the manifold adaptations to their specialized lifestyles (Table 1). In particular, *T. castaneum* has by far the lowest number of chemoreceptive sensilla (154) and consequently also of CSNs (720). In contrast to this low number, the number of GRs (220) and ORs (341), but not of the IRs (23) encoded in the genome is exceptionally high. The number of olfactory glomeruli in the AL is within the range of most other species (Table 1) [57, 172]. Comparing the relation of OR genes and number of glomeruli, the highest discrepancy occurs with about fourfold higher numbers of OR genes in *T. castaneum*. However, also in *Aedes aegypti*, OR gene numbers are more than double the number of glomeruli [165]. In most other analyzed insects, except ensiferan orthopterans that have hundreds of microglomeruli [173, 174], the number of OR genes is typically similar to the number of glomeruli (Table 1). Despite the relatively low number of IRs encoded in the genome of *T. castaneum*, the repertoire of IRs involved in olfaction is highly conserved (Fig. 8). The number of KCs is roughly the same as in *D. melanogaster* and seems to be independent of the OR or AL glomeruli number (Table 1) [175].

**Table 1:** Comparison of main components of the olfactory system of different insect model organisms

Species	Chemo-receptive sensilla (per antenna)	CSNs (per antenna)	IRs (genes)	GRs (genes)	ORs (genes)	AL glomeruli (per AL)	KCs (per MB)
*T. castaneum*	154	720	23^1^	220^2^	341^3^	70^4^ – 90	2 700
*D. melanogaster*	530^5^	1 200^5, 6^	66^1^	73^7^	62^8^	43^6^ – 54^9^	2 500^10^
*An. gambiae*	714 f^11a^	1 500 – 1600 f^11a^	46^1^	60^7^	79^8^	60 f^14^	n/a
	738 f^12^				76^13^	61 m^14^	
*Ae. aegypti*	928 f^15^	1946 f^15^	95^1^	79^16^	131^17^	50 f^18^	n/a
						49 m^18^	
*Bombyx mori*	>24 500 m^19a^	50 000 m^19a^	18^1^	56^7^	48^7^	55 – 60^19^	n/a
	>21 000 f^19a^	30 000 f^19a^					
*M. sexta*	190 000 m^20^	255 000 –	21^22^	45^22^	71^22^	63^23^	n/a
		450 000 m^21a, 20^					
		169 000 f^21a^					
*A. mellifera* (worker)	5 000 – 5 100^24^	60 000^25a^	10^1^	53^7^	163^8^	156 –166^26^	170 000^27^ – 184 000^28^
		63 700^24^					
*Periplaneta americana*	65 500 m^29^	241 000 m^29^	n/a	n/a	n/a	125 f^29^	175 000^30^
						126 m^29^	
Locusts	*Lmig* 4 700^31^	50 000^32a^	*Lmig* 11^7^	*Lmig* 75^7^	*Lmig* 95^7^	*Sgre* 1 000^32^	*Sgre* 50 000^32^
						*Sgre* 2 500 – 3 000^33^	

*AL antennal lobe, CSN* chemosensory neuron, *f* female, *GR* gustatory receptor, *IR* ionotropic glutamate-like receptor, *KC* Kenyon cells, *Lmig Locusta migratoria*, *m* male, *n/a* not available, *OR* odorant receptors, *Sgre Schistocerca gregaria*

^a^Olfactory sensory neurons/sensilla (otherwise chemosensory neurons/sensilla).

^1^Croset et al. 2010 [114], ^2^Richards et al. 2008 [81], ^3^Engsontia et al. 2008 [115], ^4^Dreyer 2010 [200], ^5^Stocker 2001 [59], ^6^Vosshall and Stocker 2007 [137], ^7^Wang et al. 2014b [223], ^8^Sánchez-Gracia et al. 2001 [25], ^9^Grabe et al. 2015 [224], ^10^Hinke 1961 [225], ^11^Qiu et al. 2006 [226], ^12^Pitts and Zwiebel 2006 [227], ^13^Pitts et al. 2011 [128], ^14^Ghaninia et al. 2007 [160], ^15^McIver 1978 [228], ^16^Kent et al 2008 [229], ^17^Bohbot et al. 2007 [165], ^18^Ignell et al. 2005 [162], ^19^Koontz and Schneider 1987 [230], ^20^Lee and Strausfeld 1990 [231], ^21^Homberg et al. 1989 [232], ^22^Kanost et al. 2016 [233]^, 23^Rospars and Hildebrand 2000 [234], ^24^Esslen and Kaissling 1976 [235], ^25^Frasnelli et al. 2010 [236], ^26^Galizia et al. 1999 [237], ^27^Mobbs 1982 [238], ^28^Strausfeld 2002 [239], ^29^Boeckh and Ernst 1987 [240], ^30^Neder 1957 [241], ^31^Greenwood and Chapman 1984 [242], ^32^Laurent and Naraghi 1994 [243], ^33^Schachtner et al. 2005 [57]

### No apparent sexual dimorphism

Sexual dimorphism of the olfactory system is described in many insect species [57, 172, 176, 177]. However, in contrast to other coleopterans [178–182], our analysis revealed no apparent sexual dimorphism on antenna morphology or number and distribution of sensilla (Additional file 1: Figure S1b–d). Expression analysis of male and female antenna samples revealed only a small but not significant dimorphism in the OBP expression levels described earlier [89]. Also for IRs, GRs, ORs, and SNMPs, we could not find any significant sexual differences (Fig. 6), similar to the striped flea beetle *Phyllotreta striolata* [183] and in contrast to described situations in Diptera and Lepidoptera [63, 128, 184, 185]. Different numbers of glomeruli or different sized glomeruli were observed in several insect species [57, 70, 176] including the beetle *Holotrichia diomphalia* [172]. However, the comparison of the ALs of *T. castaneum* males and females disclosed no obvious dimorphism as previously described also for the small hive beetle (*Aethina tumida*) [186]. In summary, our study did not reveal any sexual dimorphism of the olfactory system in *T. castaneum*. This finding is consistent with behavioral studies that showed an attraction of both sexes to the aggregation pheromone 4,8-dimethyldecanal [187] and no sex preference in the mating choice of males [188].

## Conclusions

Detailed analysis of the olfactory system in *T. castaneum*, a holometabolous insect of special importance for the study of coleopteran and pest biology, reveals that olfactory sensory input from the antennae is processed mostly in the antennal lobes of the brain, as observed in other insect species. However, tracing of olfactory projections from the mouthparts enabled the identification of two additional neuropils: a lobus glomerulatus described previously only in a hemimetabolous insect and an unpaired glomerularly organized olfactory neuropil in the GNG (the GOC), which has never before been described. In addition, the high number of GRs on both the antennae and mouthparts indicates that there is no organotopic separation of olfaction and gustation in this beetle. These findings are a reminder of the wide variety of solutions to chemoreception that have evolved in the holometabolous insects. This should remind us that we have much still to learn about olfactory systems in general.

## Methods

### *Tribolium castaneum* rearing and transgenic lines

*Tribolium castaneum* (Herbst, 1797; Insecta, Coleoptera, Tenebrionidae) wild-type strain San Bernardino, as well as the transgenic lines partial *Orco*-Gal4, *UAS*-DsRed, *UAS*-tGFP [87], and *EF1*-*B*-DsRed [189] were bred at about 30 °C and 40 % relative humidity on organic whole wheat flour supplemented with 5 % yeast powder [190]. The *Orco*-Gal4 and *UAS*-DsRed lines were generated by *piggyBac*-based insertional mutagenesis [191]. The donor plasmids used were assembled by a versatile two-step cloning procedure [192].

For the partial *Orco*-Gal4 line, a donor plasmid was generated by cloning a blunted and BamHI (Fermentas, Vilnius, Lithuania) digested PCR < product containing *Gal4delta*-*SV40pA* (amplified with primers Gal4deltafor and SV40rev from plasmid CH#757, see Additional file 16) into the BamHI and EcoRV (Fermentas) digested pSLfa1180 vector [193]. After propagation, a BamHI and BfuAI digested PCR product containing 2.5 kb upstream of the *TcasOrco* (amplified with TcOR1upfor and TcOR1uprev from San Bernardino gDNA) was cloned into the corresponding restriction sites to generate pSLfa1180[2.5kbOrcoUp_GAL4delta]. The whole cassette was shuttled with AscI and FseI (New England Biolabs, Ipswich, MA, USA) into the pBac[*3XP3*-*Tcv*] [194] donor plasmid. The tissue-specific expression of Gal4 in the *Orco*-Gal4 line was determined by crossing it with an *UAS*-tGFP [87] line and performing IHC on the antennae with α-tGFP and α-Orco antibody or by staining of the whole brain with α-tGFP and an α-synapsin counterstaining. These stainings revealed that only Orco-immunoreactive neurons are labeled in antennae (Additional file 2: Figure S2d), which indicates the specificity of the Orco-Gal4 driver line. However, only half of the Orco-immunoreactive neurons in the antenna express tGFP (Additional file 2: Figure S2d), which implies that the *Orco*-Gal4 line only partially covers the Orco pattern resulting in labelling of only half of the AL glomeruli (Additional file 2: Figure S2e). The same approach with an *UAS*-dsRed line and an α-RFP antibody was used to characterize the palps, in which the reporter is also exclusively expressed in *Orco*-immunoreactive neurons, but in only 10–20 % of the cells (Additional file 2: Figure S2f). We, therefore, refer to it as the partial *Orco*-Gal4 line.

For *UAS*-DsRed**,** the donor plasmid pBac[*3XP3*-eYFP_*UAS*-*Tchsp68bP*-DsRedex-*SV40*] was generated by cloning the DsRed express ORF (Clontech laboratories Inc., Mountain View, CA, USA; catalog no. 632412) into the pSLfa[*UAS*-*Tc'Hsp-p*-tGFP-*SV40*]fa shuttle vector [87] by using KpnI and NotI, which was followed by transferring the *UAS*-*hsp*-DsRed-SV40 cassette into the pBac[*3XP3*-eYFP] [193] using AscI and FseI. The *UAS*-DsRed line as well as the *UAS*-tGFP line were analyzed by confocal microscopy to ensure that no reporter expression was present in the relevant tissues in the absence of a Gal4 driver line (Additional file 17: Figure S12).

The EF1-B-DsRed line (elongation factor1-alpha regulatory region-DsRedExpress; kindly provided by Michalis Averof, Institut de Génomique Fonctionnelle de Lyon, France) has been described to label most neurons in the central nervous system of first instar larvae [189] and also shows high expression in the adult central nervous system. However, clearly not all neurons are labeled in the peripheral nervous system. We, therefore, re-analyzed adult antennae of this line using confocal microscopy in combination with antibody stainings. The labeled neurons in the antenna resemble the typical morphology of CSNs with the dendrites being embedded in the sensilla cavities (Fig. 2h; Fig. 1f, g, and h) and the axons converging to the antennal nerve (Fig. 2h; Additional file 1: Figure S1a). No labelling was detected at mechanosensory sensilla (Fig. 1c, d, and e) except the scolopidia cells of Johnston's organ (Additional file 1: Figure S1a). In addition to almost all Orco-immunoreactive ORNs (Fig. 2i), this line labels also non Orco-immunoreactive neurons that are affiliated with sensilla coeloconica (Fig. 1h) and sensilla basiconica (Fig. 1g). Whereas in the palps only about 30 to 50 % of the DsRed-immunoreactive cells are also Orco-immunoreactive (Fig. 4), in antennal segment 11 a higher percentage of CSNs is double labeled, and in segments 9 and 10, the vast majority of CSNs are double labeled (Fig. 2i–i''; Additional file 1: Figure S1a). This suggests that almost all and only CSNs are labeled by this line in the adult antenna.

### Tissue preparation for SEM

Antennae of sex-separated adults were dissected and immediately fixed for at least 2 h in 5 % glutaraldehyde in 0.1 M phosphate buffered saline (PBS), pH 7.1, washed and post-fixed in osmium-tetroxide (1 % in 0.1 M Sörensen buffer, pH 7.2). Fixed samples were washed in water, dehydrated overnight in ethyleneglycolmonoethylether, and then transferred into acetone via at least three 10-min changes with 100 % acetone as described in [195]. The samples were critical-point-dried by using a Polaron E 3000 (Balzers Union, Quorum Technologies Ltd, Darmstadt, Germany). After being sputtered with gold (Balzers Union Sputter Coater, Balzers, Liechtenstein; Quorum Technologies Ltd, Ringmer, UK), the material was examined using a Hitachi S-530 SEM (Hitachi High-Technologies Europe GmbH, Krefeld, Germany). Micrographs (Figs. 1b–b'', c''', c'''', d''', d'''', e''–e'''', f'', g''', g'''', h'', and 2a–g) were taken by digital image acquisition (DISS 5, point electronic, Halle, Germany).

### Immunohistochemistry

Whole mount brain IHC was performed as described in [102]. The animals were cold anesthetized, their brains were dissected in cold PBS (0.01 M, pH 7.4), and fixed subsequently overnight at 4 °C or for 1–2 h at room temperature in PBS containing 4 % paraformaldehyde (Roth, Karlsruhe, Germany). The tissue was rinsed four times for 10 min with PBS. and pre-incubated with 5 % normal goat serum (NGS, Jackson ImmunoResearch, Westgrove, PA, USA) in PBT (PBS containing 0.3 % Triton X-100; Sigma-Aldrich, Steinheim, Germany) for 1–3 days at 4 °C. After pre-incubation, nervous tissue was transferred to the primary antibody solution containing 2 % NGS in PBT and incubated for 2–4 days at 4 °C. To label neuropil regions selectively, a monoclonal primary antibody from mouse against synapsin was used in combination with specific additional antibodies and various dyes (for an overview of the antibodies and dyes employed, see Additional file 18: Table S2). After rinsing five times for 10 min with PBT, the brains were incubated with appropriate secondary antibodies and various dyes (Additional file 18: Table S2) diluted in PBT containing 2 % NGS for 1–3 days at 4 °C, followed by 3 to 5 washing steps for 10 min each with PBT. Brains and ganglia were dehydrated in an ascending ethanol series (50 %, 70 %, 90 %, 95 %, 100 %, and 100 % for 2.5 min each) and cleared with methyl salicylate (Merck, Gernsheim, Germany). Finally, they were mounted on coverslips using Permount mounting medium (Fisher Scientific, Pittsburgh, PA, USA) and a stack of two reinforcement rings (Zweckform, Oberlaindern, Germany) as spacers to prevent compression. Brains and ganglia of some of the backfills were not dehydrated and directly mounted in Aqua-Poly/Mount (Polysciences Europe Inc., Eppelheim, Germany).

Antennae and palps of the *EF1*-*B*-DsRed, the *Orco*-Gal4/*UAS*-tGFP, or *Orco*-Gal4/*UAS*-dsRed lines were dissected and fixed overnight at 4 °C in 4 % paraformaldehyde and 10 % methanol in PBT. Afterwards, they were transferred into silicone molds, embedded in tissue-freezing media (Leica, Wetzlar, Germany), and frozen for at least 1 hour at -80 °C, followed by cutting into 50 μm sections at -23 °C on a Cryotome (Cryotome CM 1959, Leica Microsystems, Wetzlar, Germany) resulting in longitudinally halved antennae. The half mounts were collected in a tube and rinsed four times for 20 min each at room temperature in PBT. The samples were pre-incubated with 5 % NGS in PBT overnight at 4 °C followed by incubation with primary antibodies and dyes together with 5 % NGS in PBT overnight. After washing four times for 20 min with PBT, the samples were incubated with appropriate secondary antibodies (Additional file 18: Table S2) overnight at 4 °C. Finally, the antennae were rinsed four times with PBT for 20 min and embedded on coverslips in Aqua-Poly/Mount with one layer of reinforcement rings as spacers.

The specificity of the Orco-antiserum (Moth-R2, kindly provide by Jürgen Krieger) in *T. castaneum* could be demonstrated by IHC on antennae of animals with RNA interference-mediated knock-down of *Orco* [115]. To circumvent problems during dsRNA synthesis previously observed with the full length CDS of *TcasOrco*, we cloned a 476 bp fragment from San Bernadino cDNA containing only a part of CDS and the majority of the 3' untranslated region amplified by Advantage2 Taq Polymerase and primers *TcasOrco3UTRrev* and *Tcas*Orco3for (see Additional file 16) into PCRII vector (Invitrogen). Using PCR, a bidirectional template was generated followed by dsRNA synthesis with the MEGAscript T7 transcription kit (Ambion, Austin, USA) [196]. *Orco dsRNA* was injected into pupa of the strain San Bernardino. About 7 days after adult eclosion, the antennae of the treated animals were collected together with antennae of untreated beetles of the black strain, which can be easily discriminated based on the cuticle color, and thus, they served as internal staining controls. A maximal projection of a confocal stack of the Orco-antiserum (Moth-R2) treated antennae shows no detectable antibody staining in RNAi-treated animals (Additional file 2: Figure S2b) in contrast to the black beetle internal control (Additional file 2: Figure S2c).

### In vivo backfills of the antenna, single maxillary palps, and whole mouthparts

Cold anesthetized animals were mounted with dental wax (S-U-wax wire, 2.0 mm, hard; Schuler Dental, Ulm, Germany) and modelling clay (Das grosse Dino-Knet-Set; Moses, Verlag GMBH, Kempen, Germany) using a low-temperature soldering iron (Solder-Unit ST 081; Star Tec Products, Bremen, Germany) or with rubber cement (Fixogum, Marabu, Tamm, Germany) with their dorsal side on a microscope slide. The last three segments of the antenna and the most distal segment of the maxillary palp were removed and 4 % neurobiotin in 1 M KCl (Vector Laboratories, Burlingame, UK) for the antenna and Texas Red coupled dextran 50 mg/ml in PBS (3000 MW; Molecular Probes, Invitrogen) for the maxillary palps were used as neuronal tracers. Glass micropipettes were drawn (Model P-97, Sutter Instrument, Novato, USA) from borosilicate glass (inner diameter, 0.75 mm; outer diameter, 1.5 mm; Hilgenberg, Malsfeld, Germany) and broken to a tip diameter matching to the antenna/maxillary palp stump. The dye-filled glass micropipette was put on the antenna/maxillary palp stump for about 4–6 hours in a moist chamber at 4 °C. For the backfills of the whole mouthparts, the maxillary and labial palps were cut and the antennae were protected from unintentional dye-filling by covering them with dental wax (S-U-wax wire, 2.0 mm, hard). A crystal of biotin-conjugated dextran (3000 MW; Molecular Probes, Invitrogen) was placed onto the prepared mouthparts, covered with a drop of distilled water, and stored for about 4 h in a moist chamber at 4 °C. Brains and ganglia were dissected, fixed, washed, and stained as described above. Neurobiotin was visualized with Cy3 conjugated streptavidin (Dianova, Hamburg, Germany) diluted 1/200 in PBT (0.3 % TrX). The staining solution contained in addition Alexa Fluor 488-coupled phalloidin (1/200), DAPI (1/20,000) and 2 % NGS. The incubation time was 2–3 days at 4 °C. Biotin-coupled dextran was visualized with Alexa Fluor 488-coupled streptavidin (Molecular Probes, Invitrogen) diluted 1/200 in PBT (0.3 % TrX and 2 % NGS) and applied together with synapsin (1/300) for 2–3 days at 4 °C.

### In vivo dye injection into the antennal lobes

Cold anesthetized animals with fluorescent labeled ALs (partial *Orco*-Gal4/*UAS*-DsRed) were mounted with their ventral side pointing upside down with dental wax on a microscope slide. The pronotum and the head capsule were opened using a piece of a razor blade held by a blade breaker, with two parallel longitudinal cuts along the compound eyes. The cuticle, fat tissue, and tracheae were removed. Afterwards, the head capsule and pronotum were covered with ringer solution [197]. A tungsten needle was sharpened in 2 M KOH with 5–8 volts as described in [198], followed by coating with Texas Red conjugated dextran (3000 MW; Molecular Probes, Invitrogen) dissolved in NGS and air-dried. The injection of dye into the DsRed-labeled AL was performed manually under a fluorescence stereomicroscope (SteREO Lumar.V12, Carl Zeiss MicroImaging, Jena, Germany) by careful perforation. The treated animals were kept in a moist chamber for about 1 h at room temperature to let the dye diffuse. Afterwards, the brains were dissected, fixed, washed, and pre-incubated with NGS as described previously and afterwards incubated with Alexa Fluor 488-coupled phalloidin (1/200), DAPI (1/20,000), and 2 % NGS for 2 days at 4 °C. Subsequently, the brains were washed, dehydrated, cleared, and mounted in Permount as described above.

### Microscopic image acquisition, processing, and analysis

The fluorescent-labeled microscopic samples were scanned with CLSM (TCS SP5, Leica Microsystems) at 1024 × 1024 or 2048 × 2048 pixel resolution, a scanning speed between 100 and 200 Hz, a pinhole of size 1 airy, a line average of 2–4, and a step size between 0.5 and 2.5 μm. Confocal images and image stacks were analyzed with the Amira 5.3.3 graphics software (FEI, Hillsboro, OR, USA). The final image processing and figure arrangements were processed using Corel Draw X3 (Corel, Ottawa, Ontario, Canada), Adobe Photoshop CS3 (Adobe Systems, San Jose, CA, USA), or Inkscape [199].

The number of CSNs per sensillum was determined based on high-resolution CLSM stacks taken from antennae of the *EF1*-*B*-DsRed line after antibody enhancement of the DsRed reporter signal in combination with Orco antibody staining. To determine the number of CSNs and Orco-immunoreactive OSNs, we traced the stained dendrites of the CSNs to their associated soma of several sensilla and calculated their average number (Additional file 3: Figure S3i; Fig. 2h).

AL glomeruli were separately labeled in the AMIRA Segmentation Editor and 3D reconstructed [102, 200] based on CLSM stacks of brains labeled with synapsin and TKRP antibodies of five male and five female A7 beetles (one AL from a random hemisphere for each brain). To optimize data quality, the CLSM stacks were previously deconvoluted in AMIRA using the blind method with initial estimation set to input data, with a border width of 10, 10, and 10, and an iteration of ten cycles.

KCs were identified based on their position, size, and density in DAPI stainings [103]. The total volumes of the whole CAs (13 CAs of seven A7 males), as well as the volumes of three randomly assigned clusters of 20 KCs per CA, were measured using 3D reconstruction. For the segmentation and reconstruction details, we refer to [201, 202]. Briefly, different layers of a structure were labeled in the Segmentation Editor and wrapped. Volumes of reconstructed structures were taken from Material Statistics. Based on the ratios between whole CA volume and the volumes of the three clusters of 20 KCs, the total number of KCs per CA was interpolated. In addition, we counted the KCs by an independent method using MorphoGraphX (www.MorphoGraphX.org). The CLSM stacks were processed with the arithmetic tool of AMIRA to mask the CAs and consequently to remove the remaining materials. The resulting stacks were converted to TIFF files with FIJI [203] by preserving the image properties. These files were analyzed with the “Local Maxima” tool of MorphoGraphX [111] with the following parameter X-/Y-/Z-radius = 1 μm, Start Label -1, and Min Color 1. Because of the inhomogeneous intensity distribution within some CLSM stacks, only nine CAs from five specimens were analyzed automatically.

Statistical analysis included determination of mean values, standard deviations, and independent/unpaired two-tailed Student’s *t*-tests, which were performed in Excel XP (Microsoft, Redmond, WA, USA). Bar charts were created in Excel XP and imported and revised in Corel Draw.

### RNA isolation and sequencing

Total RNA of about 1000 antennae, 150 mouthparts (a piece of the head capsule anterior of the antennae), 600 legs, 50 heads (without antennae but including mouthparts), and 20 remaining bodies of sex-separated adults was isolated using the ZR Tissue and Insect RNA Micro Prep Kit (Zymo Research, Irvine, CA, USA), following the manufacturer´s protocol. The library preparations for RNA-Seq were performed using the TruSeq RNA Sample Preparation Kit (Illumina, San Diego, CA, USA) and cDNA libraries were amplified and sequenced using the cBot and HiSeq2000 from Illumina (paired end; 2 × 100 bp). Biological triplicates of female and male antennae were sequenced. For the other tissues, one male and one female sample were used and one additional mouthpart sample was obtained from unsexed beetles. The number of biological replicates was chosen based on previous publications using similar approaches [128, 170, 171]. Each biological replicate came from independently prepared and processed tissue. No technical replicates were performed. For details, see Dippel et al. [89].

### Re-annotation of olfactory genes

For manual inspection the obtained reads were mapped against the *T. castaneum* 4.0 genome using BLAT [204] and a genome browser was set up (http://bioinf.uni-greifswald.de/tcas/). In a genome independent approach, a de novo assembly was built with Trinity (release 2013_08_14) [205] as described in [89]. The previously published OR [115], GR [81], IR [114], and SNMP [116, 117] sequences were used for further analysis. To identify the potential ODEs, the official (OGS3) [81–83], the preliminary AU2 and AU3, and the NCBI [206, 207] gene sets were used and a protein functional analysis was conducted using InterProScan [208]. All genes belonging to a protein family containing known ODEs in other insect species were collected (namely, ALDH, ALOX, CES, EH, GST, and CYP [4]). The redundant genes were removed and the sequences were reviewed. The identified GSTs and CYPs were collated to already published sequences [148, 150] and the names were adapted. For all other candidates, a genome-based name was built reflecting the protein family and the chromosomal localization (e.g., CES2D is the fourth CES on the second chromosome). The genes supposed to be involved in olfactory transduction of *D. melanogaster* were taken from [46], the corresponding sequences were downloaded from the Flybase [209], and the *T. castaneum* orthologs were identified by pBLAST embedded in the genome browser (http://bioinf.uni-greifswald.de/tcas/).

The revision of the olfactory genes was performed in an iterative process based on sequence comparison with the de novo assembly and the RNA-seq based gene annotations (AU3), a conserved domain search [210], and manual inspection of the aligned reads in the genome browser. For discrepancies, the gene models were manually curated. Finally, the chromosomal localization of the olfactory genes was determined by pBLAST against the genome assembly Tcas4.0. The ODE candidates were searched for signal peptides using the SignalP4.1 server [211]. The sequences and read numbers are summarized in Additional file 8: Table S1. The complete dataset including all relevant parameters has been deposited in the National Center for Biotechnology Information (NCBI) database repository ‘Gene Expression Omnibus’ (GEO accession number: GSE63162).

### *Tribolium castaneum* expression profiling

The olfactory genes were identified in the AU3 gene set by pBLAST and the corresponding gene models were replaced with the re-annotated candidate sequences. The resulting enhanced AU3 gene set was used to map the RNAseq data with Bowtie2 [212] using the very sensitive presetting. The mapped reads were counted with Samtools [213] and normalized as RPKM values. The RPKMs were visualized (matrix2png interface, version 1.2.1; [214]) and the figures were arranged in Inkscape [199]. One male antennae sample was excluded from the subsequent analysis due to massive differences with the other five antennae samples in the principal component as well as cluster analysis. Because no significant differences for the genes of interest were observed between male and female reads, the sequenced tissues were considered as biological replicates. Statistical analysis was performed in R [215] with the DESeq package (version 1.12.0) [216] from Bioconductor [217]. *p* values were calculated with a negative binomial test using raw read counts and adjusted for multiple testing with the Benjamini–Hochberg method. All tissues were compared to body as reference. Significant differentially expressed genes (false discovery rate <0.05) are marked with asterisks in the heat maps. However, since only two body samples served as controls, all conclusions from this should be treated as preliminary. Genes with an RPKM ≥ 0.5 were considered as specifically expressed in the tissue. The tissue comparison was visualized as Venn diagrams (e.g., Additional file 9: Figure S5a) (http://bioinformatics.psb.ugent.be/webtools/Venn/). For details, see Dippel et al. [89].

### Phylogenetic analysis and interspecies comparison

We compared the *T. castaneum* IR, GR, and OR sequences independent from each other on a protein level with data from *D. melanogaster* [46, 209] and *An. gambiae* [128, 218]. The sequences were aligned using MAFFT (v7.040b [219]) (--genafpair --maxiterate 1000 --bl 62 --op 1.53 --ep 0.123) and the phylogeny was calculated using RAxML (version 7.8.6 [220]), with the LG substitution model and GAMMA correction. The robustness of the tree topology was evaluated by 100 rapid bootstrap replications. The relative expression levels were calculated as log_2_-fold changes of antenna/body and palp (mouthpart)/body as described in [89]. The *D. melanogaster* data set was downloaded from the EMBL gene expression atlas [221], originally published in [170], and the *An. gambiae* data were obtained from [128]. The phylogenetic tree was visualized by iTOL [222] and descriptions were added using Inkscape [199].

## Additional files

Additional file 1: Figure S1. Antibody staining against DsRed and Orco of the *EF1*-*B*-DsRed line. **a** Maximum projection of a confocal image stack of a halved antenna of the *EF1*-*B*-DsRed line, with an antibody staining against DsRed and Orco and in addition DAPI. Showing Orco immunoreactivity in the last three segments and particularly in the SBas. The DsRed reporter line labels in addition the scolopidia cells of Johnston's organ (*JO*) in the pedicellus (*P*). *S*, scapus. **b** Optical section of a mechano- and chemosensillum trichoideum (*mSTri* and *cSTri*) labeled with an Orco antibody (*green*) shows immunoreactivity only within the sensillum cavity of the *cSTri*. Autofluorescence of the cuticle at 560 nm is in *blue*. **c–c''** Single optical section of a sensilla basiconica (*SBas*) in the *EF1*-*B*-DsRed (*magenta*, **b'**) line labeled with an Orco antibody (*green*, **b''**) reveals signals of both channels particularly within the cavity and at the base of the sensillum. Both channels also show autofluorescence of the cuticle. **d–d''** Optical section of two sensilla coeloconica (*SCoe*) in the *EF1*-*B*-DsRed (*magenta*, **d'**) line labeled with an Orco antibody (*green*, **d''**) reveals no specific immunoreactivity within the sensilla cavities. Both channels also show autofluorescence of the cuticle. (TIF 8703 kb)
Additional file 2: Figure S2. Orco localization in antennae and palp. **a** Fluorescent in situ hybridization against *Orco* in the club segments in a maximum projection of a halved antenna. **b, c** Specificity of the Orco antibody. IHC against Orco in antennae of **b** a San Bernadino beetle after *Orco* ^RNAi^ treatment (light cuticle, *inset in the left lower corner*) and **c** an untreated control included in the IHC (black strain identified by dark cuticle). The cross-reactive Orco antiserum results in no detectable staining in the antenna after *Orco* knockdown, whereas in the antenna of the untreated beetles, the odorant receptor neurons (*ORNs*) are clearly labeled by the antiserum (*magenta*). This indicates the specificity of the Orco antiserum against *Tcas*Orco. Counterstaining with phalloidin (*green*) and DAPI (*blue*). The Orco antibody staining was labeled with a goat anti rabbit Cy3 secondary antibody. **d–d**'' Immunohistochemical characterization of the *Orco*-*Gal*4 line in the antenna and brain. Double immune-staining against Orco (*magenta*) and tGFP (*green*) in the antennae of the partial Orco-Gal4/UAS-tGFP line revealed that only half of the Orco-immunoreactive neurons expressing tGFP (*arrow* indicates colocalization and *arrowhead* as an example for no colocalization). **e** Immunohistochemical characterization of the *Orco*-*Gal*4 line in the brain. Antibody staining against tGFP in the *Orco*-*Gal4/UAS*-tGFP line (*orange*) labels only half of the AL glomeruli represented as a 3D reconstruction (*light green*, based on a phalloidin staining). AL glomeruli not labeled are not shown. **f** Immunohistochemical characterization of the partial Orco-Gal4 line in the palps. Double immuno-staining against Orco (*green*) and dsRed (*magenta*) in the palps of the *Orco*-*Gal4/UAS*-dsRed line reveals that all genetically labeled neurons are also Orco-immunoreactive. However, in contrast to the antennae, in which about half of the Orco-immunoreactive neurons are labeled in the partial *Orco*-*Gal*4 line, only a few of the the Orco-immunoreactive odorant receptor neurons express the reporter in the palps. The Cy2 (Orco) signal is quenched by dsRed. (TIF 6221 kb)
Additional file 3: Figure S3. Comparison of sensilla type numbers on the antenna of *Tribolium castaneum* and chemosensory neurons entering the sensilla types. **a–e** Number of different sensilla types on the 11th segment of the antenna: **a** sensilla campaniformis (*Scam*: ♂ 5.4; SD 1.8; ♀ 5.4; SD 0.8), **b** spatulate bristles (*SpaB*: ♂ 10.2; SD 0.9; ♀ 10.3; SD 0.9), **c** mechanoreceptive sensilla trichoideum (*mSTri*: ♂ 36.9; SD 3; ♀ 37.6; SD 4.3), **d** chemoreceptive sensilla trichoideum (*cSTri*: ♂ 86.3; SD 9.3; ♀ 87.1; SD 6.9), **e** sensilla coeloconica (*SCoe*: ♂ 6.8; SD 1.4; ♀ 7.6; SD 1.1). **f** Amount of sensilla basiconica on the club segments (11th: ♂ 24.4; SD 1.5; ♀ 25.5; SD 1.3; tenth: ♂ 16.2; SD 1.5; ♀ 16.4; SD 1.4; ninth: ♂ 13.6; SD 0.9; ♀ 13.8; SD 1.1), regardless of the number of prongs. **g** Number of sensilla basiconica as in (**f**), but considering the prong number. **h** Number of different sensilla in the lateral corner of the tenth and ninth segments. **i** Number of chemosensory neurons (CSNs) entering the chemoreceptive sensilla: *SBas* 5.92 CSNs per prong (SD = 1.2; *n* = 73 prongs of total 48 *SBas*), *cSTri* 1.07 CSNs (SD = 0.25; *n* = 61), and *SCoe* 3.16 CSNs (SD = 1.10; *n* = 5). Error bars represent standard deviations; *n* = number of antennae. (TIF 1147 kb)
Additional file 6: Figure S4. Ipsilateral antennal projection. Maximum intensity projection of a brain labeled with an antibody against synapsin (*green*) and a neurotracer resulting from an antennal backfill (*magenta*). The antennal backfill labels exclusively structures in the ipsilateral hemisphere, mainly the AL via the antennal nerve (*), and a tract (*arrowhead*) descending to the gnathal ganglion. The *inset* depicts a projection of only a few optical sections showing fibers interconnecting the AL and the protocerebrum with some arborizations in the accessory medulla of the optical lobe (*arrow*) suggesting an integration of circadian information. (TIF 3816 kb)
Additional file 8: Table S1. Summary of the RNAseq data. In column **(A)** the gene name of the GRs according to [81], the ORs from [115], the SNMPs modified after [116, 117], the *T. castaneum* orthologous of *D. melanogaster* genes named after candidates obtained from [46], the GSTs named after [148], the CYPs named after [150] and the remaining ODE candidates de novo named according to their chromosomal localization (Additional file 15: Figure S11). Column B shows the sequences of the ORF based on published annotations or existing gene models, but modified if necessary. Confirmed gene models are highlighted in grey, modified ones are highlighted in yellow, only partially covered but expressed ones are highlighted in orange, genes with low and scattered coverage are highlighted in red, and not highlighted sequences were not manually checked. Columns C–I show the average RPKM values of antennae, mouthparts (piece of the head capsule anterior of the antennae), legs, head (without antennae but including mouthparts), and remaining body of sex-separated adult animals. Columns J–Q show the results of the statistical analysis conducted in R [215] with the DESeq package (version 1.12.0) [216] (from Bioconductor [217], based on five antenna samples and two replicates for the other adult tissues in comparison to body. Columns P–AB show the individual RPKM values of the biological replicates. (XLSX 677 kb)
Additional file 9: Figure S5.
*IR* gene tissue expression and chromosomal localization of *IR* and SNMP genes. **a** Venn diagram showing the number of *IRs* expressed (RPKM ≥ 0.5) in the different body parts: antennae, legs, mouthparts (as a piece of the head capsule anterior of the antennae), heads (the whole head capsule including mouthparts but excluding the antennae), and bodies (excluding head and legs). **b** Based on Georgia GA-2 strain genome assembly 3 [81], only chromosomal linkage groups containing an IR or SNMP are depicted. Gene clusters are indicated by a number referring to the chromosome and a letter conveys the relative position on the chromosome. The number of genes within this cluster is indicated in *square brackets*. (PDF 66 kb)
Additional file 10: Figure S6. Phylogenetic tree of IRs. *Outer rings* represent the expression in body, mouthparts (*T. castaneum*: palps, mandible, labrum, and labium; *D. melanogaster*: palp and proboscis; *An. gambiae*: maxillary palp) and antenna as a percentage compared to the highest expressed gene according to the scale in the left upper corner. Note that the methods used to obtain the different expression data (RNAseq and microarray) are not directly comparable. This figure can, thus, only give an impression of the tissue-specific abundance of the transcripts. The *scale bars* within the trees represent one amino acid substitution per site. Antennal IRs are highlighted in *yellow*. (PDF 625 kb)
Additional file 11: Figure S7.
*GR* gene tissue expression and their chromosomal localization. **a** Venn diagram showing the number of *GRs* expressed (RPKM ≥ 0.5) in the different body parts: antennae, legs, mouthparts (as a piece of the head capsule anterior of the antennae), heads (the whole head capsule including mouthparts but excluding the antennae), and bodies (excluding head and legs). **b** Based on Georgia GA-2 strain genome assembly 3.0 [81], only chromosomal linkage groups containing an IR or SNMP are depicted. Gene clusters are indicated by a number referring to the chromosome and a letter conveys the relative position on the chromosome. The number of genes within this cluster is indicated in the *square brackets*. (PDF 176 kb)
Additional file 12: Figure S8. Phylogenetic mid-point rooted tree of the *GRs* based on protein sequences. *Outer rings* represent the expression in body, mouthparts (*T. castaneum*: palps, mandible, labrum, and labium; *D. melanogaster*: palp and proboscis; *An. gambiae*: maxillary palp) and antenna as a percentage compared to the highest expressed gene according to the scale in the *left upper corner*. Note that the methods used to obtain the different expression data (RNAseq and microarray) are not directly comparable. This figure can, thus, only give an impression of the tissue-specific abundance of the transcripts. The *scale bars* within the trees represent 1 amino acid substitution per site. Potential sugar and fructose receptors are labeled and highlighted in *yellow* and in *grey*, and CO_2_ receptors are highlighted in *orange*. (PDF 1733 kb)
Additional file 13: Figure S9.
*OR* gene tissue expression and their chromosomal localization. **a** Venn diagram showing the number of *ORs* expressed (RPKM ≥ 0.5) in the different body parts: antennae, legs, mouthparts (as piece of the head capsule anterior of the antennae), heads (the whole head capsule including mouthparts but excluding the antennae), and bodies (excluding head and legs). **b** Venn diagram comparing our results (*yellow*, *green*) with data from Engsontia et al. [115] (*blue*, *red*). Number of expressed ORs, defined by RPKM ≥ 0.5 (*yellow*), by RT-PCR (*blue*), not expressed RPKM < 0.5 (*green*), or with no RT-PCR amplicon (*red*). *ORs* of the brown group were not previously tested by Engsontia et al. **c** Chromosomal localization of *T. castaneum ORs*. Based on the Georgia GA-2 strain genome assembly 3.0 [81], only chromosomal linkage groups containing an IR or SNMP are depicted. Gene clusters are indicated by a number referring to the chromosome and a letter conveys the relative position on the chromosome. The number of genes within this cluster is indicated in the *square brackets*. (PDF 277 kb)
Additional file 14: Figure S10. Phylogenetic tree of the *ORs* based on protein sequences. *Outer rings* represent the expression in body, mouthparts (*T. castaneum*: palps, mandible, labrum, and labium; *D. melanogaster*: palp and proboscis; *An. gambiae*: maxillary palp) and antenna as a percentage compared to the highest expressed gene according to the scale in the *left upper corner*. Note that the methods used to obtain the different expression data (RNAseq and microarray) are not directly comparable. This figure can, thus, only give an impression of the tissue-specific abundance of the transcripts. The *scale bars* within the trees represent one amino acid substitution per site. (PDF 2302 kb)
Additional file 15: Figure S11. Chromosomal localization of potential *T. castaneum* ODE genes. Based on Georgia GA-2 strain genome assembly 3.0 [81], aldehyde dehydrogenase (*ALDH*, in *grey*), aldehyde oxidase (*ALOX*, in *orange*), carboxylesterase (*CES*, in *blue*), epoxide hydrolase (*EH*, in *green*), glutathione S-transferase (*GST*, in *purple*), and cytochrome P450 (*CYP*, in *magenta*). Gene clusters are indicated by a number referring to the chromosome and a letter conveys the relative position on the chromosome. The number of genes within this cluster is indicated in the *square brackets*. (PDF 257 kb)
Additional file 16: Sequences of primers and template plasmid used to generate pSLfa1180[2.5kbOrcoUp_GAL4delta]. (PDF 111 kb)
Additional file 17: Figure S12.
*UAS* responder lines in the absence of Gal4 driver. In the four rows, the maximum projections of head capsules from different transgenic strains (*Orco*-Gal4/*UAS*-tGFP, *UAS*-tGFP, *Orco*-Gal4/*UAS*-dsRed, and *UAS*-dsRed) are depicted. The upper row represents the overlay of both channels (GFP/YFP in *green* and dsRed in *red*). For UAS responders without the Gal4 driver, high-resolution images of the palps and the antennal club are provided. In the second and third rows, the separated channels are given as greyscale images. The UAS-tGFP and UAS-dsRed lines do not show leaky reporter expression in the absence of a Gal4 driver in the antennae and palps. The presence of the genetic constructs is indicated by the eye markers: pBac[3XP3-dsRed_UAS-Tchsp68bP-tGFP-SV40] and pBac[3XP3-eYFP_UAS-Tchsp68bP-DsRedex-SV40]. The marker signal is quenched in the crossed lines by the vermillion rescue marker of the Orco-Gal4 construct pBac[3XP3-gVerm_2.5kbOrcoUp_GAL4delta]. (TIF 6507 kb)
Additional file 18: Table S2. Primary and secondary antibodies and dyes used with additional information such as source and specificity. *n/a* not available. (PDF 118 kb)

## Abbreviations

AL: Antennal lobe
ALDH: Aldehyde dehydrogenase
ALOX: Aldehyde oxidase
ALT: Antennal lobe tract
AMMC: antennal mechanosensory and motor center
AN: antennal nerve
CA: Calyx of the MB
CES: Carboxylesterase
CLSM: Confocal laser-scanning microscopy
CSN: Chemosensory neuron
CSP: Chemosensory protein
cSTri: Chemosensilla trichoidea
CYP: Cytochrome P450
EH: Epoxide hydrolase
GABA: Gamma-aminobutyric acid
GL: Antennal lobe glomeruli
GNG: Gnathal ganglia
GOC: Gnathal olfactory center
GR: Gustatory receptor
GSN: Gustatory sensory neuron
GST: Glutathione S-transferase
IHC: Immunohistochemistry
IR: Ionotropic glutamate-like receptor
KC: Kenyon cells
lALT: Lateral antennal lobe tract
LG: Lobus glomerulatus
LH: Lateral horn (lateral protocerebrum)
LN: Local interneuron
mALT: Mediolateral lobe tract
MB: Mushroom body
mlALT: Mediolateral antennal lobe tract
mSTri: Mechanosensilla trichoidea
NA: Neuro-axis
NGS: normal goat serum
OBP: Odorant binding protein
ODE: Odorant degrading enzyme
OR: Odorant receptor
Orco: Odorant receptor co-receptor
ORF: Open reading frame
OSN: Olfactory sensory neuron
PBS: Phosphate buffered saline
PGC: Primary gustatory center
PN: Projection neuron
RPKM: Reads per kilobase per million
SBas: Sensilla basiconica
SCam: Sensilla campaniformes
SCha: Sensilla chaetica
SCoe: Sensilla coeloconica
SD: standard deviation
SEM: Scanning electron microscopy
SNMP: Sensory neuron membrane protein
SpaB: Spaculate bristle
TKRP: Tachykinin-related peptide
