# Supplementary figures and images for: Morphological and Transcriptomic Analysis of a Beetle Chemosensory System Reveals a Gnathal Olfactory Center
Source: BMC Biol. 2016 Oct 17;14:90. doi: 10.1186/s12915-016-0304-z (PMC5067906; doi:10.1186/s12915-016-0304-z)

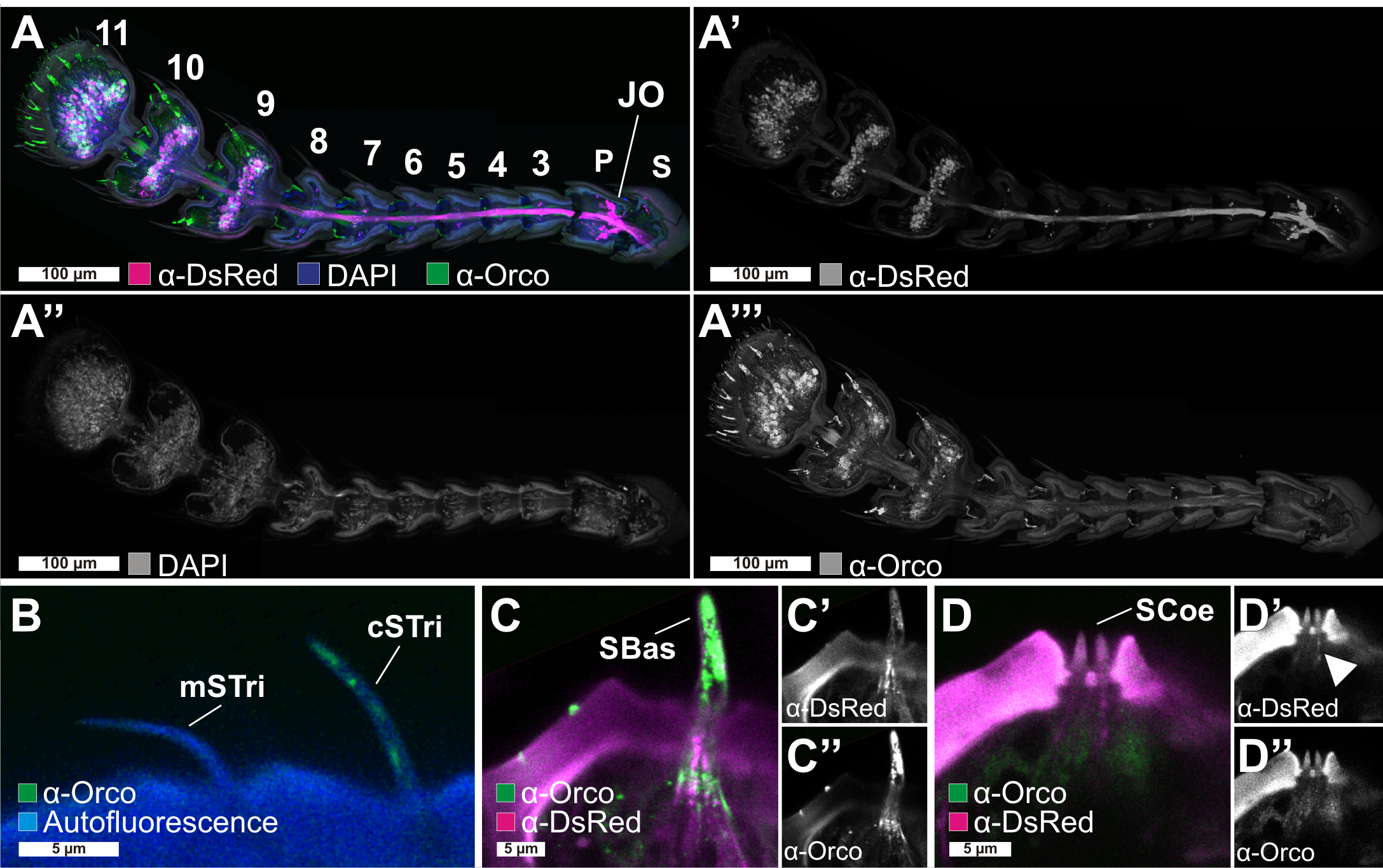

Supplement: Additional file 1: Figure S1. — Antibody staining against DsRed and Orco of the EF1-B-DsRed line. a Maximum projection of a confocal image stack of a halved antenna of the EF1-B-DsRed line, with an antibody staining against DsRed and Orco and in addition DAPI. Showing Orco immunoreactivity in the last three segments and particularly in the SBas. The DsRed reporter line labels in addition the scolopidia cells of Johnston's organ (JO) in the pedicellus (P). S, scapus. b Optical section of a mechano- and chemosensillum trichoideum (mSTri and cSTri) labeled with an Orco antibody (green) shows immunoreactivity only within the sensillum cavity of the cSTri. Autofluorescence of the cuticle at 560 nm is in blue. c–c'' Single optical section of a sensilla basiconica (SBas) in the EF1-B-DsRed (magenta, b') line labeled with an Orco antibody (green, b'') reveals signals of both channels particularly within the cavity and at the base of the sensillum. Both channels also show autofluorescence of the cuticle. d–d'' Optical section of two sensilla coeloconica (SCoe) in the EF1-B-DsRed (magenta, d') line labeled with an Orco antibody (green, d'') reveals no specific immunoreactivity within the sensilla cavities. Both channels also show autofluorescence of the cuticle. (TIF 8703 kb) [file 12915_2016_304_MOESM1_ESM.tif]

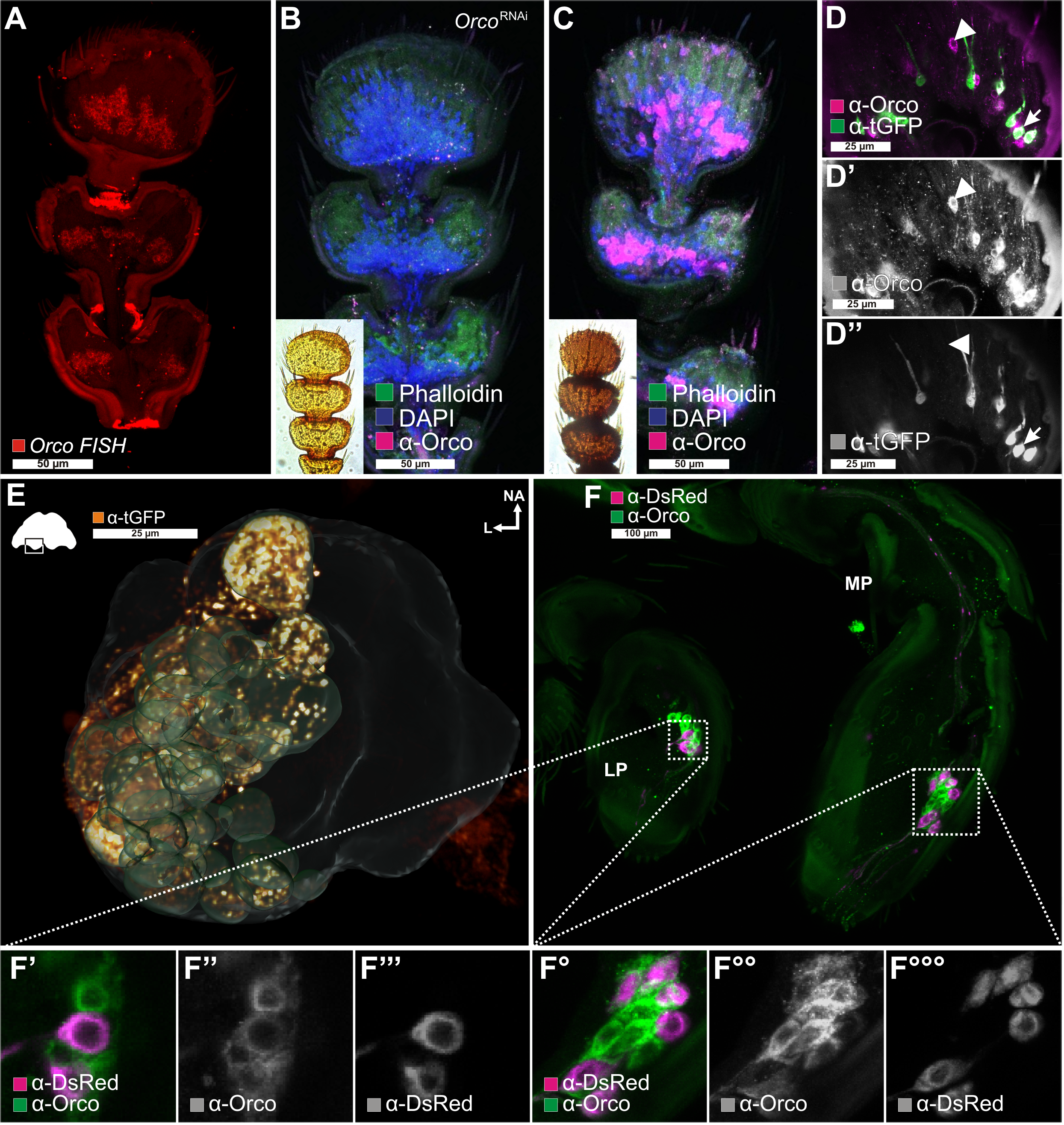

Supplement: Additional file 2: Figure S2. — Orco localization in antennae and palp. a Fluorescent in situ hybridization against Orco in the club segments in a maximum projection of a halved antenna. b, c Specificity of the Orco antibody. IHC against Orco in antennae of b a San Bernadino beetle after Orco RNAi treatment (light cuticle, inset in the left lower corner) and c an untreated control included in the IHC (black strain identified by dark cuticle). The cross-reactive Orco antiserum results in no detectable staining in the antenna after Orco knockdown, whereas in the antenna of the untreated beetles, the odorant receptor neurons (ORNs) are clearly labeled by the antiserum (magenta). This indicates the specificity of the Orco antiserum against TcasOrco. Counterstaining with phalloidin (green) and DAPI (blue). The Orco antibody staining was labeled with a goat anti rabbit Cy3 secondary antibody. d–d'' Immunohistochemical characterization of the Orco-Gal4 line in the antenna and brain. Double immune-staining against Orco (magenta) and tGFP (green) in the antennae of the partial Orco-Gal4/UAS-tGFP line revealed that only half of the Orco-immunoreactive neurons expressing tGFP (arrow indicates colocalization and arrowhead as an example for no colocalization). e Immunohistochemical characterization of the Orco-Gal4 line in the brain. Antibody staining against tGFP in the Orco-Gal4/UAS-tGFP line (orange) labels only half of the AL glomeruli represented as a 3D reconstruction (light green, based on a phalloidin staining). AL glomeruli not labeled are not shown. f Immunohistochemical characterization of the partial Orco-Gal4 line in the palps. Double immuno-staining against Orco (green) and dsRed (magenta) in the palps of the Orco-Gal4/UAS-dsRed line reveals that all genetically labeled neurons are also Orco-immunoreactive. However, in contrast to the antennae, in which about half of the Orco-immunoreactive neurons are labeled in the partial Orco-Gal4 line, only a few of the the Orco-immunoreactive odorant recept [file 12915_2016_304_MOESM2_ESM.tif]

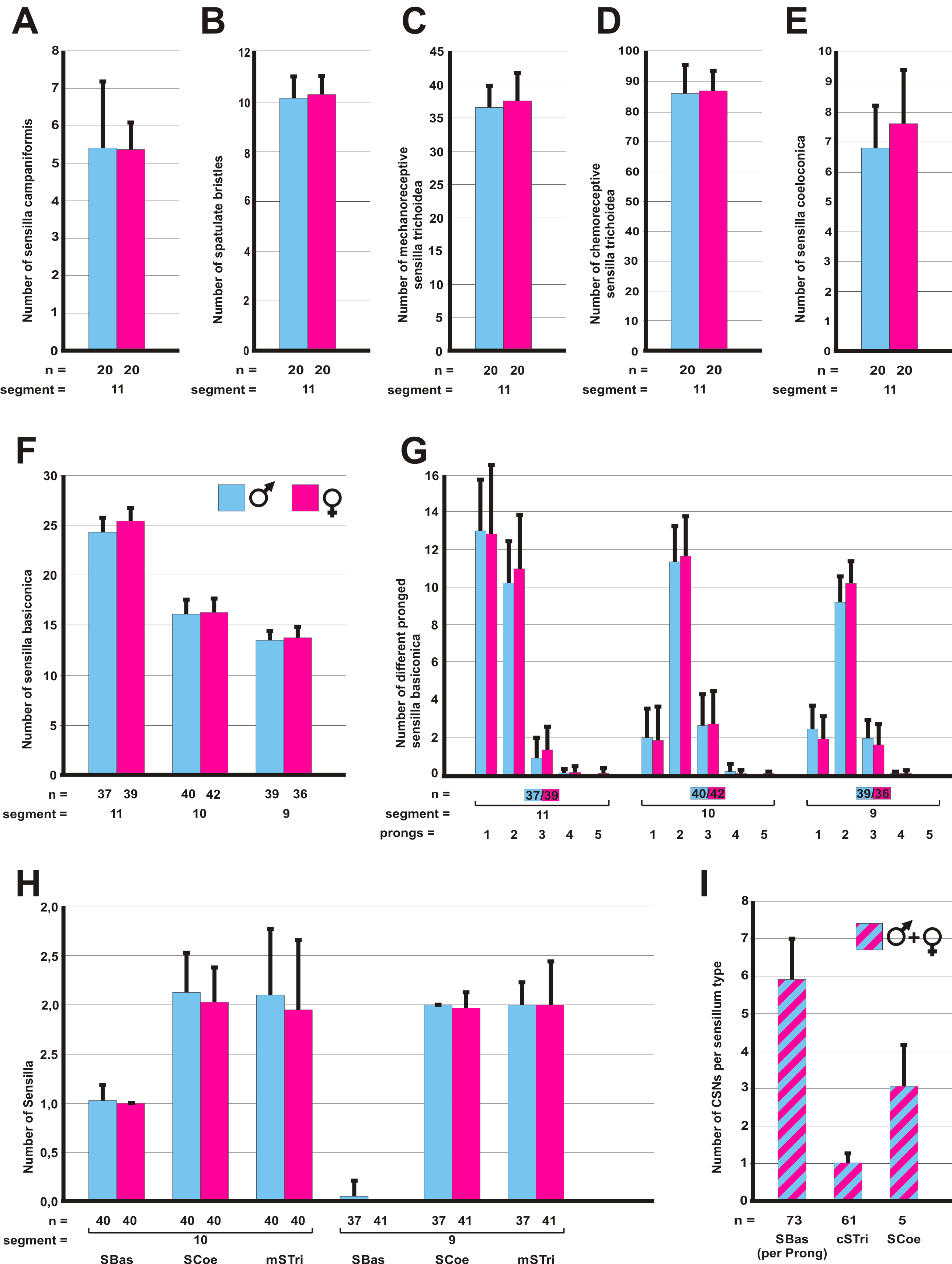

Supplement: Additional file 3: Figure S3. — Comparison of sensilla type numbers on the antenna of Tribolium castaneum and chemosensory neurons entering the sensilla types. a–e Number of different sensilla types on the 11th segment of the antenna: a sensilla campaniformis (Scam: ♂ 5.4; SD 1.8; ♀ 5.4; SD 0.8), b spatulate bristles (SpaB: ♂ 10.2; SD 0.9; ♀ 10.3; SD 0.9), c mechanoreceptive sensilla trichoideum (mSTri: ♂ 36.9; SD 3; ♀ 37.6; SD 4.3), d chemoreceptive sensilla trichoideum (cSTri: ♂ 86.3; SD 9.3; ♀ 87.1; SD 6.9), e sensilla coeloconica (SCoe: ♂ 6.8; SD 1.4; ♀ 7.6; SD 1.1). f Amount of sensilla basiconica on the club segments (11th: ♂ 24.4; SD 1.5; ♀ 25.5; SD 1.3; tenth: ♂ 16.2; SD 1.5; ♀ 16.4; SD 1.4; ninth: ♂ 13.6; SD 0.9; ♀ 13.8; SD 1.1), regardless of the number of prongs. g Number of sensilla basiconica as in (f), but considering the prong number. h Number of different sensilla in the lateral corner of the tenth and ninth segments. i Number of chemosensory neurons (CSNs) entering the chemoreceptive sensilla: SBas 5.92 CSNs per prong (SD = 1.2; n = 73 prongs of total 48 SBas), cSTri 1.07 CSNs (SD = 0.25; n = 61), and SCoe 3.16 CSNs (SD = 1.10; n = 5). Error bars represent standard deviations; n = number of antennae. (TIF 1147 kb) [file 12915_2016_304_MOESM3_ESM.tif]

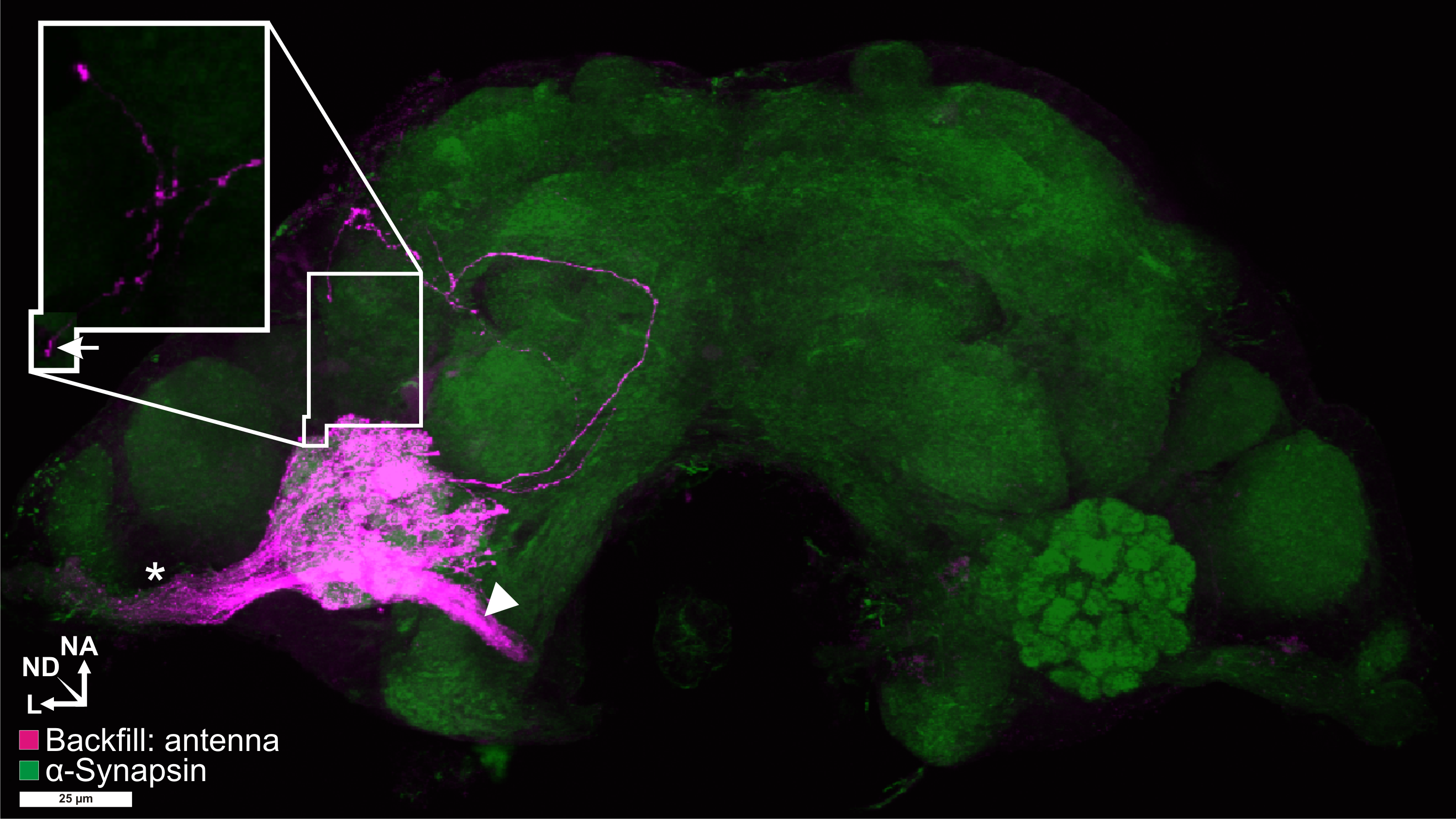

Supplement: Additional file 6: Figure S4. — Ipsilateral antennal projection. Maximum intensity projection of a brain labeled with an antibody against synapsin (green) and a neurotracer resulting from an antennal backfill (magenta). The antennal backfill labels exclusively structures in the ipsilateral hemisphere, mainly the AL via the antennal nerve (*), and a tract (arrowhead) descending to the gnathal ganglion. The inset depicts a projection of only a few optical sections showing fibers interconnecting the AL and the protocerebrum with some arborizations in the accessory medulla of the optical lobe (arrow) suggesting an integration of circadian information. (TIF 3816 kb) [file 12915_2016_304_MOESM6_ESM.tif]

**ChLGX** 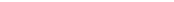 **IR21a**

ChLG4

ChLG9 IR75q.2  
IR68a

Supplement: Additional file 9: Figure S5. — IR gene tissue expression and chromosomal localization of IR and SNMP genes. a Venn diagram showing the number of IRs expressed (RPKM ≥ 0.5) in the different body parts: antennae, legs, mouthparts (as a piece of the head capsule anterior of the antennae), heads (the whole head capsule including mouthparts but excluding the antennae), and bodies (excluding head and legs). b Based on Georgia GA-2 strain genome assembly 3 [81], only chromosomal linkage groups containing an IR or SNMP are depicted. Gene clusters are indicated by a number referring to the chromosome and a letter conveys the relative position on the chromosome. The number of genes within this cluster is indicated in square brackets. (PDF 66 kb) [file 12915_2016_304_MOESM9_ESM.pdf]

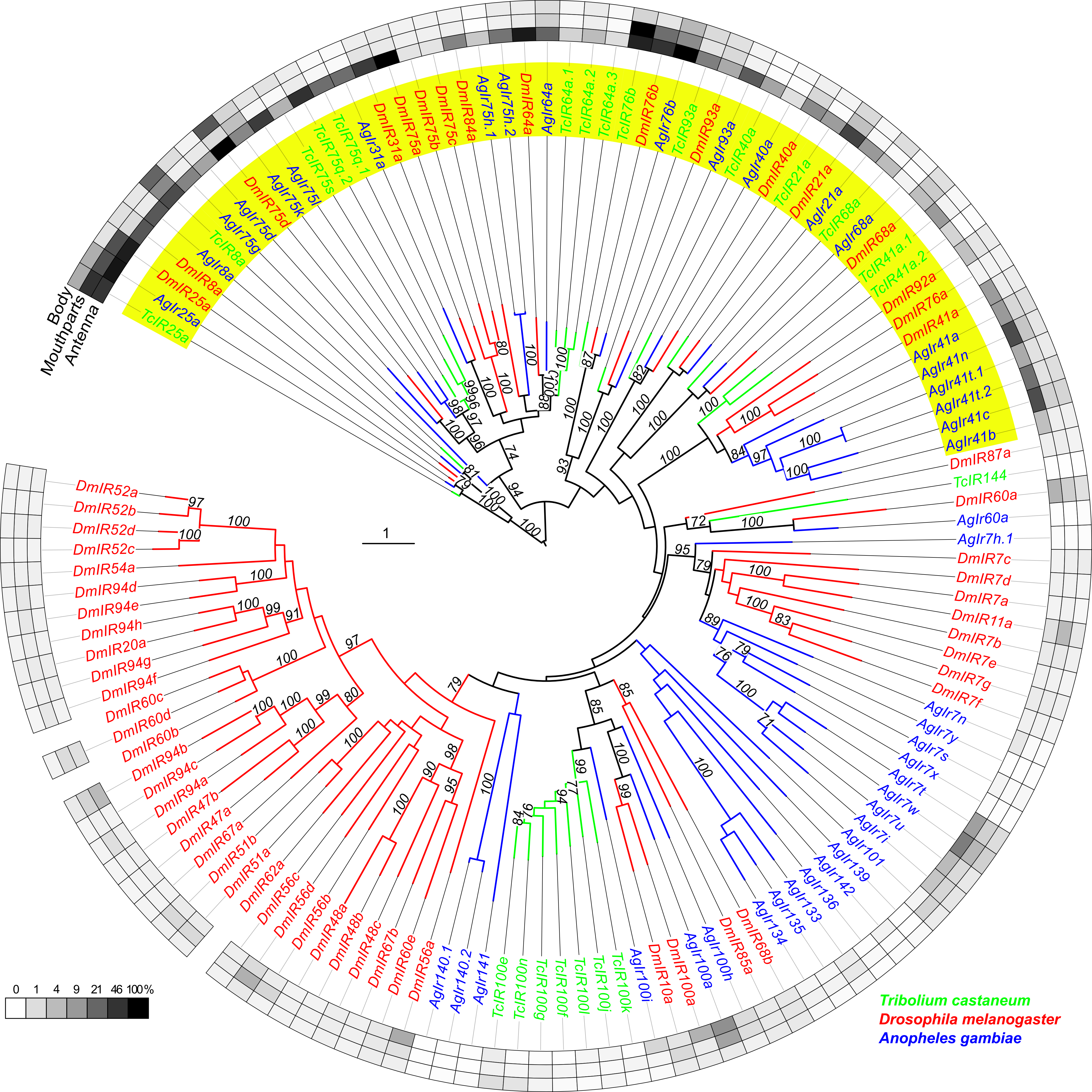

Supplement: Additional file 10: Figure S6. — Phylogenetic tree of IRs. Outer rings represent the expression in body, mouthparts (T. castaneum: palps, mandible, labrum, and labium; D. melanogaster: palp and proboscis; An. gambiae: maxillary palp) and antenna as a percentage compared to the highest expressed gene according to the scale in the left upper corner. Note that the methods used to obtain the different expression data (RNAseq and microarray) are not directly comparable. This figure can, thus, only give an impression of the tissue-specific abundance of the transcripts. The scale bars within the trees represent one amino acid substitution per site. Antennal IRs are highlighted in yellow. (PDF 625 kb) [file 12915_2016_304_MOESM10_ESM.pdf]

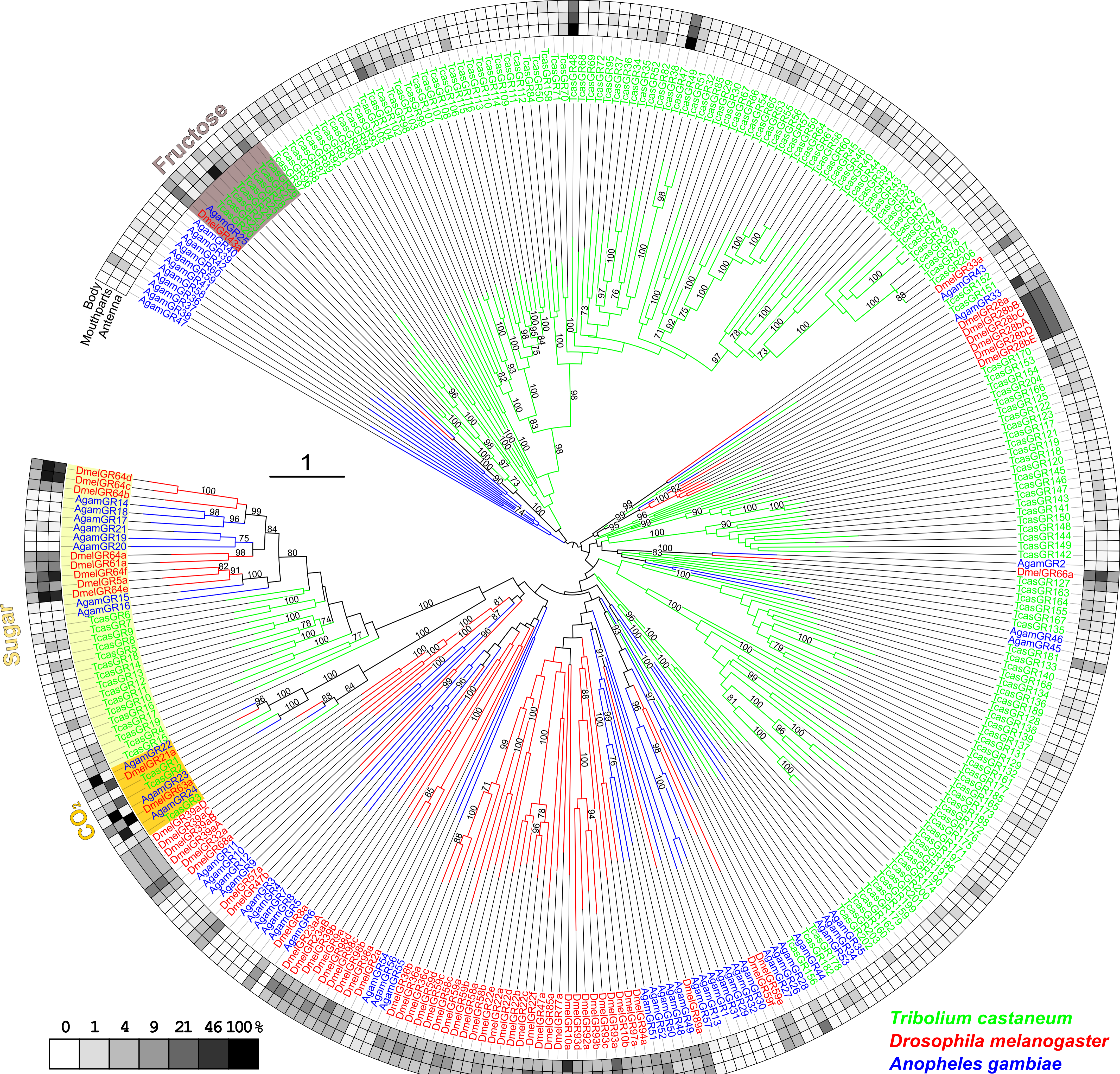

Supplement: Additional file 12: Figure S8. — Phylogenetic mid-point rooted tree of the GRs based on protein sequences. Outer rings represent the expression in body, mouthparts (T. castaneum: palps, mandible, labrum, and labium; D. melanogaster: palp and proboscis; An. gambiae: maxillary palp) and antenna as a percentage compared to the highest expressed gene according to the scale in the left upper corner. Note that the methods used to obtain the different expression data (RNAseq and microarray) are not directly comparable. This figure can, thus, only give an impression of the tissue-specific abundance of the transcripts. The scale bars within the trees represent 1 amino acid substitution per site. Potential sugar and fructose receptors are labeled and highlighted in yellow and in grey, and CO2 receptors are highlighted in orange. (PDF 1733 kb) [file 12915_2016_304_MOESM12_ESM.pdf]

A

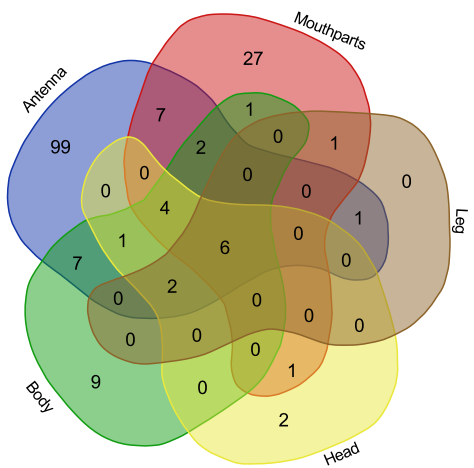

B

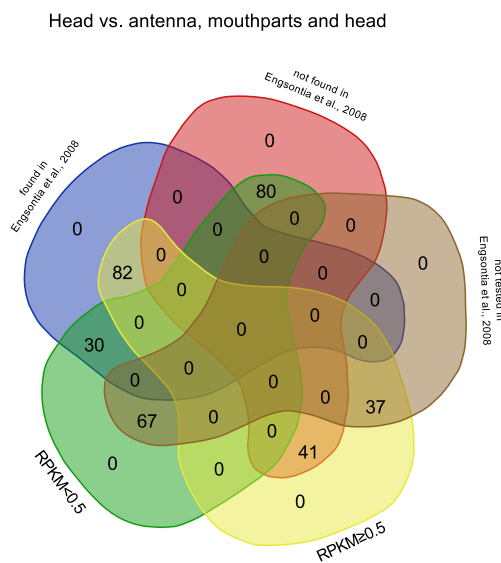

C

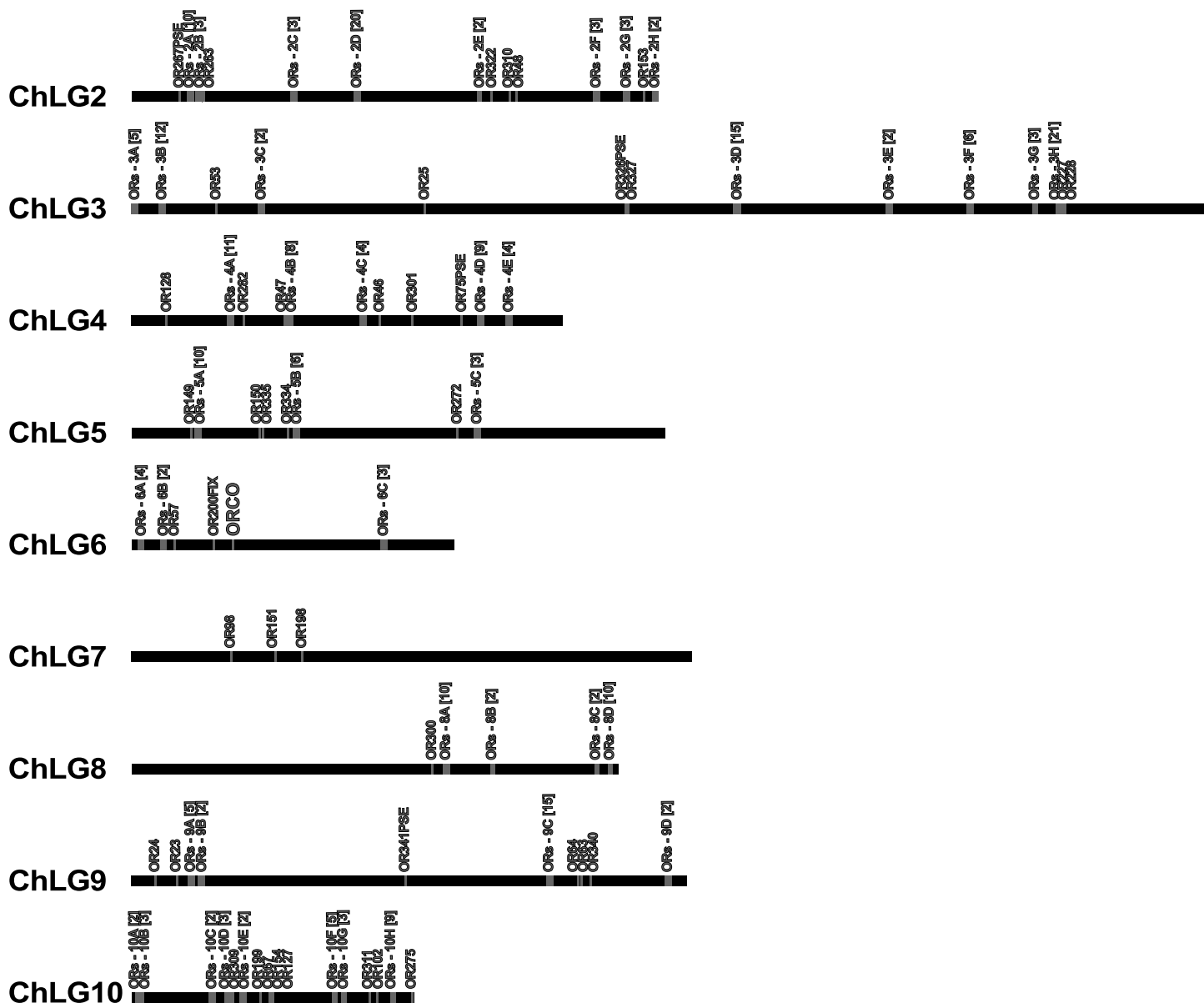

Supplement: Additional file 13: Figure S9. — OR gene tissue expression and their chromosomal localization. a Venn diagram showing the number of ORs expressed (RPKM ≥ 0.5) in the different body parts: antennae, legs, mouthparts (as piece of the head capsule anterior of the antennae), heads (the whole head capsule including mouthparts but excluding the antennae), and bodies (excluding head and legs). b Venn diagram comparing our results (yellow, green) with data from Engsontia et al. [115] (blue, red). Number of expressed ORs, defined by RPKM ≥ 0.5 (yellow), by RT-PCR (blue), not expressed RPKM < 0.5 (green), or with no RT-PCR amplicon (red). ORs of the brown group were not previously tested by Engsontia et al. c Chromosomal localization of T. castaneum ORs. Based on the Georgia GA-2 strain genome assembly 3.0 [81], only chromosomal linkage groups containing an IR or SNMP are depicted. Gene clusters are indicated by a number referring to the chromosome and a letter conveys the relative position on the chromosome. The number of genes within this cluster is indicated in the square brackets. (PDF 277 kb) [file 12915_2016_304_MOESM13_ESM.pdf]

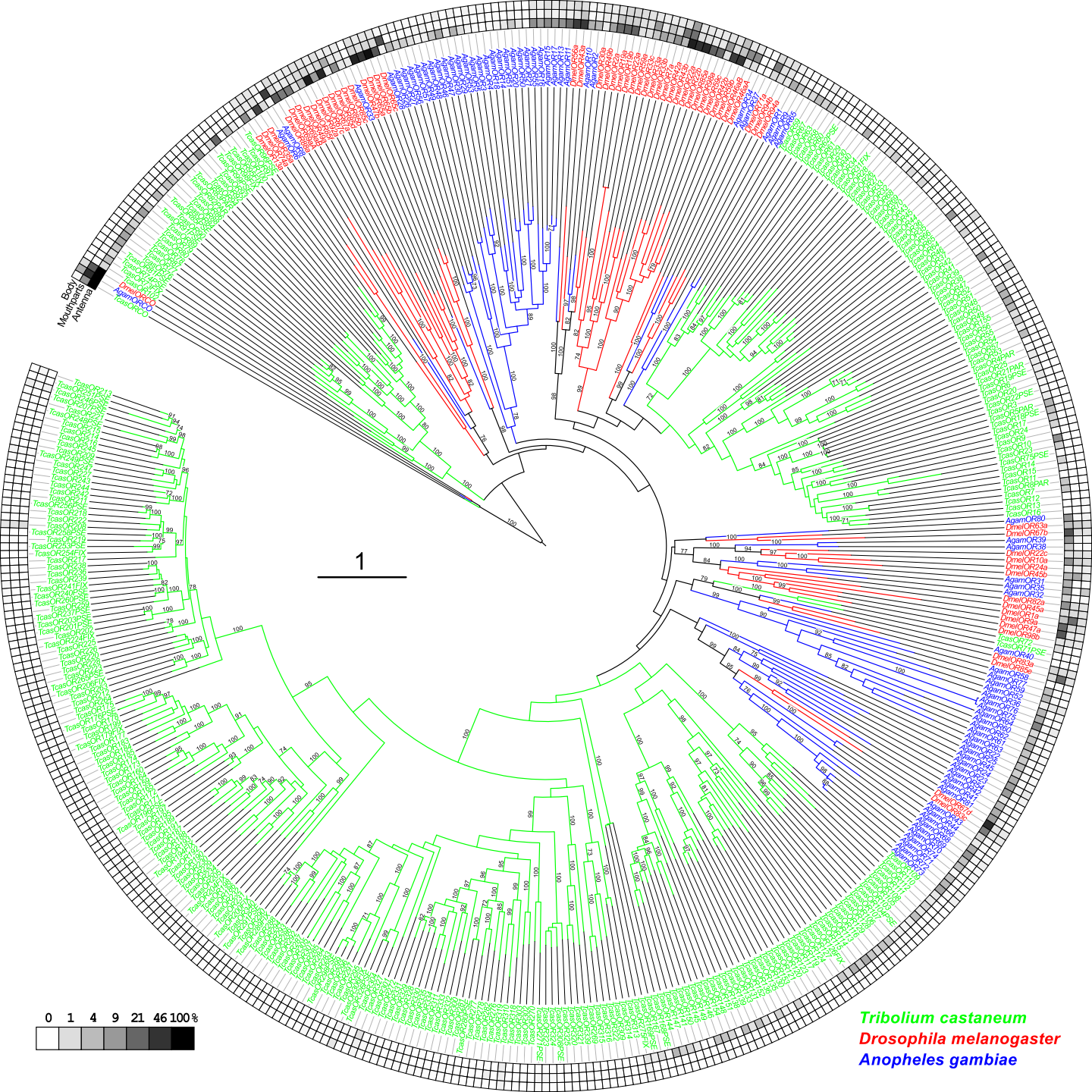

Supplement: Additional file 14: Figure S10. — Phylogenetic tree of the ORs based on protein sequences. Outer rings represent the expression in body, mouthparts (T. castaneum: palps, mandible, labrum, and labium; D. melanogaster: palp and proboscis; An. gambiae: maxillary palp) and antenna as a percentage compared to the highest expressed gene according to the scale in the left upper corner. Note that the methods used to obtain the different expression data (RNAseq and microarray) are not directly comparable. This figure can, thus, only give an impression of the tissue-specific abundance of the transcripts. The scale bars within the trees represent one amino acid substitution per site. (PDF 2302 kb) [file 12915_2016_304_MOESM14_ESM.pdf]

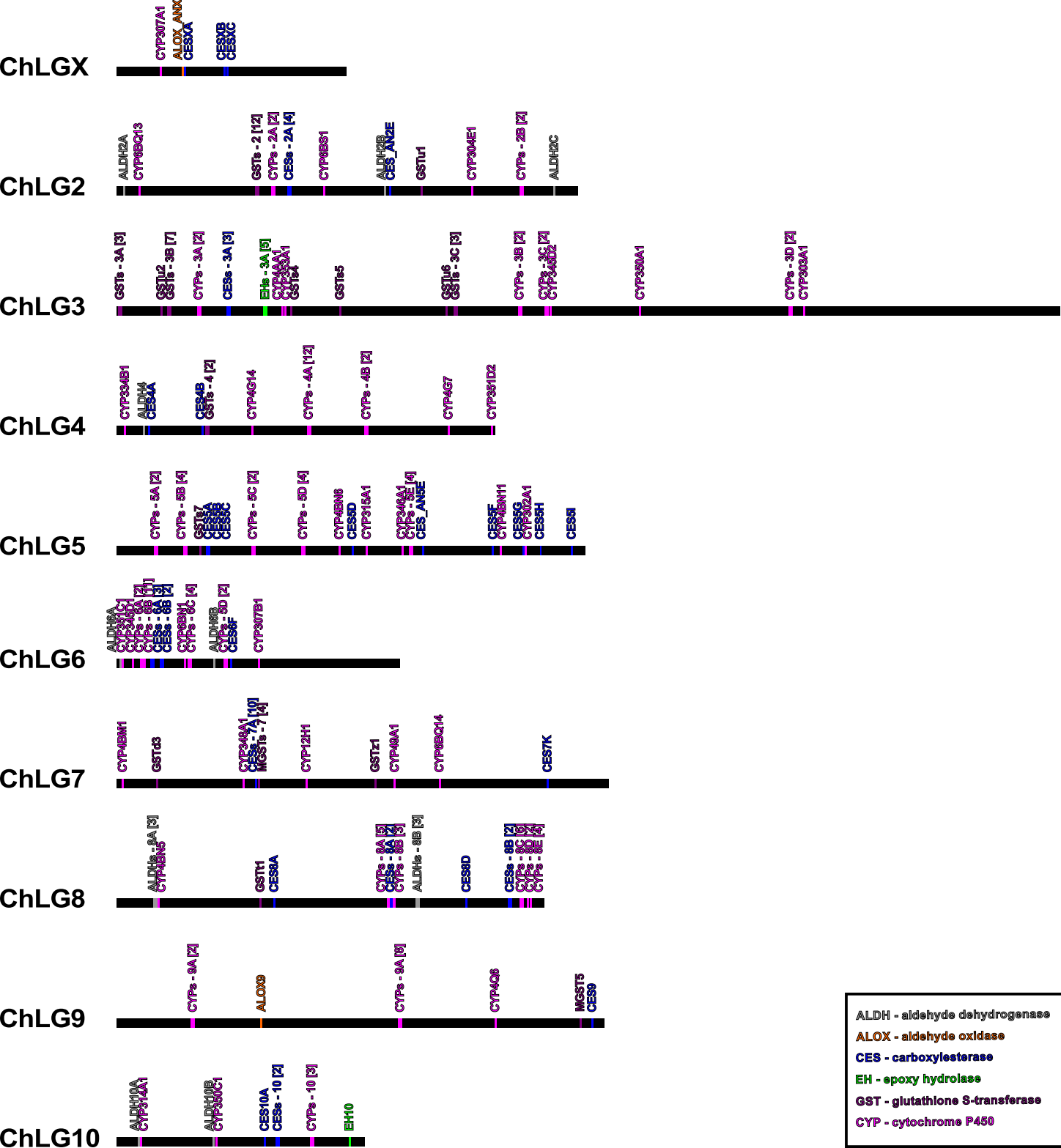

Supplement: Additional file 15: Figure S11. — Chromosomal localization of potential T. castaneum ODE genes. Based on Georgia GA-2 strain genome assembly 3.0 [81], aldehyde dehydrogenase (ALDH, in grey), aldehyde oxidase (ALOX, in orange), carboxylesterase (CES, in blue), epoxide hydrolase (EH, in green), glutathione S-transferase (GST, in purple), and cytochrome P450 (CYP, in magenta). Gene clusters are indicated by a number referring to the chromosome and a letter conveys the relative position on the chromosome. The number of genes within this cluster is indicated in the square brackets. (PDF 257 kb) [file 12915_2016_304_MOESM15_ESM.pdf]

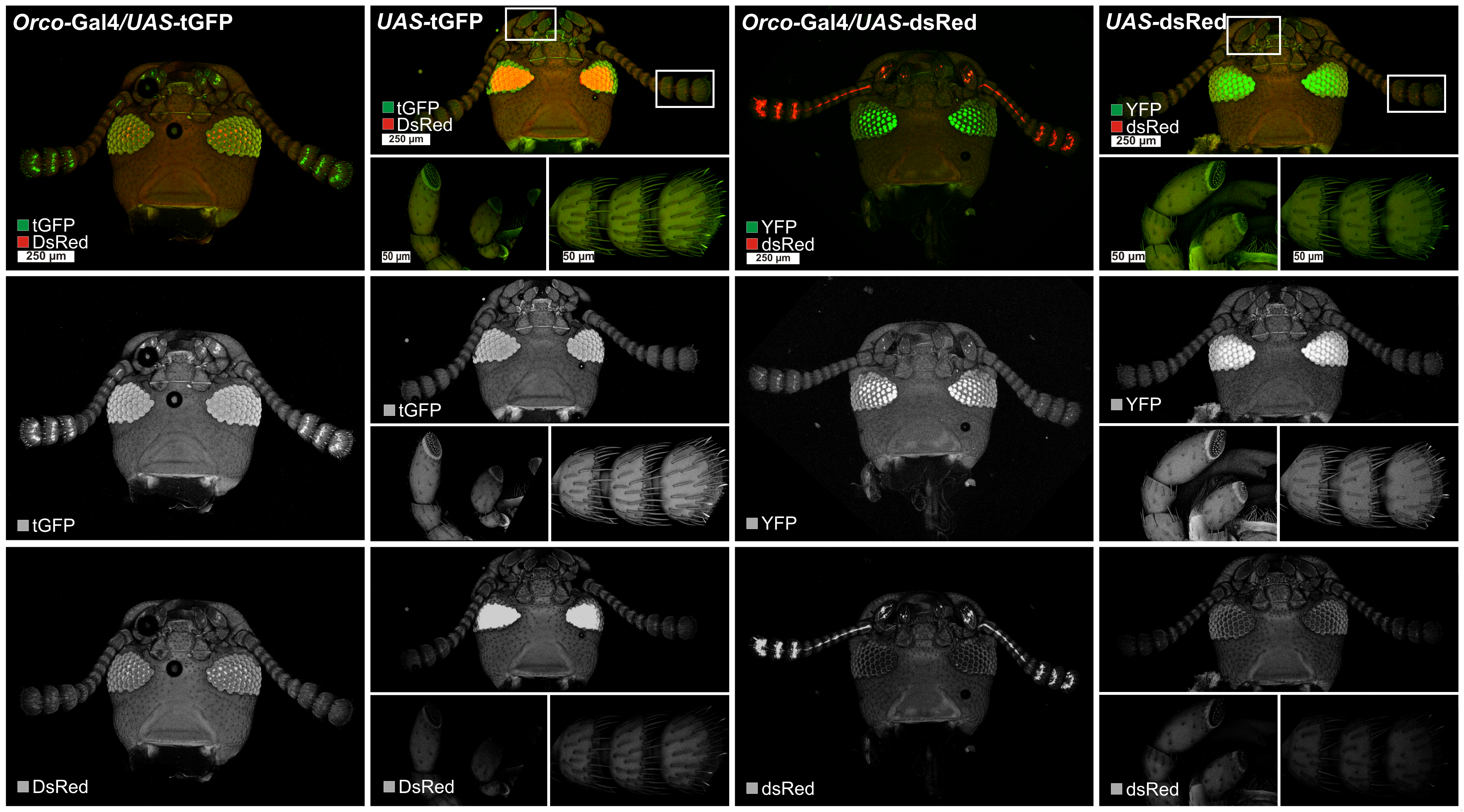

Supplement: Additional file 17: Figure S12. — UAS responder lines in the absence of Gal4 driver. In the four rows, the maximum projections of head capsules from different transgenic strains (Orco-Gal4/UAS-tGFP, UAS-tGFP, Orco-Gal4/UAS-dsRed, and UAS-dsRed) are depicted. The upper row represents the overlay of both channels (GFP/YFP in green and dsRed in red). For UAS responders without the Gal4 driver, high-resolution images of the palps and the antennal club are provided. In the second and third rows, the separated channels are given as greyscale images. The UAS-tGFP and UAS-dsRed lines do not show leaky reporter expression in the absence of a Gal4 driver in the antennae and palps. The presence of the genetic constructs is indicated by the eye markers: pBac[3XP3-dsRed_UAS-Tchsp68bP-tGFP-SV40] and pBac[3XP3-eYFP_UAS-Tchsp68bP-DsRedex-SV40]. The marker signal is quenched in the crossed lines by the vermillion rescue marker of the Orco-Gal4 construct pBac[3XP3-gVerm_2.5kbOrcoUp_GAL4delta]. (TIF 6507 kb) [file 12915_2016_304_MOESM17_ESM.tif]
