# Supplementary material for: Morphological and Transcriptomic Analysis of a Beetle Chemosensory System Reveals a Gnathal Olfactory Center
Source: BMC Biol. 2016 Oct 17;14:90. doi: 10.1186/s12915-016-0304-z (PMC5067906; doi:10.1186/s12915-016-0304-z)
Supplement: Additional file 11: Figure S7. — GR gene tissue expression and their chromosomal localization. a Venn diagram showing the number of GRs expressed (RPKM ≥ 0.5) in the different body parts: antennae, legs, mouthparts (as a piece of the head capsule anterior of the antennae), heads (the whole head capsule including mouthparts but excluding the antennae), and bodies (excluding head and legs). b Based on Georgia GA-2 strain genome assembly 3.0 [81], only chromosomal linkage groups containing an IR or SNMP are depicted. Gene clusters are indicated by a number referring to the chromosome and a letter conveys the relative position on the chromosome. The number of genes within this cluster is indicated in the square brackets. (PDF 176 kb) [file 12915_2016_304_MOESM11_ESM.pdf]

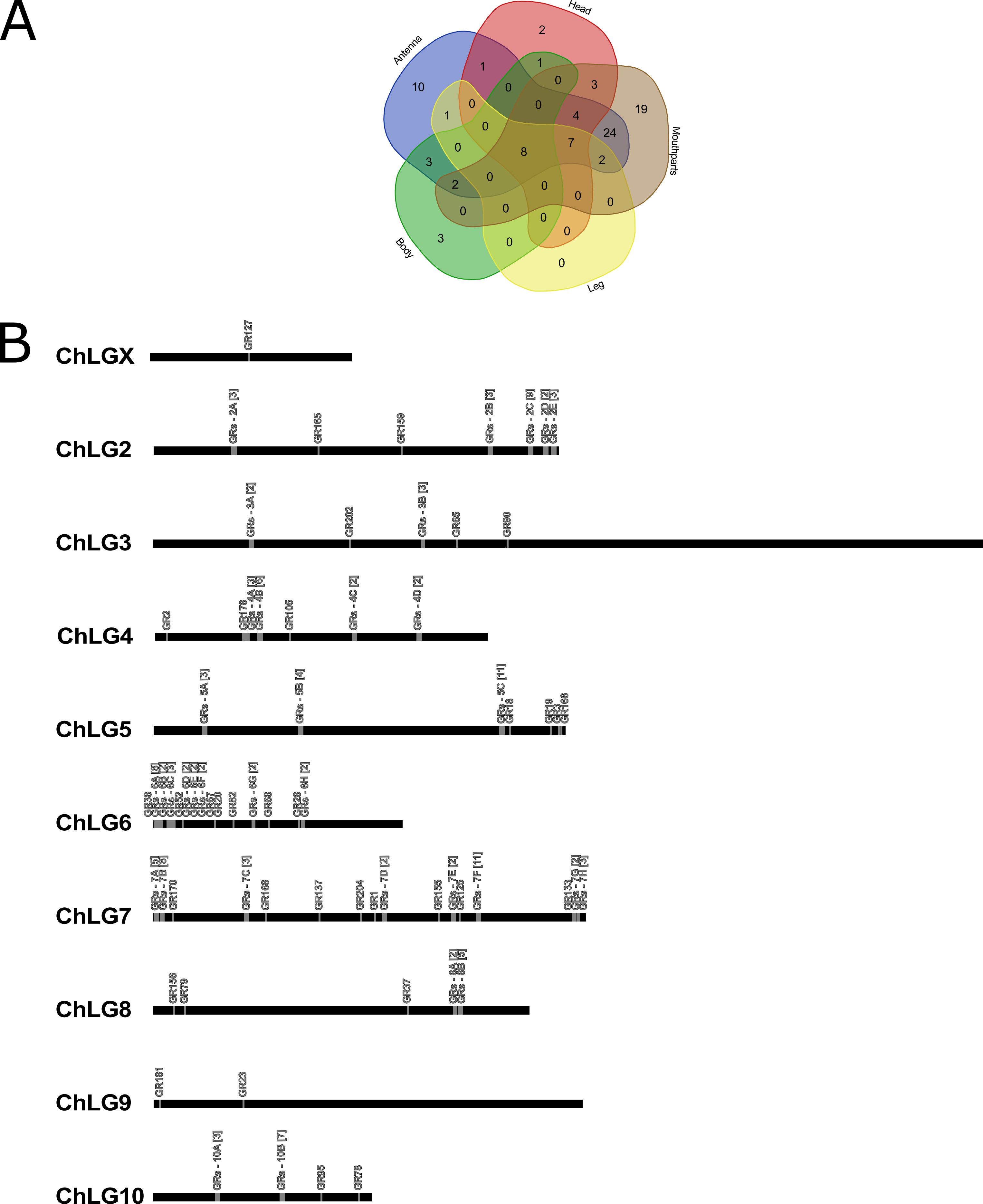

A
Antenna
Head
Mouthparts
Leg
Body
10
2
19
3
0
1
1
0
3
0
0
4
24
0
1
0
7
8
2
3
2
0
0
0
0
0
0
0
0
0
0
B
ChLGX
GR127
ChLG2
GRs - 2A [3]
GR165
GR159
GRs - 2B [3]
GRs - 2C [9]
GRs - 2D [2]
GRs - 2E [3]
ChLG3
GRs - 3A [2]
GR202
GRs - 3B [3]
GR65
GR90
ChLG4
GR2
GR178
GRs - 4A [3]
GRs - 4B [6]
GR105
GRs - 4C [2]
GRs - 4D [2]
ChLG5
GRs - 5A [3]
GRs - 5B [4]
GRs - 5C [11]
GR18
GR19
GR166
ChLG6
GR38
GRs - 6A [8]
GRs - 6B [2]
GRs - 6C [3]
GR52
GRs - 6D [2]
GRs - 6E [2]
GRs - 6F [2]
GR67
GR20
GR82
GRs - 6G [2]
GR68
GR28
GRs - 6H [2]
ChLG7
GRs - 7A [5]
GRs - 7B [8]
GR170
GRs - 7C [3]
GR168
GR137
GR204
GR1
GRs - 7D [2]
GR155
GRs - 7E [2]
GR125
GRs - 7F [11]
GR133
GRs - 7G [2]
GRs - 7H [3]
ChLG8
GR156
GR79
GR37
GRs - 8A [2]
GRs - 8B [5]
ChLG9
GR181
GR23
ChLG10
GRs - 10A [3]
GRs - 10B [7]
GR95
GR78
