# Supplementary material for: Morphological and Transcriptomic Analysis of a Beetle Chemosensory System Reveals a Gnathal Olfactory Center
Source: BMC Biol. 2016 Oct 17;14:90. doi: 10.1186/s12915-016-0304-z (PMC5067906; doi:10.1186/s12915-016-0304-z)
Supplement: Additional file 16: — Sequences of primers and template plasmid used to generate pSLfa1180[2.5kbOrcoUp_GAL4delta]. (PDF 111 kb) [file 12915_2016_304_MOESM16_ESM.pdf]

Primer:

Gal4deltafor: TATGGATCCACCTGCGCTGATGAAGCTACTGTCTTCTATCGAACAAG

SV40rev: GGTCCATGGGATGAGTTTGGACAAACCACAACTAG

TcOR1upfor: TAGGGATCCTTAAATTATAAAAACAATTGACGAAAAATAGCCTCGTC

TcOR1uprev: AGTACCTGCTTCATCATCTGGAAGTTGGTTAGAATTTGGTATTG

T7: gaattgtaatacgactcactatagg

T7-SP6: taatacgactcactataggatttaggtgacactataga

TcasOrco3UTRrev: TTAGGGTAAACTTTATTAGGTAAAATAGAAGTAACTAAGTC

TcasOrco3for: GCGTTAGCCCAAGTGTTTC

>TcasOrco

ATGATGAAATTCAAGGTCACGGGCCTCGTGGCCGACCTTATGCCCAACATTCGGCTCATCCAAGCGTC
CGGGCACTTCATGCTCAACTACCATGCCGATAATTCCGGCGCCTTGCACACCCTCAGGCTCGGCTACT
GCTGCATGCACCTGGTGTTCGTCCTAGTCCAATATGGTTGTAATTTCGTTAATTTGGTACTGGAACGT
GGTGACGTCAACGATTTGGCTGCCAACACCATCACGGTGCTGTTTTTCACGCATTGTGTCACGAAATT
CGTGTATTTCGCGGTCAGGTCGAAACTTTTCTACCGAACTTTAGGTATTTGGAACCAACCAAACAGTC
ATCCGTTGTTTGTTGAGTCGAATAATCGCTACCACGGCATTGCTTTGAAAAAAATGCGGCGCTTGCTC
TATATTATAATCATCTGGACGTCCTTCAGTGCTATTGCTTGGACCGGTATCACGTTCGTGGGTGATAG
CGTCCACAATATCAAGGACCCAGAAAACGAAAATTTGACCATCACTGAACCCATCCCACGCTTGTTGG
TCAAGGCATGGTACCCATGGGACGCCATGAGCGGCATGCCCTACTACATAACCCTCGTGTTCCAAATC
TACTACGTTTTTTTCTCACTCGCGCACGCAAACCTCCTCGATAGCCTCTTCTGTTCGTGGCTGATTTT
CGCCTGCGAGCAGTTGCAACACTTGAAAGAGATCATGAAGCCTTTGATGGAACTTTCGGCAACTCTGG
ACACCTACGTGCCGAAGAGTGCCGACTTGTTCCGAGCGCCGAGCGCCACCTCGCAGGACCAACTCATC
GAAAATGATTACAATGAGAAAAACGAGGATCTTAAGGGGGTGTATAGCACGCGCCAGGAGCTCGGCGG
CCATTTTAGGGGCGGGGCGTTGCAGAATTTCGGTAGTGGGGGCGTTGGGCCGAACGGTTTGACCAAAA
AACAGGAGTTGATGGTCAGGTCGGCGATCAAATACTGGGTGGAGCGCCACAAACACGTCGTCAGGTTG
GTCACGGCCATCGGTGATGCGTATGGTGTGGCACTCCTGCTCCATATGCTGACGTCTACGATCATGTT
GACGCTTTTGGCCTATCAGGCTACGAAAATCACAGGGGTGGACAAATACGCAGCTACGGTTCTGGGCT
ATTTGCTGTTTGCGTTAGCCCAAGTGTTTCATTTTTGTATTTTTGGCAACCGCCTCATTGAGGAGAGC
TCTTCGGTGATGGAGGCGGCCTATAGTTGCCATTGGTACGACGGGTCGGAGGAGGCTAAGACTTTTGT
ACAGATCGTCTGCCAACAATGTCAGAAGGCTATGTCCATTTCCGGTGCTAAATTCTTCACGATTTCGT
TAGATCTGTTCGCTTCGGTACTGGGCGCTGTGGTGACCTACTTCATGGTGTTGGTGCAACTCAAATGA
GGGGTTTTTCGTTGCGTACGGTTGGTAGTTCCTTAATTTCTGCACGTGACGTTTTGAAAATTGTGATAATTAGACGGCACTGATTTTTCAAGTT
ACACAGCATTCATAAGTGTCTTAATTCAAGATGGCGAGTACCTGATAATGGCGGTGACAAGGGGAAATAATAACGCTCTAGACTTAGTTACTTC
TATTTTACCTAATAAAGTTTACCCTAACTA

Plasmids:

>CH#757: **pSLfa_p-Gal4delta-SV40, UASp-lacZ-K10_fa**

CTAGAGTCGACGGTACCGGGCCCCAATTCCCGGGGATCGAAAGAGGCTGCTAAAGCAAAAAAGAAGTCACCATGT
CGTTTACTTTGACCAACAAGAACGTGATTTTCGTTGCCGGTCTGGGAGGCATTGGTCTGGACACCAGCAAGGAGC
TGCTCAAGCGCGATCCCGTCGTTTTACAACGTCGTGAGTGGGAAAACCCTGGCGTTACCCAACTTAATCGCCTTG

CAGCACATCCCCCTTTCGCCAGCTGGCGTAATAGCGAAGAGGCCCGCACCGATCGCCCTTCCCAACAGTTGCGCA
GCCTGAATGGCGAATGGCGCTTTGCCTGGTTTCCGGCACCAGAAGCGGTGCCGGAAAGCTGGCTGGAGTGCGATC
TTCCTGAGGCCGATACTGTCGTCGTCCCCTCAAACTGGCAGATGCACGGTTACGATGCGCCCATCTACACCAACG
TAACCTATCCCATTACGGTCAATCCGCCGTTTGTTCCCACGGAGAATCCGACGGGTTGTTACTCGCTCACATTTA
ATGTTGATGAAAGCTGGCTACAGGAAGGCCAGACGCGAATTATTTTTGATGGCGTTAACTCGGCGTTTCATCTGT
GGTGCAACGGGCGCTGGGTCGGTTACGGCCAGGACAGTCGTTTGCCGTCTGAATTTGACCTGAGCGCATTTTTAC
GCGCCGGAGAAAACCGCCTCGCGGTGATGGTGCTGCGTTGGAGTGACGGCAGTTATCTGGAAGATCAGGATATGT
GGCGGATGAGCGGCATTTTCCGTGACGTCTCGTTGCTGCATAAACCGACTACACAAATCAGCGATTTCCATGTTG
CCACTCGCTTTAATGATGATTTCAGCCGCGCTGTACTGGAGGCTGAAGTTCAGATGTGCGGCGAGTTGCGTGACT
ACCTACGGGTAACAGTTTCTTTATGGCAGGGTGAAACGCAGGTCGCCAGCGGCACCGCGCCTTTCGGCGGTGAAA
TTATCGATGAGCGTGGTGGTTATGCCGATCGCGTCACACTACGTCTGAACGTCGAAAACCCGAAACTGTGGAGCG
CCGAAATCCCGAATCTCTATCGTGCGGTGGTTGAACTGCACACCGCCGACGGCACGCTGATTGAAGCAGAAGCCT
GCGATGTCGGTTTCCGCGAGGTGCGGATTGAAAATGGTCTGCTGCTGCTGAACGGCAAGCCGTTGCTGATTCGAG
GCGTTAACCGTCACGAGCATCATCCTCTGCATGGTCAGGTCATGGATGAGCAGACGATGGTGCAGGATATCCTGC
TGATGAAGCAGAACAACTTTAACGCCGTGCGCTGTTCGCATTATCCGAACCATCCGCTGTGGTACACGCTGTGCG
ACCGCTACGGCCTGTATGTGGTGGATGAAGCCAATATTGAAACCCACGGCATGGTGCCAATGAATCGTCTGACCG
ATGATCCGCGCTGGCTACCGGCGATGAGCGAACGCGTAACGCGAATGGTGCAGCGCGATCGTAATCACCCGAGTG
TGATCATCTGGTCGCTGGGGAATGAATCAGGCCACGGCGCTAATCACGACGCGCTGTATCGCTGGATCAAATCTG
TCGATCCTTCCCGCCCGGTGCAGTATGAAGGCGGCGGAGCCGACACCACGGCCACCGATATTATTTGCCCGATGT
ACGCGCGCGTGGATGAAGACCAGCCCTTCCCGGCTGTGCCGAAATGGTCCATCAAAAAATGGCTTTCGCTACCTG
GAGAGACGCGCCCGCTGATCCTTTGCGAATACGCCCACGCGATGGGTAACAGTCTTGGCGGTTTCGCTAAATACT
GGCAGGCGTTTCGTCAGTATCCCCGTTTACAGGGCGGCTTCGTCTGGGACTGGGTGGATCAGTCGCTGATTAAAT
ATGATGAAAACGGCAACCCGTGGTCGGCTTACGGCGGTGATTTTGGCGATACGCCGAACGATCGCCAGTTCTGTA
TGAACGGTCTGGTCTTTGCCGACCGCACGCCGCATCCAGCGCTGACGGAAGCAAAACACCAGCAGCAGTTTTTCC
AGTTCCGTTTATCCGGGCAAACCATCGAAGTGACCAGCGAATACCTGTTCCGTCATAGCGATAACGAGCTCCTGC
ACTGGATGGTGGCGCTGGATGGTAAGCCGCTGGCAAGCGGTGAAGTGCCTCTGGATGTCGCTCCACAAGGTAAAC
AGTTGATTGAACTGCCTGAACTACCGCAGCCGGAGAGCGCCGGGCAACTCTGGCTCACAGTACGCGTAGTGCAAC
CGAACGCGACCGCATGGTCAGAAGCCGGGCACATCAGCGCCTGGCAGCAGTGGCGTCTGGCGGAAAACCTCAGTG
TGACGCTCCCCGCCGCGTCCCACGCCATCCCGCATCTGACCACCAGCGAAATGGATTTTTGCATCGAGCTGGGTA
ATAAGCGTTGGCAATTTAACCGCCAGTCAGGCTTTCTTTCACAGATGTGGATTGGCGATAAAAAACAACTGCTGA
CGCCGCTGCGCGATCAGTTCACCCGTGCACCGCTGGATAACGACATTGGCGTAAGTGAAGCGACCCGCATTGACC
CTAACGCCTGGGTCGAACGCTGGAAGGCGGCGGGCCATTACCAGGCCGAAGCAGCGTTGTTGCAGTGCACGGCAG
ATACACTTGCTGATGCGGTGCTGATTACGACCGCTCACGCGTGGCAGCATCAGGGGAAAACCTTATTTATCAGCC
GGAAAACCTACCGGATTGATGGTAGTGGTCAAATGGCGATTACCGTTGATGTTGAAGTGGCGAGCGATACACCGC
ATCCGGCGCGGATTGGCCTGAACTGCCAGCTGGCGCAGGTAGCAGAGCGGGTAAACTGGCTCGGATTAGGGCCGC
AAGAAAACTATCCCGACCGCCTTACTGCCGCCTGTTTTGACCGCTGGGATCTGCCATTGTCAGACATGTATACCC
CGTACGTCTTCCCGAGCGAAAACGGTCTGCGCTGCGGGACGCGCGAATTGAATTATGGCCCACACCAGTGGCGCG
GCGACTTCCAGTTCAACATCAGCCGCTACAGTCAACAGCAACTGATGGAAACCAGCCATCGCCATCTGCTGCACG
CGGAAGAAGGCACATGGCTGAATATCGACGGTTTCCATATGGGGATTGGTGGCGACGACTCCTGGAGCCCGTCAG
TATCGGCGGACCAGCTGAGCGCCGGTCGCTACCATTACCAGTTGGTCTGGTGTCAAAAATAATAATAACCGGGCA
GGCCATGTCTGCCCGTATTTCGCGTAAGGAAATCCATTATGTACTATTTAAAAAACACAAACTTTTGGATGTTCG
GTTTATTCTTTTTCTTTTACTTTTTTATCATGGGAGCCTACTTCCCGTTTTTCCCGATTTGGCTACATGACATCA
ACCATATCAGCAAAAGTGATACGGGTATTATTTTTGCCGCTATTTCTCTGTTCTCGCTATTATTCCAACCGCTGT
TTGGTCTGCTTTCTGACAAACTCGACTCTAGGGCCGCCTGCAGCTGGCGCCATCGATACGGTCGACCGTACGTCG
CGACCGCGGACATGTACAGAGCTCGAGAAGTACTAGAATTATACCTAAACCCATGGTCAAGAGTAAACATTTCTG
CCTTTGAAGTTGAGAACACAATTAAGCATCCCCTGGTTAAACCTGACACTTCATACTTGTTAATAGCGCCATAAA
CATAGCACCAATTTCGAAGAAATCAGTTAAAAGCAATTAGCAATTAGCAATTAGCAATAACTCTGCTGACTTCAA
AACGAGAAGAGTTGCAAGTATTTGTAAGGCACAGTTTATAGACCACCGACGGCTCATTAGGGCTCGTCATGTAAC
TAAGCGCGGTGAAACCCAATTGAACAGTAGTAGGTGGAATTAGTTAGTTAGTCAATGGGGAAGATTTAACCCTCA
GGTAGCAAAGTAATTGAACTGCGGGCAGAGAGTCCTAAGACTAAATAATATATTTAAAAATCTGGCCCTTTGACC
TTGCTTGTCAGGTGCATTTGGGTTCAATCGTAAGTTGCTTCTATATAAACACTTTCCCCATCCCCGCAATAATGA
AGAATACCGCAGAATAAAGAGAGATTTGCAACAAAAAATAAAGGCATTGCGAAAACTTTTTATGGGGGATCATTA
CACTCGGGCCTACGGTTACAATTCCCAGCCACTTAAGCGACAAGTTTGGCCAACAATCCATCTAATAGCTAATAG
CGCAATCACTGGTAATCGCAAGAGTATATAGGGCAATAGAACCCATGGATTTGACCAAAGGTAACCGAGACAATG

GAGAAGCAAGAGGATTTCAAACTGAACACCCACAGTGCTGTGTACTACCACTGGCGCGTTTGGAGCTCACTGGCC
TGATGCGCTCCTCCGGGCGTTTCAAGCCTGCTTTACGTGGTATACTCCATTACGGTCAAGTTGGTGGTCACCGTG
CTGTTTCCCTTGAGCTTGCTGGCCAGGCTGCTGTTCACCACCAACATGGCCGGATTGTGCGAGAACCTGACCATA
AGTATTACCGATATTGTGGCCAATTTGAAGTTTGCGAATGTGTACATGGTGAGGAAGCAGCTCCATGAGATTCGC
TCTCTCCTAAGGCTCATGGACGCTAGAGCCCGGCTGGTGGGCGATCCCGAGGAGATTTCTGCCTTGAGGAAGGAA
GTGAATATCGGCACAGGGCACTTTCCAGCACCTTTGGCCAGTATTTTCGTATTTGGCACTACTTTGAGTTCGCGT
CCGCGTGGTCGTTCGCCCGGATCGAGAGCTCCTGTATCCGGCCTGGTTCGGCGTTGACTGGATTCACTCCACCAG
AAACTATGTGCTCATCAATATCTACCAGCTCTTCGGCTTGATAGTGCAGGCTATACAGAACTGCGCTAGTGACTC
CTATCCGCCTGCGTTTCTCTGCCTGCTCACGGGTCATATGCGTGCTTTGGAGCTGAGGGTGCGGCGGATTGGCTG
CACTGCAGCTGGCGCCATCGATACGCGTACGTCGCGACCGCGGACATGTACAGAGCTCGAGAAGTACTAGTGGCC
ACGTGGGCCGTGCACCTTAAGCTTGGCGCGCCTAGGCCGGCCAGCTTGGCACTGGCCGTCGTTTTACAACGTCGT
GACTGGGAAAACCCTGGCGTTACCCAACTTAATCGCCTTGCAGCACATCCCCCTTTCGCCAGCTGGCGTAATAGC
GAAGAGGCCCGCACCGATCGCCCTTCCCAACAGTTGCGCAGCCTGAATGGCGAATGGCGCCTGATGCGGTATTTT
CTCCTTACGCATCTGTGCGGTATTTCACACCGCATACGTCAAAGCAACCATAGTACGCGCCCTGTAGCGGCGCAT
TAAGCGCGGCGGGTGTGGTGGTTACGCGCAGCGTGACCGCTACACTTGCCAGCGCCCTAGCGCCCGCTCCTTTCG
CTTTCTTCCCTTCCTTTCTCGCCACGTTCGCCGGCTTTCCCCGTCAAGCTCTAAATCGGGGGCTCCCTTTAGGGT
TCCGATTTAGTGCTTTACGGCACCTCGACCCCAAAAAACTTGATTTGGGTGATGGTTCACGTAGTGGGCCATCGC
CCTGATAGACGGTTTTTCGCCCTTTGACGTTGGAGTCCACGTTCTTTAATAGTGGACTCTTGTTCCAAACTGGAA
CAACACTCAACCCTATCTCGGGCTATTCTTTTGATTTATAAGGGATTTTGCCGATTTCGGCCTATTGGTTAAAAA
ATGAGCTGATTTAACAAAAATTTAACGCGAATTTTAACAAAATATTAACGTTTACAATTTTATGGTGCACTCTCA
GTACAATCTGCTCTGATGCCGCATAGTTAAGCCAGCCCCGACACCCGCCAACACCCGCTGACGCGCCCTGACGGG
CTTGTCTGCTCCCGGCATCCGCTTACAGACAAGCTGTGACCGTCTCCGGGAGCTGCATGTGTCAGAGGTTTTCAC
CGTCATCACCGAAACGCGCGAGACGAAAGGGCCTCGTGATACGCCTATTTTTATAGGTTAATGTCATGATAATAA
TGGTTTCTTAGACGTCAGGTGGCACTTTTCGGGGAAATGTGCGCGGAACCCCTATTTGTTTATTTTTCTAAATAC
ATTCAAATATGTATCCGCTCATGAGACAATAACCCTGATAAATGCTTCAATAATATTGAAAAAGGAAGAGTATGA
GTATTCAACATTTCCGTGTCGCCCTTATTCCCTTTTTTGCGGCATTTTGCCTTCCTGTTTTTGCTCACCCAGAAA
CGCTGGTGAAAGTAAAAGATGCTGAAGATCAGTTGGGTGCACGAGTGGGTTACATCGAACTGGATCTCAACAGCG
GTAAGATCCTTGAGAGTTTTCGCCCCGAAGAACGTTTTCCAATGATGAGCACTTTTAAAGTTCTGCTATGTGGCG
CGGTATTATCCCGTATTGACGCCGGGCAAGAGCAACTCGGTCGCCGCATACACTATTCTCAGAATGACTTGGTTG
AGTACTCACCAGTCACAGAAAAGCATCTTACGGATGGCATGACAGTAAGAGAATTATGCAGTGCTGCCATAACCA
TGAGTGATAACACTGCGGCCAACTTACTTCTGACAACGATCGGAGGACCGAAGGAGCTAACCGCTTTTTTGCACA
ACATGGGGGATCATGTAACTCGCCTTGATCGTTGGGAACCGGAGCTGAATGAAGCCATACCAAACGACGAGCGTG
ACACCACGATGCCTGTAGCAATGGCAACAACGTTGCGCAAACTATTAACTGGCGAACTACTTACTCTAGCTTCCC
GGCAACAATTAATAGACTGGATGGAGGCGGATAAAGTTGCAGGACCACTTCTGCGCTCGGCCCTTCCGGCTGGCT
GGTTTATTGCTGATAAATCTGGAGCCGGTGAGCGTGGGTCTCGCGGTATCATTGCAGCACTGGGGCCAGATGGTA
AGCCCTCCCGTATCGTAGTTATCTACACGACGGGGAGTCAGGCAACTATGGATGAACGAAATAGACAGATCGCTG
AGATAGGTGCCTCACTGATTAAGCATTGGTAACTGTCAGACCAAGTTTACTCATATATACTTTAGATTGATTTAA
AACTTCATTTTTAATTTAAAAGGATCTAGGTGAAGATCCTTTTTGATAATCTCATGACCAAAATCCCTTAACGTG
AGTTTTCGTTCCACTGAGCGTCAGACCCCGTAGAAAAGATCAAAGGATCTTCTTGAGATCCTTTTTTTCTGCGCG
TAATCTGCTGCTTGCAAACAAAAAAACCACCGCTACCAGCGGTGGTTTGTTTGCCGGATCAAGAGCTACCAACTC
TTTTTCCGAAGGTAACTGGCTTCAGCAGAGCGCAGATACCAAATACTGTTCTTCTAGTGTAGCCGTAGTTAGGCC
ACCACTTCAAGAACTCTGTAGCACCGCCTACATACCTCGCTCTGCTAATCCTGTTACCAGTGGCTGCTGCCAGTG
GCGATAAGTCGTGTCTTACCGGGTTGGACTCAAGACGATAGTTACCGGATAAGGCGCAGCGGTCGGGCTGAACGG
GGGGTTCGTGCACACAGCCCAGCTTGGAGCGAACGACCTACACCGAACTGAGATACCTACAGCGTGAGCTATGAG
AAAGCGCCACGCTTCCCGAAGGGAGAAAGGCGGACAGGTATCCGGTAAGCGGCAGGGTCGGAACAGGAGAGCGCA
CGAGGGAGCTTCCAGGGGGAAACGCCTGGTATCTTTATAGTCCTGTCGGGTTTCGCCACCTCTGACTTGAGCGTC
GATTTTTGTGATGCTCGTCAGGGGGGCGGAGCCTATGGAAAAACGCCAGCAACGCGGCCTTTTTACGGTTCCTGG
CCTTTTGCTGGCCTTTTGCTCACATGTTCTTTCCTGCGTTATCCCCTGATTCTGTGGATAACCGTATTACCGCCT
TTGAGTGAGCTGATACCGCTCGCCGCAGCCGAACGACCGAGCGCAGCGAGTCAGTGAGCGAGGAAGCGGAAGAGC
GCCCAATACGCAAACCGCCTCTCCCCGCGCGTTGGCCGATTCATTAATGCAGCTGGCACGACAGGTTTCCCGACT
GGAAAGCGGGCAGTGAGCGCAACGCAATTAATGTGAGTTAGCTCACTCATTAGGCACCCCAGGCTTTACACTTTA
TGCTTCCGGCTCGTATGTTGTGTGGAATTGTGAGCGGATAACAATTTCACACAGGAAACAGCTATGACCATGATT
ACGAATTGGCGCGCCTAGGCCGGCCGAATTCGAATGGCCATGGGACGTCGAGTCGATAGCCGAAGCTTACCGAAG
TATACACTTAAATTCAGTGCACGTTTGCTTGTTGAGAGGAAAGGTTGTGTGCGGACGAATTTTTTTTTGAAAACC

GGTGATAGAGCCTGAACCAGAAAAGATAAAAGAAGGCTATACCAGTGGGAGTACACAAACAGAGTAAGTTTGAAT
AGTAAAAAAAATCATTTATGTAAACAATAACGTGACTGTGCGTTAGGTCCTGTTCATTGGTACCNNNNNNNNNNN
NNNNNNNNNNNNNNNNATGAAGCTACTGTCTTCTATCGAACAAGCATGCGATATTTGCCGACTTAAAAAGCTCAA
GTGCTCCAAAGAAAAACCGAAGTGCGCCAAGTGTCTGAAGAACAACTGGGAGTGTCGCTACTCTCCCAAAACCAA
AAGGTCTCCGCTGACTAGGGCACATCTGACAGAAGTGGAATCAAGGCTAGAAAGACTGGAACAGCTATTTCTACT
GATTTTTCCTCGAGAAGACCTTGACATGATTTTGAAAATGGATTCTTTACAGGATATAAAAGCATTGTTAACAGG
ATTATTTGTACAAGATAATGTGAATAAAGATGCCGTCACAGATAGATTGGCTTCAGTGGAGACTGATATGCCTCT
AACATTGAGACAGCATAGAATAAGTGCGACATCATCATCGGAAGAGAGTAGTAACAAAGGTCAAAGACAGTTGAC
TGTATCGATTGAATTCCTCGGCAGCTCATCATGATAACTCCACAATTCCGTTGGATTTTATGCCCAGGGATGCTC
TTCATGGATTTGATTGGTCTGAAGAGGATGACATGTCGGATGGCTTGCCCTTCCTGAAAACGGACCCCAACAATA
ATGGGTTCTTTGGCGACGGTTCTCTCTTATGTATTCTTCGATCTATTGGCTTTAAACCGGAAAATTACACGAACT
CTAACGTTAACAGGCTCCCGACCATGATTACGGATAGATACACGTTGGCTTCTAAGATCCACAACATCCCGTTTA
CTTCAAAGTTATCTCAATAATTTTCACCCCTACTGCCCTATCGTGCACTCACCGACGCTAATGATGTTGTATAAT
AACCAGATTGAAATCGCGTCGAAGGATCAATGGCAAATCCTTTTTAACTGCATATTAGCCATTGGAGCCTGGTGT
ATAGAGGGGGAATCTACTGATATAGATGTTTTTTACTATCAAAATGCTAAATCTCATTTGACGAGCAAGGTCTTC
GAGTCAGGTTCCATAATTTTGGTGACAGCCCTACATCTTCTGTCGCGATATACACAGTGGAGGCAGAAAACAAAT
ACTAGCTATAATTTTCACAGCTTTTCCATANNNNNNNNNAAGCTTNGTCGACNNGGTACCGCGGCCAATTCGAATG
GCCATGGGCCGCGACTCTAGATCATAATCAGCCATACCACATTTGTAGAGGTTTTACTTGCTTTAAAAAACCTCC
CACACCTCCCCCTGAACCTGAAACATAAAATGAATGCAATTGTTGTTGTTAACTTGTTTATTGCAGCTTATAATG
GTTACAAATAAAGCAATAGCATCACAAATTTCACAAATAAAGCATTTTTTTCACTGCATTCTAGTTGTGGTTTGT
CCAAACTCATCAATGTATCTTAACATGGGACGTCGAGGCCGCTCTAGCCCCCCCTCGAATGTTCTCTCTCTTCTC
TTCTCTCTCTCTTTCTCGAATGTTCTCTCTCTTCTCTTCTCTCTCTCTTTCTCGAGGTCATCAAGCTTAGGCCTC
CAAGGCGGAGTACTGTCCTCCGGGCTGGCGGAGTACTGTCCTCCGGCAAGGCGGAGTACTGTCCTCCGGGCTGGC
GGAGTACTGTCCTCCGGCAAGGCGGAGTACTGTCCTCCGGGCTGGCGGAGTACTGTCCTCCGGCAAGGCGGAGTA
CTGTCCTCCGGGCTGGCGGAGTACTGTCCTCCGGCAAGGCGGAGTACTGTCCTCCGGGCTGGCGGAGTACTGTCC
TCCGGCAAGGCGGAGTACTGTCCTCCGGGCTGGCGGAGTACTGTCCTCCGGCAAGGCGGAGTACTGTCCTCCGGG
CTGGCGGAGTACTGTCCTCCGGCAAGGGTCGAGTCGATAGCCGAAGCTTACCGAAGTATACACTTAAATTCAGTG
CACGTTTGCTTGTTGAGAGGAAAGGTTGTGTGCGGACGAATTTTTTTTTGAAAACCGGTGATAGAGCCTGAACCA
GAAAAGATAAAAGAAGGCTATACCAGTGGGAGTACACAAACAGAGTAAGTTTGAATAGTAAAAAAAATCATTTAT
GTAAACAATAACGTGACTGTGCGTTAGGTCCTGTTCATTGGTAC
