# Supplementary material for: Morphological and Transcriptomic Analysis of a Beetle Chemosensory System Reveals a Gnathal Olfactory Center
Source: BMC Biol. 2016 Oct 17;14:90. doi: 10.1186/s12915-016-0304-z (PMC5067906; doi:10.1186/s12915-016-0304-z)
Supplement: Additional file 18: Table S2. — Primary and secondary antibodies and dyes used with additional information such as source and specificity. n/a not available. (PDF 118 kb) [file 12915_2016_304_MOESM18_ESM.pdf]

Primary antibodies					
Name	Abbreviation	Host Species	Used dilution	Donor and reference / vendor (catalog #, batch #, RRID #)	Specificity
D. melanogaster Synapsin I	α-Synapsin	Mouse	1/50	Dr. E. Buchner, University of Würzburg, Germany; Klagges et al., 1996 [245] (n/a, n/a, AB_2313617)	Utz et al., 2008 [246]
Moth Odorant receptor coreceptor	α-Orco	Rabbit	1/500	Dr. J. Krieger, Martin-Luther-Universität Halle-Wittenberg, Germany	RNAi Additional File 2 B and C
Locusta migratoria Tachykinin II	α-TKRP	Rabbit	1/10000	Dr. H. Agricola University of Jena, Germany; Veenstra et al., 1995 [247] / Jena Bioscience, Jena, Germany (ABD-045, n/a, AB_2341129)	Binzer et al., 2014 [103]
Red fluorescent protein	α-DsRed	Chicken	1/2000	Rockland Immunochemicals INC, Limerick, PA, USA (600-901-379, 26274, AB_10704808)	
Red fluorescent protein	α-DsRed	Rat	1/1000	ChromoTek GmbH, Planegg-Martinsried, Germany (rfp-antibody-5f8, 090428, AB_2336064)	
Turbo Green fluorescent protein	α-tGFP	Rabbit	1/8000	Evrogen, Moscow, Russia (AB514, n/a, n/a)	

Secondary antibodies				
Name	Abbreviation	Coupled dye	Used dilution	Donor/source, reference
goat anti-rabbit	GAR	Cy3	1/300	Jackson ImmunoResearch; Westgrove, PA, USA (111-165-144, n/a, AB_2338006)
goat anti-chicken	GAC	Cy3	1/300	Jackson ImmunoResearch; Westgrove, PA, USA (103-165-155, 93117, AB_2337386)
goat anti-rabbit	GAR	Cy2	1/300	Jackson ImmunoResearch; Westgrove, PA, USA (111-225-003, 88408, AB_2307385)
goat anti-rabbit	GAR	Cy5	1/300	Jackson ImmunoResearch; Westgrove, PA, USA (111-175-144, 76449, AB_2338013)
goat anti-mouse	GAM	Cy5	1/300	Jackson ImmunoResearch; Westgrove, PA, USA (115-175-146, 81431, AB_2338713)
goat anti-chicken	GAC	Alexa488	1/300	Jackson ImmunoResearch; Westgrove, PA, USA (103-547-008, n/a, n/a)
goat anti-rat	GARat	Cy5	1/300	Jackson ImmunoResearch; Westgrove, PA, USA (112-175-143, 69074, AB_2338263)

Dyes and tracers				
Name	Abbreviation	stains	Dilution	Donor / source / reference
4‘,6-Diamidin-2-phenylindol	DAPI	nuclei	1/20000	Sigma-Aldrich, Taufkirchen, Germany
Alexa Fluor 488 coupled phalloidin	Phalloidin	f-actin	1/200	Molecular Probes, Eugene, OR, USA
Neurobiotin		neurotracer	4% solution	Vector Laboratories, Burlingame, UK
Texas-Red coupled 3000 MW dextran		neurotracer	50 mg/ml or crystals	Molecular Probes, Invitrogen, Karlsruhe, Germany
Biotin coupled 3000 MW dextran		neurotracer	crystals	Molecular Probes, Invitrogen, Karlsruhe, Germany
Cy3 coupled Streptavidin		biotin	1/200	Dianova, Hamburg, Germany

References:

245. Klagges BRE, Heimbeck G, Godenschwege TA, Hofbauer A, Pflugfelder GO, Reifegerste R, et al. Invertebrate Synapsins: A Single Gene Codes for Several Isoforms in Drosophila. J. Neurosci. 1996;16:3154–65.

246. Utz S, Huetteroth W, Vömel M, Schachtner J. Mas-allatotropin in the developing antennal lobe of the sphinx moth Manduca sexta: Distribution, time course, developmental regulation, and colocalization with other neuropeptides. Dev. Neurobiol. 2008;68:123–42.

247. Veenstra JA, Lau GW, Agricola HJ, Petzel DH. Immunohistological localization of regulatory peptides in the midgut of the female mosquito Aedes aegypti. Histochem. Cell Biol. 1995;104:337–47.
